# Easy Access to Fused Tricyclic Quinoline Derivatives through Metal-Free Electrocatalytic [4 + 2] Annulation

**DOI:** 10.1021/acsorginorgau.4c00037

**Published:** 2024-08-08

**Authors:** Sayan Ghosh, Samrat Mallick, Devika Karolly, Suman De Sarkar

**Affiliations:** Department of Chemical Sciences, Indian Institute of Science Education and Research Kolkata, Mohanpur, West Bengal 741246, India

**Keywords:** electrocatalysis, redox, quinoline, [4 + 2] annulation, metal-free synthesis, fused-heterocycle, lactone

## Abstract

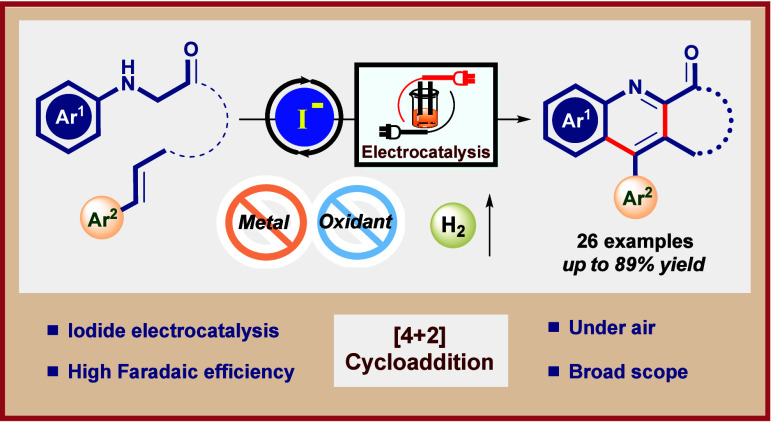

An efficient electrocatalytic cycloaddition approach for the construction of a lactone- or lactam-fused quinoline framework is documented. Diverse arrays of functionalities are well-compatible under this metal-free, mild, and scalable electro-redox protocol. Mechanistic studies indicate an iodide-mediated electro-oxidation of secondary amines to their corresponding imines and consequent [4 + 2] cycloaddition, fabricating C–C bonds followed by rapid aromatization leading to the six-membered core structure.

Fused quinolines represent a class of organic motifs that are immensely precious in medicinal chemistry because of their omnipresence in natural bioactive molecules and pharmaceuticals (Figure 1).^1,2^ Especially, quinoline-fused lactones and lactams are recognized as crucial intermediates or precursors for the fruitful synthesis of several eminent drug molecules and radioligands.^3−6^ Due to its versatile utility profile, considerable attention has been paid to constructing these important quinolines through diverse synthetic protocols.

**Figure 1:**
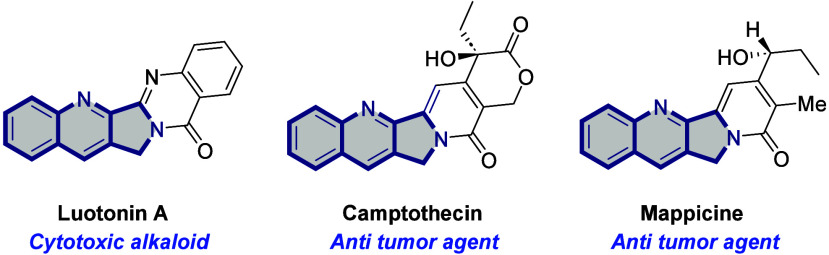
Representative examples of bioactive fused quinolines.

Since the pioneering works of Povarov in 1967,^7^ aza [4 + 2] cycloaddition reactions between amines and electron-rich olefins or alkynes have become a practical and efficient synthetic tool to fabricate substituted quinolines (Scheme 1a).^8−11^ This cycloaddition strategy has also been applied to build fused quinoline architectures, where both the amine and olefin moieties are embedded in a single molecular framework (Scheme 1b).^12−14^ Despite being strategically formidable, these modern synthetic approaches require either expensive catalysts or stoichiometric oxidants for the *in situ* conversion of secondary amines to their corresponding imines to execute the cycloaddition. Moreover, the involvement of such metal catalysts and super stoichiometric oxidants is often responsible for the generation of unwanted toxic chemical wastes and abridged cost-effectivity of the overall process. Photoredox catalysis, to some extent, circumvented these shortcomings;^15,16^ nonetheless, it still suffers from limitations as late-row transition metal-based photocatalysts or complex organic dyes are essential to carry out these transformations.

**Scheme 1:**
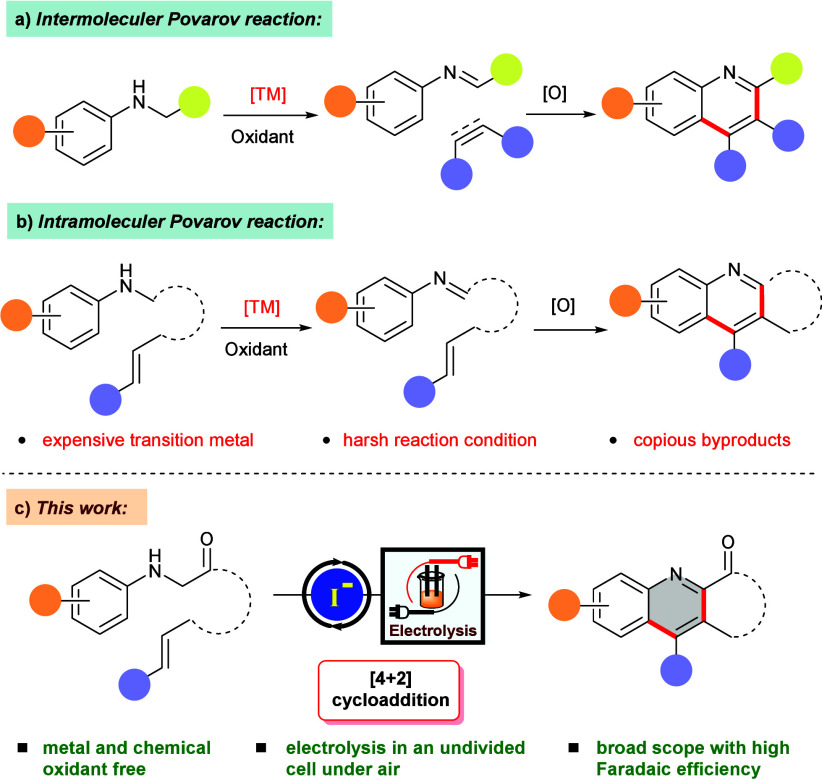
[4 + 2] Cycloaddition Strategies to Construct Heterocycles

In recent years, organic electrosynthesis has emerged as a true substitute to the traditional reagent-based synthetic approaches because of its atom-economic and sustainable features.^17−19^ During electrolysis, organic frameworks directly oxidize or reduce on the electrode surface, negating the need for expensive catalysts or stoichiometric quantity of redox reagents.^20−23^ Moreover, additional flexibility in regulating the reaction parameters (e.g., applied potential or electricity) is capable of providing superior chemoselectivity through controlled redox transformations.^24−26^

Oxidation of amines to their corresponding transient imine intermediates and further utilization in constructing diverse valuable molecular scaffolds is a prevalent approach in electro-organic synthesis.^27−29^ In this regard, α-amino carbonyls have been recognized as one of the suitable candidates due to their superior reactivity and easy availability from the ready feedstock.^30−32^ Recently, our group revealed a [3 + 2] cycloaddition protocol between α-amino carbonyls and tosylmethyl isocyanide, fabricating 1,5-disubstituted imidazole scaffolds.^33^ Iodide has proven to be a versatile and economical mediator in organic electrosynthesis and enabled various C–C and C–het bond-forming transformations.^34−37^ Comprehending the latest developments in this specific domain and relying on our proficiency with electro-organic transformations,^38−40^ herein we unveil an iodide-mediated electrochemical [4 + 2] cycloaddition protocol, illustrating the construction of lactone- and lactam-fused quinoline frameworks (Scheme 1c).

We commenced the inspection by electrolyzing a solution of cinnamyl 2-(*p*-tolylamino)acetate (**A1**) in the presence of 25 mol % tetrabutylammonium iodide (^*n*^Bu_4_NI) as the electro-catalyst and lithium perchlorate as the supporting electrolyte in acetonitrile solvent in an undivided electrochemical cell utilizing graphite and Pt plates as anode and cathode, respectively. To our delight, after the solution was electrolyzed at room temperature for 4 h under open air, the desired fused quinoline scaffold **B1** was obtained with 38% yield (Table 1, entry 1). Encouraged by this initial outcome, reaction parameters were screened in detail to obtain the optimal reaction conditions. Raising the reaction temperature to 65 °C proved to be beneficial, as the desired **B1** was obtained with the highest yield of 86% (entry 2). Notably, yield was substantially diminished in the absence of ^*n*^Bu_4_NI, establishing its effective role as the mediator (entry 3). Additional variation in the ^*n*^Bu_4_NI quantity did not lead to any significant improvement (entry 4). Employing ^*n*^Bu_4_NI as both a redox mediator and an electrolyte further deprived productivity (entry 5). Other iodide or bromide salts as mediators have also proven to be less competent (see Supporting Information for details). Afterward, changing the electrolyte to ^*n*^Bu_4_NPF_6_ or ^*n*^Bu_4_NOTs from LiClO_4_ produced subpar results (entries 6 and 7). Thereafter, the impact of the solvent on product conversion was scrutinized, and other solvents were found to be incompetent for the desired transformation (entries 8 and 9). Next, the effect of cathode material was surveyed, and altering the cathode to graphite or nickel foam resulted in inferior outcomes (entry 10). Also, Pt or glassy carbon as anode was incompetent for the product conversion (see Supporting Information for details). Raising or lowering the flow of electricity in the reaction medium failed to improve the reaction efficiency (entry 11). Finally, no product formation was detected without electricity (entry 12), establishing its essential requirement for desired annulation.

**Table 1:**
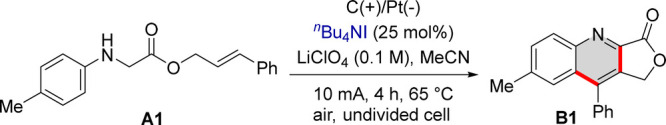
Optimization of the Reaction Conditions^a^

Entry	Deviation from standard conditions	Yield (%)^b^
1	at room temperature	38
**2**	**none**	**86**
3	without ^*n*^Bu_4_NI	40
4	5/30/50 mol % of ^*n*^Bu_4_NI	62/83/74
5	2 equiv, of ^*n*^Bu_4_NI	32^c^
6	^*n*^Bu_4_NPF_6_ instead of LiClO_4_	42
7	^*n*^Bu_4_NOTs instead of LiClO_4_	19
8	EtOH/MeOH as solvent	nd/nd
9	DMF/DMSO as solvent	71/nd
10	C/Ni foam as cathode	23/42
11	20 mA for 2 h/5 mA for 8 h	48/57
12	without electricity	nd

a Reaction conditions: **A1** (0.2 mmol), ^*n*^Bu_4_NI (0.05 mmol), LiClO_4_ (0.1 M) in MeCN, current = 10 mA, at 65 °C under air for 4 h.

b Isolated yield.

c Without LiClO_4_ as supporting electrolyte. nd = Not detected.

Once the optimized condition of this [4 + 2] cycloaddition protocol was obtained, the functional group tolerability was surveyed through the variation of the *N*-aryl counterpart (Table 2). Keeping the cinnamyl group constant at the ester handle, we first varied the alkyl substituents present in the *N*-aryl system, and compounds **B1** to **B4** were manufactured with excellent productivity. Next, halogen substitutions on the aromatic ring were investigated, which afforded the desired lactone-fused quinolines containing -F (**B5**), -Cl (**B6**), and -Br (**B7**) substituents with admirable yields. Electron-rich aryls accommodating -OPh (**B8**), -OCF_3_ (**B9**), and -SMe (**B10**) functionalities were found to be well-compatible in this oxidative environment. An electron-deficient aryl ring having a -CN group at the para position (**A11**) also converted smoothly to quinoline **B11** with an acceptable yield of 69%. Finally, the scope for disubstituted aromatic scaffolds was probed. Notably, 2,4-disubstituted aniline system **A12** delivered single isomer **B12**, and 3,4-disubstituted precursor **A13** generated a mixture of regioisomers **B13** and **B13’** with the ratio of 1.33:1. In order to demonstrate the practical applicability and industrial compatibility of this electrochemical strategy, a scale-up synthesis was performed, which delivered quinoline **B1** with an acceptable productivity (Table 2).

**Table 2:**
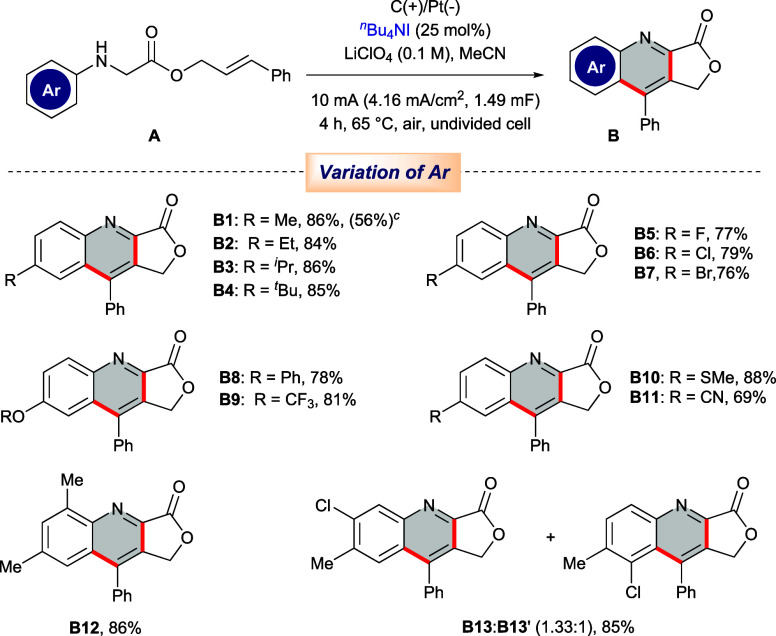
Scope for the Synthesis of Substituted Fused Quinolines^a^^,^^b^

a Reaction conditions: **A** (0.2 mmol), ^*n*^Bu_4_NI (0.05 mmol), LiClO_4_ (0.1 M) in MeCN (5 mL), graphite anode, Pt cathode, undivided cell, constant current = 10 mA, at 65 °C under air for 4h.

b Isolated yield.

c Isolated yield in the gram-scale reaction.

Next, the aryl core positioned in the cinnamyl counterpart was varied (Table 3). Unsubstituted and alkyl-, aryl-, and halogen-substituted benzene rings were converted smoothly to the corresponding fused quinolines (**B14** to **B17**) with good to excellent yields. Next, substitution at the ortho-carbon of the aromatic system was investigated, and corresponding quinolines having -Me (**B18**), -F (**B19**), and -Br (**B20**) groups at the C-2 position were acquired with excellent efficacy. Interestingly, in cases of larger *ortho*-substituents (Me and Br), formations of single diastereomers were observed, whereas *ortho*-F resulted in a mixture of diastereomers (**B19** and **B19’**).

**Table 3:**
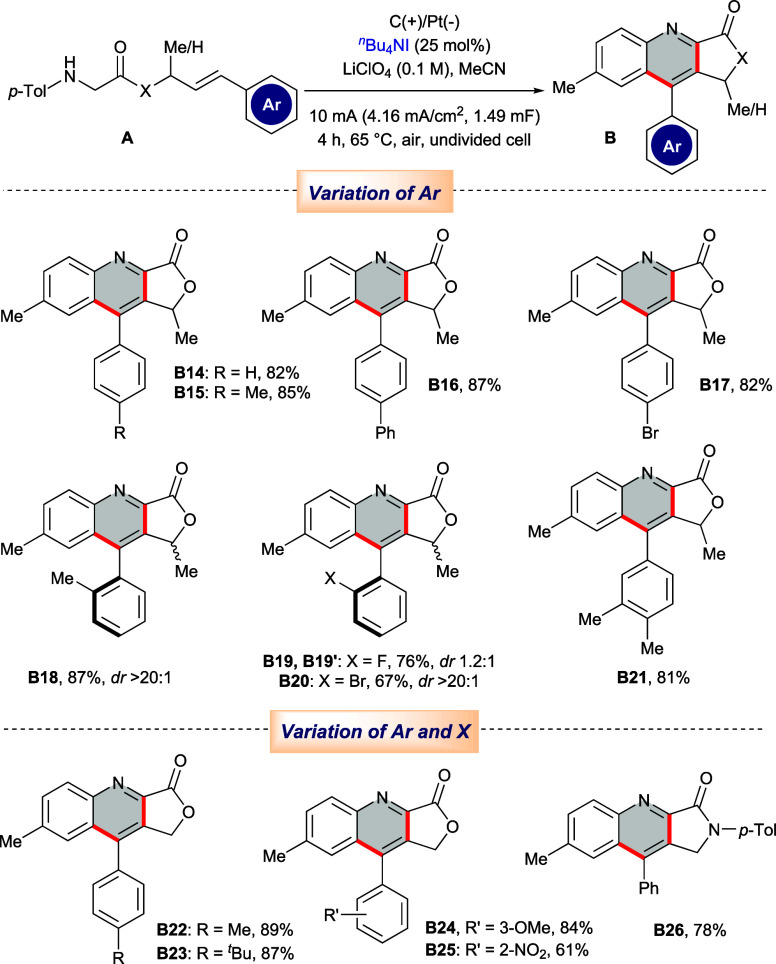
Scope for the Synthesis of Substituted Fused Quinolines^a^^,^^b^

a Reaction conditions: **A** (0.2 mmol), ^*n*^Bu_4_NI (25 mol %), LiClO_4_ (0.1 M) in MeCN (5 mL), graphite anode, Pt cathode, undivided cell, constant current = 10 mA, at 65 °C under air for 4h.

b Isolated yield.

Pleasingly, 3,4-disubstituted derivative **A21** also fabricated corresponding quinoline **B21** with an excellent yield of 81%. Finally, derivatives of cinnamyl alcohol with various alkyl, alkoxy, and -NO_2_ substituents at different positions of the aromatic nucleus were tested under the optimized reaction condition, and corresponding quinolines were manufactured efficiently (**B22** to **B25**). Pleasingly, this electro-redox protocol also allowed the construction of lactam-fused quinoline **B26**, with a *p*-tolyl group on amide nitrogen from its appropriate precursor **A26**, with a decent yield of 78%. However, the synthesis of a few other electronically different lactam-fused quinoline derivatives was unsuccessful, displaying the limitation of the methodology (see Supporting Information for details).

To gain detailed mechanistic insights, cyclic voltammetric (CV) experiments were performed with standard substrate **A1** and mediator ^*n*^Bu_4_NI (Figure 2). CV measurement of ^*n*^Bu_4_NI revealed two consecutive oxidation peaks at 0.71 and 1.00 V, indicating electrochemical generation of reactive iodine species in the reaction environment (Figure 2a). Interestingly, the CV diagram of **A1** also exhibited two distinct oxidation waves at 0.95 and 1.27 V (Figure 2b), which likely corresponds to a sequential two-electron oxidation process. A mixture of both reaction components demonstrated the oxidation of ^*n*^Bu_4_NI prior to the substrate (Figure 2c). Notably, in the mixture, the onset potentials of the first oxidation wave of **A1** and the second oxidation wave of ^*n*^Bu_4_NI merged, resulting in an increased current intensity. This observation supports the possibility of a parallel oxidation of both species.

**Figure 2:**
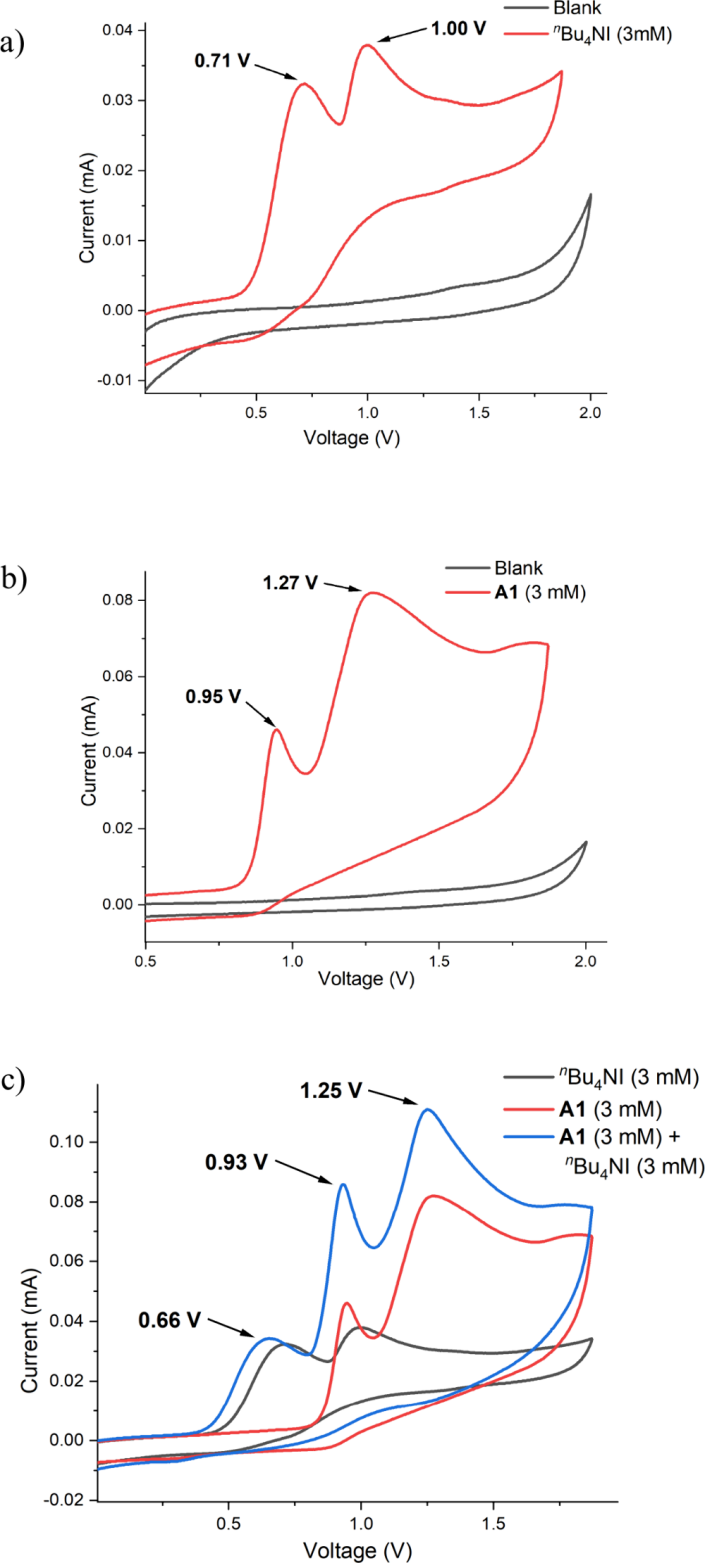
Cyclic voltammograms.

To acquire profound mechanistic knowledge regarding this [4 + 2] cycloaddition protocol, a series of control experiments were performed. Treatment of **A1** with a superstoichiometric amount of molecular iodine or *N*-iodosuccinimide in the presence of electricity produced quinoline **B1** with 72% and 68% yields, respectively. However, the product yields were significantly depleted when similar reactions were conducted without electricity (Scheme 2a). The product was isolated in 84% yield when substrates were placed as anolytes in a divided cell. On the contrary, no product formation was noticed when reactants were electrolyzed in the cathodic chamber (Scheme 2b), discarding any involvement of substrate reduction for the aforementioned transformation.

**Scheme 2:**
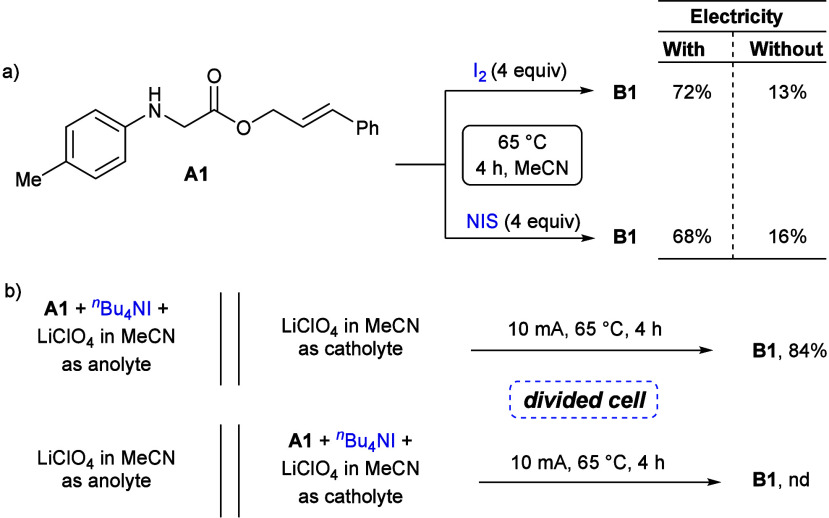
Control Experiments

Based on the evidence accumulated from control experiments and cyclic voltammetric analysis, a plausible mechanism of this [4 + 2] cycloaddition approach is illustrated in Scheme 3. Consecutive oxidations of iodide generate molecular iodine in the reaction environment,^41^ which reacts with substrate **A1** to produce the *N*-iodo species **I**.^42−44^ Elimination of HI from **I** and subsequent protonation fabricates reactive iminium intermediate **II**. Notably, **II** can also be produced following direct two-electron oxidation of **A1** on the anodic surface (through radical cation intermediate **III**). [4 + 2] Cycloaddition and follow-up rearomatization deliver the tetrahydroquinoline **V**. Finally, oxidative aromatization, presumably through iodide electrocatalysis, produces quinoline **B1**. The reduction of protons at the cathode preserves electrical neutrality by releasing dihydrogen.

**Scheme 3:**
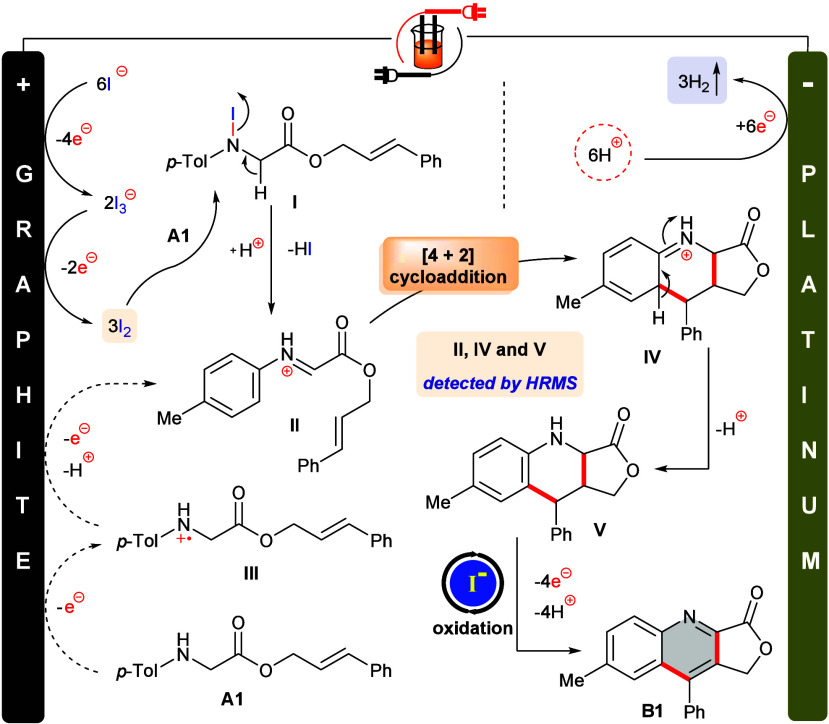
Plausible Reaction Mechanism

In conclusion, we have established a [4 + 2] cycloaddition protocol to synthesize a series of lactone- or lactam-fused quinoline scaffolds employing diversely substituted cinnamyl 2-(phenylamino)-acetates or amides. This mild and atom-economic electro-redox methodology operates in the presence of a catalytic amount of iodide salt, eliminating the requirement for transition metals and chemical oxidants, making it environmentally sustainable and economically viable. Mechanistic studies revealed the oxidation of amine to its corresponding reactive imine intermediate with the help of electrogenerated molecular iodine, resulting in a subsequent [4 + 2] cycloaddition furnishing C–C bonds. Further developments in synthesizing important scaffolds via the electro-oxidation of amines are underway in our laboratory.

## Data Availability

The data underlying this study are available in the published article and its Supporting Information.

## Supporting Information Available

Experimental procedures, ^1^H and ^13^C NMR spectra, characterization data of compounds, cyclic voltammogram, and pictures of the electrochemical setup (PDF)
