# Supplementary material for: Easy Access to Fused Tricyclic Quinoline Derivatives through Metal-Free Electrocatalytic [4 + 2] Annulation
Source: ACS Org Inorg Au. 2024 Aug 8;4(5):492–7. doi: 10.1021/acsorginorgau.4c00037 (PMC11450728; doi:10.1021/acsorginorgau.4c00037)

Supporting Information

Easy Access to Fused Tricyclic Quinoline Derivatives through Metal-Free Electrocatalytic [4 + 2] Annulation

Sayan Ghosh,† Samrat Mallick,† Devika Karolly and Suman De Sarkar*
Department of Chemical Sciences, Indian Institute of Science Education and Research Kolkata, Mohanpur-741246, West Bengal, India

† These authors contributed equally

Email: sds@iiserkol.ac.in

Table of Contents

1. General Information S2
2. Electrochemical Set-up S3
3. General Procedure for the Preparation of Starting Materials S3
4. Optimization Study: S6
5. General Procedure for the Electrochemical [4+2] Cycloaddition (GP) S7
6. Characterization Data for Synthesized Compounds: S8
7. Mechanistic Studies S18
8. Cyclic Voltammetry Experiment S19
9. Failed Substrates S21
10. References S21
11. NMR Spectra for Synthesized Compounds S22
12. HRMS spectra of Intermediate S52

1. General Information:

Electrochemical reactions were performed under air using pre-dried glassware. MeCN and other solvents were purchased from Merck Life Science Private Limited (Emplura grade) and were directly used without further drying or purification. All reagents were obtained from commercial sources and used without further purification. Chemicals were bought from Alfa Aesar, Avra Chemicals and BLD Pharmatech and used without further purification. All the starting materials were synthesized following the reported procedures[1,2]. $LiClO_4$ was purchased from Alfa Aesar and TCI chemicals. AXIOMET AX-3003P power supply was used for electrolysis. Graphite and other electrodes were purchased from IKA. Yields refer to isolated compounds, estimated to be >95% pure as determined by ^{1}H NMR. Nuclear magnetic resonance (NMR) spectroscopy was performed using Bruker 500 MHz and Jeol 400 MHz spectrometers, chemical shifts (δ) are provided in ppm. Thin layer chromatography was performed on Merck pre-coated silica gel 60 F254 aluminum sheets with detection under UV light at 254 nm. Chromatographic separations were carried out on silica gel (100−200 mesh) purchased from Merck Life Science Private Limited. Cyclic voltammetry was measured on Klyte Research Model 263A. HRMS was performed in Waters XEVO G2-XS QTOF mass spectrometers.

2. Electrochemical Set-up:

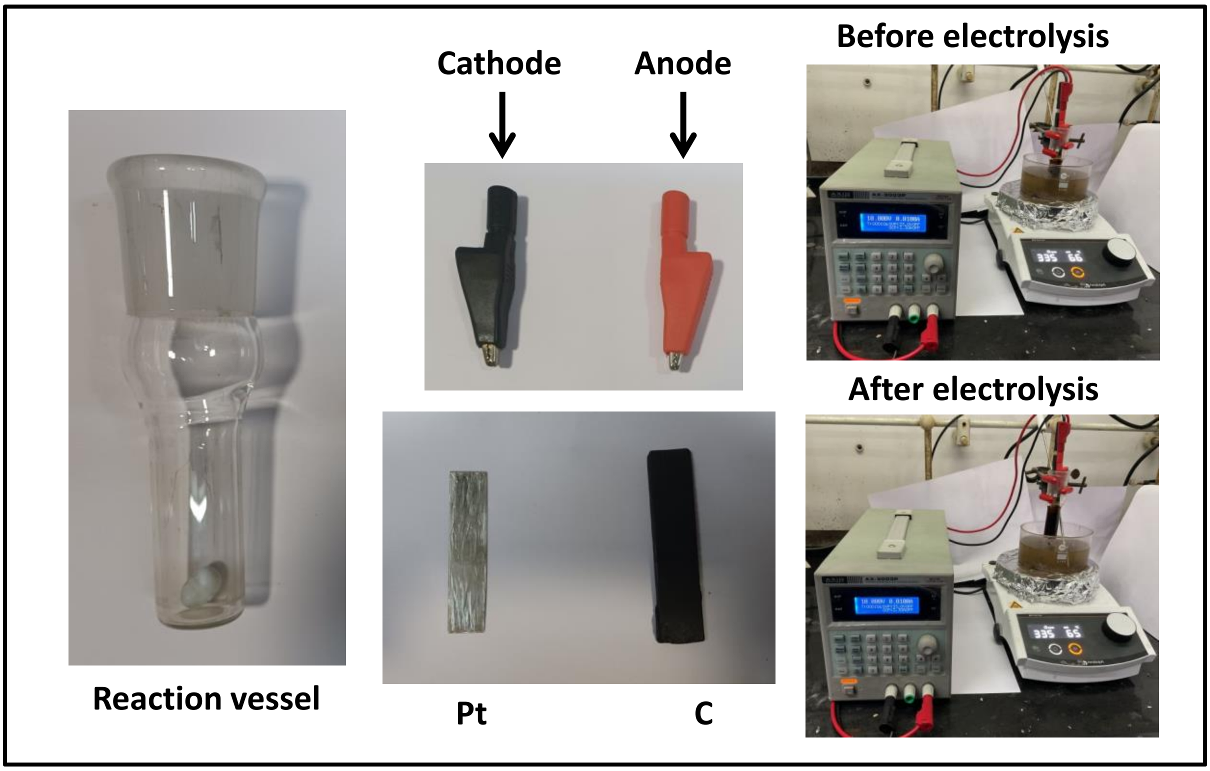

3. General Procedure for the Preparation of Starting Materials:

3.1. Preparation strategy for the starting material with variation in the aniline side:

Step 1: Acetylation of *trans*-cinnamyl alcohol using bromoacetyl bromide[2].

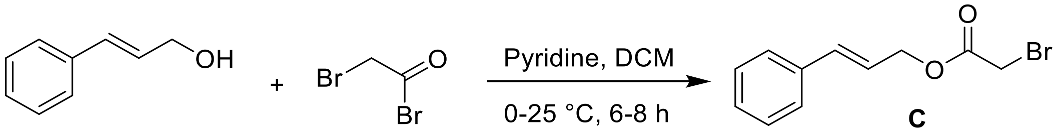

Bromoacetyl bromide (1 equiv, 30.0 mmol) was added dropwise to a solution of *trans*-cinnamyl alcohol (1 equiv, 30 mmol) and pyridine (1 equiv, 30 mmol) in CH_2Cl_2 (60 mL) at 0 °C to form a white suspension. The reaction mixture was stirred for 20 minutes at 0 °C and additional 6-8 hours at 25 °C. The progress of the reaction was monitored by TLC (Thin layered Chromatography). After completion, water was added (50 mL) to the reaction mixture to separate the organic layer. The aqueous layer was extracted with CH_2Cl_2 (20 mL x 2). After drying the resulting mixture over Na_2SO_4, the organic solvent was concentrated to obtain the crude product. Finally, product **C** was purified using column chromatography with 3% ethyl acetate in hexane.

Step 2: Coupling of Aniline derivatives with cinnamyl 2- bromoacetate (C)[2]

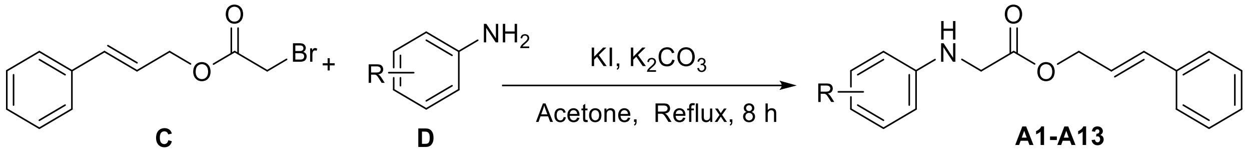

A 250 mL round-bottom flask was charged with cinnamyl 2- bromoacetate (**C**) (1.1 equiv, 55 mmol), K_2CO_3 (1.2 equiv, 60 mmol), KI (1.1 equiv, 55 mmol), anilines (1 equiv, 50 mmol) and acetone (150 mL). The mixture was heated to reflux in an oil bath for 8 h. Reaction progress was monitored using TLC and after completion, acetone was evaporated in vacuo and work-up was performed using DCM and brine. The solvent was removed under reduced pressure, and the crude product was purified by silica gel column chromatography to give **A**.

3.2. Preparation of starting materials with other variations:

a) From chalcones:

Step 1: Preparation of chalcones from Benzaldehyde derivatives and Acetone[3]

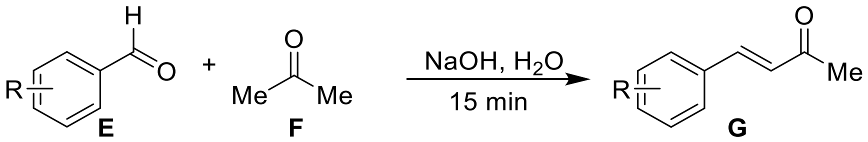

To a mixture of aldehyde (1 equiv, 20 mmol) in acetone (50.0 mL), aqueous NaOH (100 mL, 1 M in water) was added dropwise at 0 °C. The solution was stirred at room temperature. After completion of reaction (monitored using TLC) acetone was removed by evaporation in vacuo. Then, the crude mixture was dissolved in EtOAc and washed with saturated aqueous NaCl solution. The aqueous layer was washed with EtOAc (2 × 25 ml), and the combined organic layers were dried over Na_2SO_4 and concentrated under reduced pressure. Finally, the residue (**G**) was purified by flash chromatography on silica gel.

Step 2: Reduction of Chalcone to Cinnamyl alcohol[4]

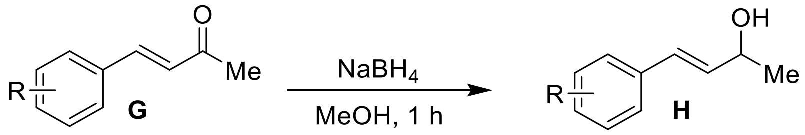

Sodium borohydride (2 equiv,40 mmol) was slowly added to a solution of **G** (1 equiv, 20 mmol) in methanol (50 mL) at 0 °C. The reaction mixture was stirred for 2 hours at 0 °C. The reaction mixture was monitored by TLC. After completion, the reaction mixture was

quenched using saturated aqueous NH_4Cl. After concentrating the resultant mixture under reduced pressure, the residue was extracted with ethyl acetate. The combined organic layers was dried with Na_2SO_4 and concentrated under reduced pressure to obtain **H** which was directly used in the next step without further purification.

Step 3: Acetylation of cinnamyl alcohol (G) with bromoacetyl bromide[2]

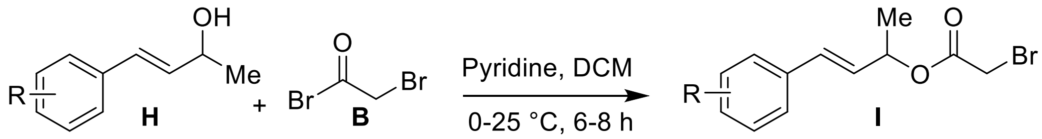

Step 3 was followed according to the aforementioned procedure 2.1.

Step 4: Coupling of aniline derivatives with I[8]

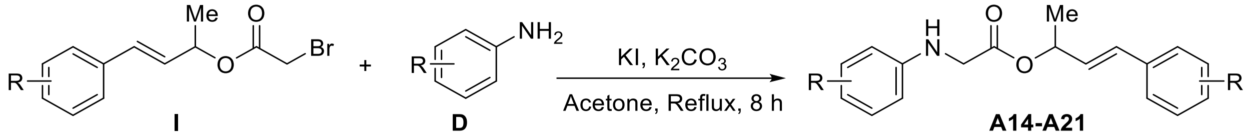

Step 3 was followed according to the aforementioned procedure 2.1.

b) From cinnamyl alcohol derivatives:

Step 1: Wittig reaction[5]

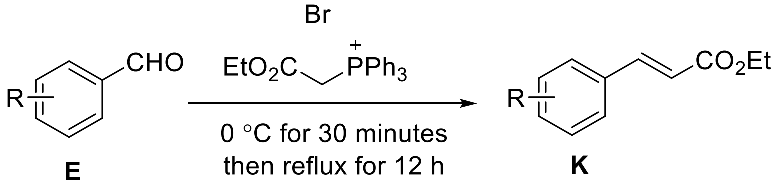

Solution of phosphonoacetate (3 equiv, 30 mmol) in dry THF was added dropwise under argon over 5 minutes to a stirred suspension of sodium hydride (3 equiv, 30 mmol) in dry THF (30 ml). The resulting mixture was stirred at 0 °C for an additional 15 min. A solution of aldehyde (1.0 equiv, 10 mmol) in dry THF was added slowly to the resulting mixture and stirred at 0 °C for an additional 0.5 h. The reaction mixture was refluxed in an oil bath for 12 h and then allowed to cool to ambient temperature. The reaction mixture was quenched with saturated aqueous NH_4Cl and the aqueous layer was extracted with EtOAc (2 X 30 ml). The combined organic layers were washed with brine, dried over Na_2SO_4, and concentrated in vacuo.

Step 3: Reduction of Cinnamyl ester into Cinnamyl alcohol[5]

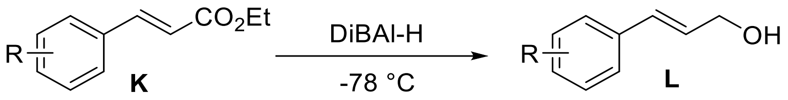

10 mL of DIBAL-H (1M solution in hexane) (2.25 equiv, 9 mmol) was added dropwise into the cooled solution of acrylic ester derivative (1 equiv, 4 mmol) in dry CH_2Cl_2 (20 mL), at -78 °C under argon atmosphere. the mixture was stirred for 1.5 hour at -78 °C. After completion the excess of DIBAL-H was quenched with 10% aqueous NaOH, followed by addition of 2 mL of saturated solution of NH_4Cl. The aqueous layer was extracted with CH_2Cl_2 (3 × 25 mL) and the organic phase was dried over anhydrous Na_2SO_4 and concentrated in vacuo.

Step 4: Acetylation of Cinnamyl alcohol (L) with Bromoacetyl bromide[2]

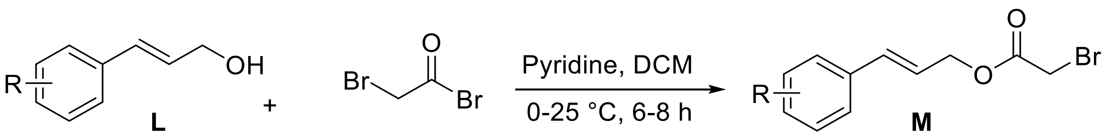

Step 4 was followed according to the aforementioned procedure 2.1.

Step 5: Coupling of Aniline derivatives with M[2]

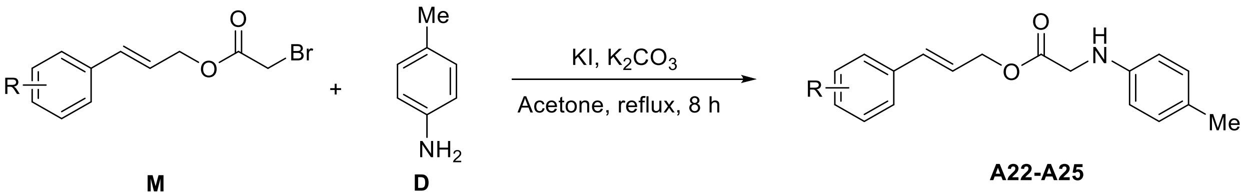

Step 5 was followed according to the aforementioned procedure 2.1.

4. Optimization Study:

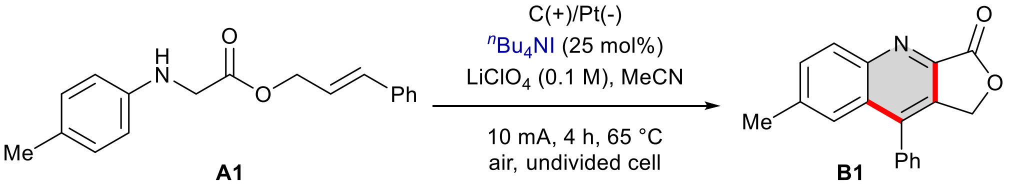

Entry	Deviation from standard conditions	Yield^b (%)
1.	at room temperature	38
**2.**	**none**	**86**

3	at 50 °C	74
4	at 80 °C	58
5.	without ^{n}Bu$_4$NI	40
6	KI/NaI/NH$_4$I instead of TBAI	56/42/81
5	^{n}Bu$_4$NBr instead of ^{n}Bu$_4$NI	49
7.	5/30/50 mol % of ^{n}Bu$_4$NI	62/83/74
8.	^{n}Bu$_4$NPF$_6$/ ^{n}Bu$_4$NOTs as electrolyte	42/19
9.	EtOH/MeOH/DMF/DMSO as solvent	Nd/nd/71/nd
10.	C/Ni foam cathode	23/42
11	Pt/glassy carbon as anode	15/64
12	20 mA for 2 h/5 mA for 8 h	48/57
13	without electricity	nd

In an oven-dried undivided reaction flask (10 mL) equipped with a stir bar, (**A1**) (0.2 mmol, 1.0 equiv), electrolyte (0.1 M) and solvent (5 mL) were added. Then electrodes were dipped into the reaction mixture, and resulting solution was electrolyzed at a constant current of 5-20 mA at RT to 65 ℃ in an oil bath, open to the air for 2-8 h. After electrolysis, the reaction mixture was evaporated under reduced pressure and purification of the product was performed by column chromatography using 12% ethyl acetate in hexane yielded **B1.**

5. General Procedure for the Electrochemical [4+2] Cycloaddition (GP):

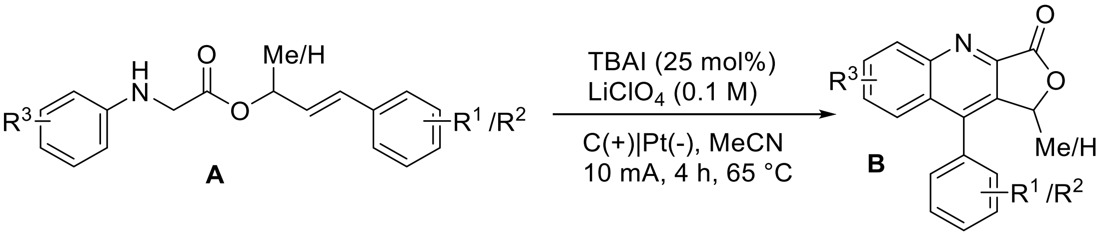

A (0.2 mmol, 1.0 equiv), TBAI (25 mol%) and lithium perchlorate (53 mg, 0.1 M) were taken in an oven dried undivided cell and dissolved in 5 ml of MeCN solvent. Thereafter graphite (3.0 cm×0.8 cm×0.2 cm) and platinum plate (3.0 cm×0.8 cm×0.025 cm) electrodes were dipped into the reaction mixture and connected with an AXIOMET AX-3003P power supply. The electrolysis was conducted along with continuous stirring for 4 h at 10 mA of constant current under air atmosphere at 65 °C in an oil bath. After electrolysis, the reaction mixture was evaporated under reduced pressure and purification of the product by column chromatography using 10% – 30% ethyl acetate in hexane yielded **B.**

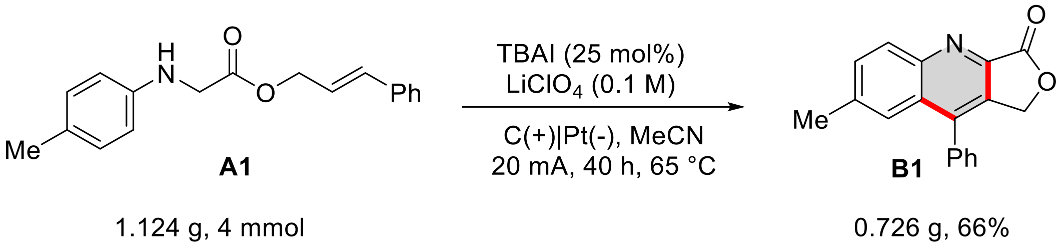

For gram-scale synthesis of B1: Cinnamyl *p*-tolylglycinate (**A1**, 1.124 g, 4.0 mmol, 1.0 equiv), TBAI (0.37 g, 1 mmol, 25 mol%) and were taken in a dry undivided cell and dissolved in 40 mL of acetonitrile solvent. The cell was equipped with a graphite cathode (5.2 cm×3.2 cm×0.2 cm) and a graphite anode (5.2 cm×3.2 cm×0.2 cm). Thereafter the reaction mixture was stirred and electrolyzed at a constant current of 20 mA under air atmosphere and at 65 °C in an oil bath. After completion, purification was done by column chromatography in silica gel using 12% ethyl acetate in hexane yielded (**B1,** 0.726 g, 56%) as a white solid.

6. Characterization Data for Synthesized Compounds:

7-Methyl-9-phenylfuro[3,4-*b*]quinolin-3(1*H*)-one (B1)[1]:

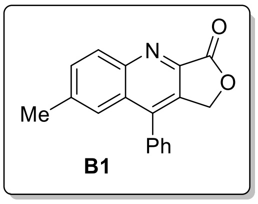

GP was followed using cinnamyl *p*-tolylglycinate (**A1**, 56 mg, 0.2 mmol), TBAI (19 mg, 25 mol%). After 4 h, purification by column chromatography in silica gel (100−200 mesh) using 12% ethyl acetate in hexane yielded (**B1,** 47 mg, 86%) as a white solid. **^{1}H NMR** (400 MHz, $CDCl_3$) δ 8.32 (d, *J* = 8.6 Hz, 1H), 7.71 – 7.56 (m, 5H), 7.44 (dd, *J* = 7.9, 1.9 Hz, 2H), 5.36 (s, 2H), 2.51 (s, 3H). **^{13}C{1H} NMR** (126 MHz, $CDCl_3$) δ 169.0, 149.5, 143.5, 143.0, 140.1, 133.9, 133.2, 132.6, 131.1, 129.5, 129.4, 128.9, 128.1, 124.4, 67.9, 22.2.

7-Ethyl-9-phenylfuro[3,4-*b*]quinolin-3(1*H*)-one (B2):

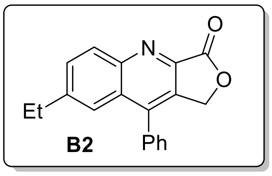

GP was followed using cinnamyl (4-ethylphenyl)glycinate (**A2**, 59 mg, 0.2 mmol), TBAI (19 mg, 25 mol%). After 4 h, purification by column chromatography in silica gel (100−200 mesh) using 12% ethyl acetate in hexane yielded (**B2**, 49 mg, 84%) as a white solid. **^{1}H NMR** (500 MHz, $CDCl_3$) δ 8.34 (d, *J* = 8.7 Hz, 1H), 7.72 (dd, *J* = 8.8, 1.9 Hz, 1H), 7.65 – 7.55 (m, 4H), 7.47 – 7.42 (m, 2H), 5.36 (s, 2H) 2.82 – 2.77 (m, 2H), 1.26 (t, *J* = 7.6 Hz, 3H).

^{13}C{1H} NMR (126 MHz, $CDCl_3$) δ 169.0, 149.7, 146.2, 143.5, 143.1, 133.9, 132.6, 132.1, 131.3, 129.5, 129.4, 128.9, 128.1, 123.2, 67.9, 29.4, 15.3. **HRMS-ESI** (m/z): calcd for $C_{19}H_{16}NO_2^+$ $[M + H]^+$ 290.1176; found 290.1180.

7-Isopropyl-9-phenylfuro[3,4-*b*]quinolin-3(1*H*)-one (B3)[1] :

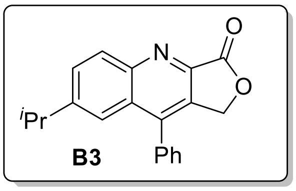

GP was followed using cinnamyl (4-isopropylphenyl)glycinate (**A3**, 62 mg, 0.2 mmol), TBAI (19 mg, 25 mol%). After 4 h, purification by column chromatography in silica gel (100−200 mesh) using 11% ethyl acetate in hexane yielded (**B3**, 52 mg, 86%) as a white solid. **^{1}H NMR** (400 MHz, $CDCl_3$) δ 8.34 (d, *J* = 8.6 Hz, 1H), 7.76 (dd, *J* = 8.6, 2.2 Hz, 1H), 7.69 – 7.54 (m, 4H), 7.45 (dd, *J* = 7.9, 1.8 Hz, 2H), 5.36 (s, 2H), 3.04 (hept, *J* = 7.0 Hz, 1H), 1.27 (d, *J* = 7.2 Hz, 6H). **^{13}C{1H} NMR** (126 MHz, $CDCl_3$) δ 169.0, 150.6, 149.7, 143.4, 143.2, 133.8, 132.5, 131.3, 130.5, 129.5, 129.4, 128.9, 128.0, 121.8, 76.9, 67.9, 34.6, 23.7.

7-(Tert-butyl)-9-phenylfuro[3,4-*b*]quinolin-3(1*H*)-one (B4)[1]:

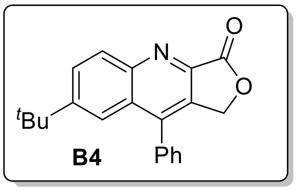

GP was followed using cinnamyl (4-(tert-butyl)phenyl)glycinate (**A4**, 65 mg, 0.2 mmol), TBAI (19 mg, 25 mol%). After 4 h, purification by column chromatography in silica gel (100−200 mesh) using 11% ethyl acetate in hexane yielded (**B4**, 54 mg, 85%) as a white solid. **^{1}H NMR** (500 MHz, $CDCl_3$) δ 8.35 (d, *J* = 9.0 Hz, 1H), 7.94 (dd, *J* = 9.1, 2.1 Hz, 1H), 7.82 (d, *J* = 2.1 Hz, 1H), 7.66 – 7.55 (m, 3H), 7.48 – 7.42 (m, 2H), 5.37 (s, 2H), 1.33 (s, 9H). **^{13}C{1H} NMR** (126 MHz, $CDCl_3$) δ 169.0, 152.7, 149.4, 143.6, 143.5, 133.8, 132.5, 130.9, 129.9, 129.5, 129.4, 129.3, 128.9, 127.7, 120.5, 76.9, 67.9, 35.5, 31.0, 29.8.

7-Fluoro-9-phenylfuro[3,4-*b*]quinolin-3(1*H*)-one (B5)[1] :

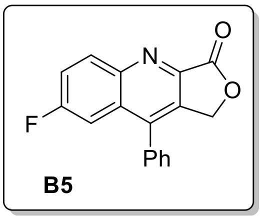

GP was followed using cinnamyl (4-fluorophenyl)glycinate (**A5**, 57 mg, 0.2 mmol), TBAI (19 mg, 25 mol%). After 4 h, purification by column chromatography in silica gel (100−200 mesh) using 12% ethyl acetate in hexane yielded (**B5**, 43 mg, 77%) as a white solid.

^{1}H NMR (500 MHz, $CDCl_3$) δ 8.55 – 8.28 (m, 1H), 7.73 – 7.55 (m, 4H), 7.50 (dd, J = 9.8, 2.8 Hz, 1H), 7.44 (dd, J = 7.9, 1.6 Hz, 2H), 5.40 (s, 2H). **^{13}C{1H} NMR** (126 MHz, $CDCl_3$) δ 168.5, 162.5 (d, $^{1}J_{C\text{-}F}$ = 253.1 Hz), 148.0, 144.1 (d, $^{4}J_{C\text{-}F}$ = 3.1 Hz), 143.5 (d, $^{4}J_{C\text{-}F}$ = 6.4 Hz), 134.2 (d, $^{3}J_{C\text{-}F}$ = 9.5 Hz), 133.2, 133.1, 129.9, 129.7, 129.3 (d, $^{3}J_{C\text{-}F}$ = 10.1 Hz), 128.8, 121.6 (d, $^{2}J_{C\text{-}F}$ = 26.3 Hz), 109.3 (d, $^{2}J_{C\text{-}F}$ = 23.6 Hz), 67.8. **^{19}F NMR{^{1}H}** (471 MHz, $CDCl_3$) δ -107.1.

7-chloro-9-phenylfuro[3,4-*b*]quinolin-3(1*H*)-one (B6)[1]**:**

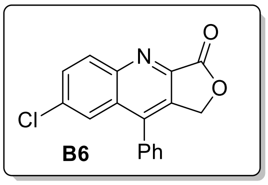

GP was followed using cinnamyl (4-chlorophenyl)glycinate (**A6**, 60 mg, 0.2 mmol), TBAI (19 mg, 25 mol%). After 4 h, purification by column chromatography in silica gel (100−200 mesh) using 12% ethyl acetate in hexane yielded (**A6**, 47 mg, 79%) as a white solid. **^{1}H NMR** (500 MHz, $CDCl_3$) δ 8.35 (d, *J* = 9.0 Hz, 1H), 7.85 (d, *J* = 2.3 Hz, 1H), 7.77 (dd, *J* = 9.0, 2.3 Hz, 1H), 7.66 – 7.58 (m, 3H), 7.46 – 7.42 (m, 2H), 5.39 (s, 2H). **^{13}C{1H} NMR** (126 MHz, $CDCl_3$) δ 168.4, 149.1, 144.7, 143.3, 135.9, 133.2, 133.0, 132.9, 131.9, 130.0, 129.6, 128.8, 128.6, 124.6, 67.8.

7-Bromo-9-phenylfuro[3,4-*b*]quinolin-3(1*H*)-one (B7)[1]**:**

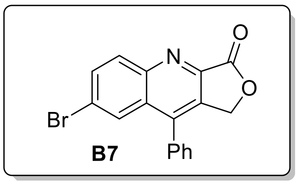

GP was followed using cinnamyl (4-bromophenyl)glycinate (**A7**, 69 mg, 0.2 mmol), TBAI (19 mg, 25 mol%). After 4 h, purification by column chromatography in silica gel (100−200 mesh) using 11% ethyl acetate in hexane yielded (**B7**, 52 mg, 76%) as a white solid. **^{1}H NMR** (500 MHz, $CDCl_3$) δ 8.28 (d, *J* = 9.1 Hz, 1H), 8.03 (d, *J* = 2.2 Hz, 1H), 7.91 (dd, *J* = 9.0, 2.2 Hz, 1H), 7.66 – 7.58 (m, 3H), 7.46 – 7.41 (m, 2H), 5.39 (s, 2H). **^{13}C{1H} NMR** (126 MHz, $CDCl_3$) δ 168.4, 149.3, 144.8, 143.2, 134.5, 133.2, 133.0, 132.9, 130.0, 129.7, 129.0, 128.8, 128.0, 124.4, 67.8.

7-Phenoxy-9-phenylfuro[3,4-*b*]quinolin-3(1*H*)-one (B8)[1] **:**

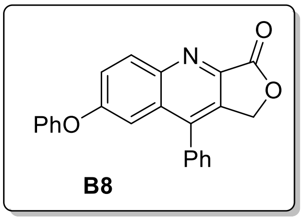

GP was followed using cinnamyl (4-phenoxyphenyl)glycinate (**A8**, 72 mg, 0.2 mmol), TBAI (19 mg, 25 mol%). After 4 h, purification by column chromatography in silica gel (100−200 mesh) using 14% ethyl acetate in hexane yielded (**B8**, 55 mg,

78%) as a white solid. **^{1}H NMR** (500 MHz, $CDCl_3$) δ 8.39 (d, J = 9.4 Hz, 1H), 7.62 – 7.47 (m, 4H), 7.42 – 7.30 (m, 5H), 7.16 (t, J = 7.4 Hz, 1H), 7.08 – 7.01 (m, 2H), 5.38 (s, 2H). **^{13}C{1H} NMR** (126 MHz, $CDCl_3$) δ 168.8, 158.1, 155.8, 147.6, 143.1, 142.6, 133.5, 133.4, 133.0, 130.1, 129.6, 129.4, 128.8, 124.6, 124.1, 119.7, 116.3, 111.1, 67.9.

9-Phenyl-7-(trifluoromethoxy)furo[3,4-*b*]quinolin-3(1*H*)-one (B9) :

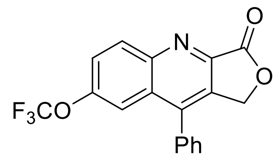

B9

GP was followed using cinnamyl (4-(trifluoromethoxy)phenyl)glycinate (**A9**, 70 mg, 0.2 mmol), TBAI (19 mg, 25 mol%). After 4 h, purification by column chromatography in silica gel (100−200 mesh) using 12% ethyl acetate in hexane yielded (**B9**, 56 mg, 81%) as a white solid. **^{1}H NMR** (500 MHz, $CDCl_3$) 8.45 (d, J = 10.1 Hz, 1H), 7.70 (d, J = 7.0 Hz, 2H), 7.66 – 7.57 (m, 3H), 7.51 – 7.35 (m, 2H), 5.41 (s, 2H). **^{13}C{1H} NMR** (126 MHz, $CDCl_3$) δ 168.3, 149.3 (q, $^3J_{C\text{-}F}$ = 1.9 Hz), 148.8, 145.1, 144.1, 133.8, 133.3, 132.9, 130.1, 129.7, 128.8, 128.5, 124.8, 120.5 (q, $^1J_{C\text{-}F}$ = 259.2 Hz), 115.9, 67.9. **^{19}F NMR{^{1}H}** (471 MHz, $CDCl_3$) δ -57.7. **HRMS-ESI** (m/z): calcd for $C_{18}H_{11}F_3NO_3^+$ $[M + H]^+$ 346.0686; found 346.0679.

7-(Methylthio)-9-phenylfuro[3,4-*b*]quinolin-3(1*H*)-one (B10) :

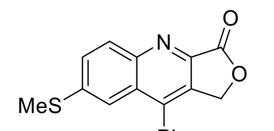

B10

GP was followed using cinnamyl (4-(methylthio)phenyl)glycinate (**A10**, 63 mg, 0.2 mmol), TBAI (19 mg, 25 mol%). After 4 h, purification by column chromatography in silica gel (100−200 mesh) using 13% ethyl acetate in hexane yielded (**B10**, 54 mg, 88%) as a white solid. **^{1}H NMR** (500 MHz, $CDCl_3$) δ 8.27 (dd, J = 9.0, 2.6 Hz, 1H), 7.72 – 7.51 (m, 5H), 7.45 (dd, J = 6.5, 1.7 Hz, 2H), 5.36 (s, 2H), 2.45 (s, 3H). **^{13}C{1H} NMR** (126 MHz, $CDCl_3$) δ 168.8, 149.0, 143.1, 142.1, 141.9, 133.6, 133.3, 131.4, 129.8, 129.7, 129.5, 128.8, 128.4, 119.3, 67.8, 15.2. **HRMS-ESI** (m/z): calcd for $C_{18}H_{14}NO_2S^+$ $[M + H]^+$ 308.0740; found 308.0737.

3-Oxo-9-phenyl-1,3-dihydrofuro[3,4-*b*]quinoline-7-carbonitrile (B11)[1] :

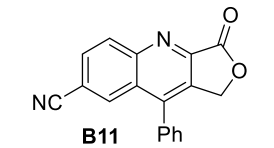

B11

GP was followed using cinnamyl (4-cyanophenyl)glycinate (**A11**, 58 mg, 0.2 mmol), TBAI (19 mg, 25 mol%). After 4 h, purification by column chromatography in silica gel (100−200 mesh) using 14% ethyl acetate in hexane yielded (**B11**, 40 mg, 69%) as a white

solid. **^{1}H NMR** (500 MHz, $CDCl_3$) δ 8.54 (dd, *J* = 8.8, 0.7 Hz, 1H), 8.31 (dd, *J* = 1.8, 0.6 Hz, 1H), 7.99 (dd, *J* = 8.8, 1.8 Hz, 1H), 7.71 – 7.61 (m, 3H), 7.49 – 7.38 (m, 2H), 5.45 (s, 2H). **^{13}C{1H} NMR** (126 MHz, $CDCl_3$) δ 167.8, 151.5, 147.3, 145.3, 133.7, 133.1, 132.6, 132.2, 131.2, 130.6, 130.0, 128.9, 127.4, 118.2, 113.3, 67.9.

5,7-Dimethyl-9-phenylfuro[3,4-*b*]quinolin-3(1*H*)-one (B12)[2] :

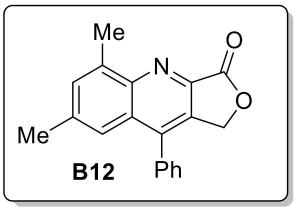

GP was followed using cinnamyl (2,4-dimethylphenyl)glycinate (**A12**, 59 mg, 0.2 mmol), TBAI (19 mg, 25 mol%). After 4 h, purification by column chromatography in silica gel (100−200 mesh) using 11% ethyl acetate in hexane yielded (**B12**, 50 mg, 86%) as a white solid. **^{1}H NMR** (500 MHz, $CDCl_3$) δ 7.63 – 7.54 (m, 3H), 7.52 (s, 1H), 7.46 – 7.39 (m, 3H), 5.32 (s, 2H), 2.90 (s, 3H), 2.45 (s, 3H). **^{13}C{1H} NMR** (126 MHz, $CDCl_3$) δ 169.3, 148.8, 142.9, 142.2, 139.6, 139.2, 134.3, 133.2, 132.5, 129.3, 129.3, 129.0, 128.2, 122.3, 67.7, 22.2, 18.6.

6-Chloro-7-methyl-9-phenylfuro[3,4-*b*]quinolin-3(1*H*)-one (B13) and 8-chloro-7-methyl-9-phenylfuro[3,4-*b*]quinolin-3(1*H*)-one (B13’) :

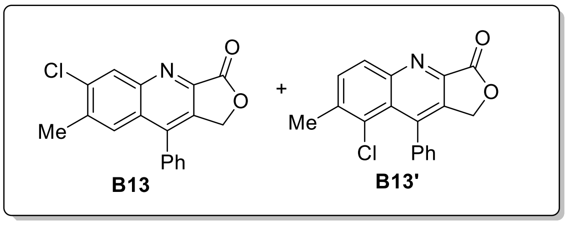

GP was followed using cinnamyl (2-chloro-4-methylphenyl)glycinate (**A13**, 63 mg, 0.2 mmol), TBAI (19 mg, 25 mol%). After 4 h, purification by column chromatography in silica gel (100−200 mesh) using 12% ethyl acetate in hexane yielded as an inseparable mixture in the ratio of 4:3 (**B13 + B13’**, 53 mg, 85%) as a white solid. **^{1}H NMR** (500 MHz, $CDCl_3$) δ 8.40 (s, 1H), 8.29 (d, *J* = 8.7 Hz, 0.72H), 7.74 (d, *J* = 8.7 Hz, 0.77H), 7.71 (s, 1H), 7.66 – 7.57 (m, 3.19H), 7.52 – 7.47 (m, 2.36H), 7.46 – 7.41 (m, 1H), 7.31 – 7.29 (m, 1H), 7.30-7.28 (m, 1.54H), 5.36 (s, 2H), 5.16 (s, 1.50H), 2.55 (s, 2.30H), 2.52 (s, 3H). **^{13}C{1H} NMR** (126 MHz, $CDCl_3$) δ 168.6, 168.5, 151.2, 149.9, 144.5, 143.7, 143.6, 143.3, 139.9, 138.7, 138.7, 137.2, 135.7, 133.6, 133.4, 132.7, 130.5, 130.3, 130.0, 129.8, 129.6, 128.9, 128.8, 128.7, 127.9, 126.7, 126.3, 125.6, 68.2, 67.9, 22.0, 21.1. HRMS-ESI (m/z): calcd for $C_{18}H_{12}ClNO_2^+$ $[M + H]^+$ 310.0629; found 310.0620.

1,7-Dimethyl-9-phenylfuro[3,4-*b*]quinolin-3(1*H*)-one (B14)[2]:

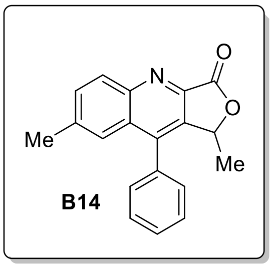

GP was followed using (*E*)-4-phenylbut-3-en-2-yl *p*-tolylglycinate (**A14**, 59 mg, 0.2 mmol), TBAI (19 mg, 25 mol%). After 4 h, purification by column chromatography in silica gel (100−200 mesh) using 13% ethyl acetate in hexane yielded (**B14**, 47 mg, 82%) as a white solid. **^{1}H NMR** (500 MHz, $CDCl_3$) δ 8.28 (d, *J* = 8.7 Hz, 1H), 7.66 – 7.50 (m, 5H), 7.42 – 7.36 (m, 2H), 5.77 (d, *J* = 6.6 Hz, 1H), 2.48 (s, 3H), 1.20 (d, *J* = 6.6 Hz, 3H) . **^{13}C{1H} NMR** (126 MHz, $CDCl_3$) δ 168.3, 149.1, 143.6, 143.0, 139.9, 136.8, 133.9, 133.1, 130.9, 129.7, 129.6, 129.3, 128.9, 128.3, 128.2, 124.5, 76.6, 22.1, 19.3.

1,7-Dimethyl-9-(*p*-tolyl)furo[3,4-*b*]quinolin-3(1*H*)-one (B15):

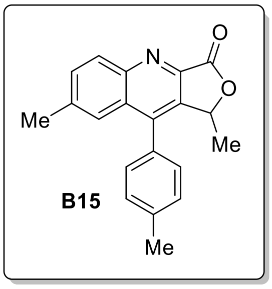

GP was followed using (*E*)-4-(*p*-tolyl)but-3-en-2-yl *p*-tolylglycinate (**A15**, 62 mg, 0.2 mmol), TBAI (19 mg, 25 mol%). After 4 h, purification by column chromatography in silica gel (100−200 mesh) using 12% ethyl acetate in hexane yielded (**B15**, 52 mg, 85%) as a white solid. **^{1}H NMR** (500 MHz, $CDCl_3$) δ 8.31 (d, *J* = 8.7 Hz, 1H), 7.66 (d, *J* = 8.7 Hz, 1H), 7.57 (s, 1H), 7.46 – 7.28 (m, 3H), 5.78 (q, *J* = 6.7 Hz, 1H), 2.50 (d, *J* = 7.9 Hz, 6H), 1.23 (d, *J* = 8.4 Hz, 3H). **^{13}C{1H} NMR** (126 MHz, $CDCl_3$) δ 168.5, 149.3, 143.7, 143.3, 139.8, 139.4, 136.9, 133.1, 131.1, 130.9, 130.3, 129.7, 128.6, 128.2, 124.6, 76.7, 22.2, 21.5, 19.4. **HRMS-ESI** (m/z): calcd for $C_{20}H_{18}NO_2^+$ [M + H]$^+$ 304.1332; found 304.1338.

9-([1,1'-biphenyl]-4-yl)-1,7-dimethylfuro[3,4-*b*]quinolin-3(1*H*)-one (B16):

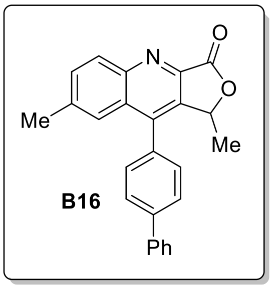

GP was followed using (*E*)-4-(*p*-tolyl)but-3(*E*)-4-(4-fluorophenyl)but-3-en-2-yl *p*-tolylglycinate (**A16**, 74 mg, 0.2 mmol), TBAI (19 mg, 25 mol%). After 4 h, purification by column chromatography in silica gel (100−200 mesh) using 12% ethyl acetate in hexane yielded (**B16**, 64 mg, 87%) as a white solid. **^{1}H NMR** (500 MHz, $CDCl_3$) δ 8.33 (d, *J* = 8.6 Hz, 1H), 7.87 – 7.81 (m, 2H), 7.75 – 7.70 (m, 2H), 7.68 (dd, *J* = 8.8, 1.9 Hz, 1H), 7.64 – 7.60 (m, 1H), 7.54 – 7.48 (m, 3H), 7.48 – 7.41 (m, 2H), 5.84 (q, *J* = 6.6 Hz, 1H), 2.52 (s, 3H), 1.28 (d, *J* = 6.6 Hz, 3H). **^{13}C{1H} NMR** (126 MHz, $CDCl_3$) δ 168.4, 149.3, 143.8, 142.8, 142.2, 140.1, 140.0, 137.0, 133.3, 132.8,

131.2, 130.3, 129.2, 128.9, 128.5, 128.3, 128.2, 127.6, 127.3, 124.6, 76.7, 22.3, 19.6. **HRMS-ESI (m/z):** calcd for $C_{25}H_{20}NO_2^+$ $[M + H]^+$ 366.1489; found 366.1488.

9-(4-Bromophenyl)-1,7-dimethylfuro[3,4-*b*]quinolin-3(1*H*)-one (B17):

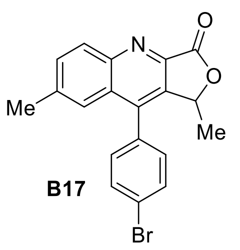

GP was followed using (*E*)-4-(4-bromophenyl)but-3-en-2-yl *p*-tolylglycinate (**A17**, 75 mg, 0.2 mmol), TBAI (19 mg, 25 mol%). After 4 h, purification by column chromatography in silica gel (100−200 mesh) using 12% ethyl acetate in hexane yielded (**B17**, 60 mg, 82%) as a white solid. **^{1}H NMR** (400 MHz, CDCl3*D*) δ 8.24 (dd, *J* = 8.7, 1.7 Hz, 1H), 7.82 – 7.69 (m, 2H), 7.69 – 7.55 (m, 1H), 7.46 (dt, *J* = 2.0, 0.9 Hz, 1H), 7.30 (dt, *J* = 7.6, 0.8 Hz, 2H), 5.74 (q, *J* = 6.6 Hz, 1H), 2.48 (d, *J* = 1.0 Hz, 3H), 1.23 (dd, *J* = 6.5, 0.9 Hz, 3H). **^{13}C{1H} NMR** (126 MHz, $CDCl_3$) 168.1, 149.1, 143.6, 141.7, 140.3, 136.7, 133.3, 133.0, 132.8, 132.3, 131.4, 131.1, 130.0, 128.1, 124.2, 123.8, 76.4, 22.2, 19.5. **HRMS-ESI** (m/z): calcd for $C_{19}H_{15}BrNO_2^+$ $[M + H]^+$ 368.0281; found 368.0263.

1,7-Dimethyl-9-(o-tolyl)furo[3,4-*b*]quinolin-3(1*H*)-one (B18):

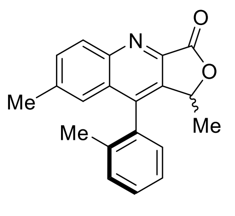

B18 (*dr* >20:1)

GP was followed using (*E*)-4-(*o*-tolyl)but-3-en-2-yl *p*-tolylglycinate (**A18**, 62 mg, 0.2 mmol), TBAI (19 mg, 25 mol%). After 4 h, purification by column chromatography in silica gel (100−200 mesh) using 12% ethyl acetate in hexane yielded (**B18**, 53 mg, 87%) as a white solid. **^{1}H NMR** (500 MHz, $CDCl_3$) δ 8.32 (d, *J* = 8.8 Hz, 1H), 7.67 (dd, *J* = 8.7, 2.0 Hz, 1H), 7.51 – 7.46 (m, 1H), 7.45 – 7.38 (m, 2H), 7.17 (dd, *J* = 7.5, 1.4 Hz, 1H), 5.64 (q, *J* = 6.6 Hz, 1H), 2.48 (s, 3H), 1.97 (s, 3H), 1.23 (d, *J* = 6.6 Hz, 3H). **^{13}C{1H} NMR** (126 MHz, $CDCl_3$) δ 168.4, 148.9, 144.1, 143.1, 140.3, 136.9, 136.2, 133.6, 133.3, 131.3, 130.8, 129.6, 128.4, 128.3, 127.0, 126.1, 124.3, 76.5, 22.2, 20.1, 18.6. **HRMS-ESI** (m/z): calcd for $C_{20}H_{18}NO_2^+$ $[M + H]^+$ 304.1332; found 304.1338.

9-(2-fluorophenyl)-1,7-dimethylfuro[3,4-*b*]quinolin-3(1*H*)-one (B19 and B19'):

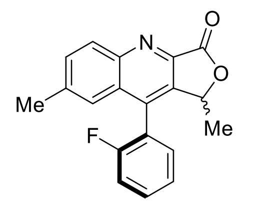

B19 and **B19'** (dr = 1.2:1)

GP was followed using (*E*)-4-(3,4-dimethylphenyl)but-3-en-2-yl *p*-tolylglycinate (**A19**, 63 mg, 0.2 mmol), TBAI (19 mg, 25 mol%). After 4 h, purification by column chromatography in silica gel (100−200 mesh) using 12% ethyl acetate in hexane yielded (**B19** and **B19'**, 47 mg, 76%) as an inseparable mixture of diastereomers (dr 1.2:1) as a white solid. **^{1}H NMR** (400 MHz,

CDCl3) δ 8.39 – 8.15 (m, 1H), 7.68 – 7.62 (m, 1H), 7.62 – 7.55 (m, 1H), 7.50 (s, 1H from major the diastereomer), 7.43 – 7.36 (m, 1H + 1H from the minor diastereomer), 7.36 – 7.28 (m, 2H), 5.76 – 5.64 (m, 1H), 2.48 (s, 3H), 1.24 – 1.20 (m, 3H). **^{13}C{1H} NMR** (101 MHz, CDCl3) For major diastereomer: δ 168.2, 159.5 (d, *J* = 247.8 Hz), 149.2, 143.4, 140.4, 137.5, 136.4, 133.3, 131.9 (d, *J* = 2.7 Hz), 131.8 (d, *J* = 8.1 Hz), 131.1, 128.4, 124.7 (d, *J* = 3.5 Hz), 124.1, 121.1 (d, *J* = 16.5 Hz), 116.8 (d, *J* = 21.4 Hz), 76.3, 22.2, 19.4. **^{13}C{1H} NMR** (101 MHz, CDCl3) For minor diastereomer δ 168.2, 159.4 (d, *J* = 248.5 Hz), 148.8, 143.7, 140.4, 137.8, 137.3, 133.4, 131.9 (d, *J* = 7.9 Hz), 131.0, 130.2 (d, *J* = 2.9 Hz), 128.4 (d, *J* = 0.9 Hz), 125.4 (d, *J* = 3.7 Hz), 124.3 (d, *J* = 1.1 Hz), 121.6 (d, *J* = 16.8 Hz), 116.4 (d, *J* = 21.1 Hz), 76.7, 22.2, 19.0. **^{19}F NMR{^{1}H}** (376 MHz, CDCl3) δ -111.8 (minor diastereomer), -114.0 (major diastereomer). **HRMS-ESI** (m/z): calcd for $C_{19}H_{15}FNO_2^+$ [M + H]$^+$ 308.1081; found 308.1076.

9-(2-Bromophenyl)-1,7-dimethylfuro[3,4-*b*]quinolin-3(1*H*)-one (B20):

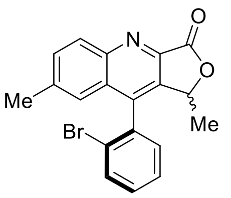

B20 (*dr* >20:1)

GP was followed using (*E*)-4-(2-bromophenyl)but-3-en-2-yl *p*-tolylglycinate (**A20**, 75 mg, 0.2 mmol), TBAI (19 mg, 25 mol%). After 4 h, purification by column chromatography in silica gel (100−200 mesh) using 12% ethyl acetate in hexane yielded (**B20**, 49 mg, 67%) as a white solid. ^{1}H NMR (500 MHz, CDCl3) δ 8.32 (d, *J* = 8.7 Hz, 1H), 7.83 (dd, *J* = 8.1, 1.2 Hz, 1H), 7.67 (dd, *J* = 8.7, 2.0 Hz, 1H), 7.56 (td, *J* = 7.5, 1.2 Hz, 1H), 7.47 (td, *J* = 7.8, 1.7 Hz, 1H), 7.30 (dd, *J* = 7.5, 1.7 Hz, 1H), 7.25 (dt, *J* = 1.8, 0.8 Hz, 1H), 5.61 (q, *J* = 6.7 Hz, 1H), 2.49 (d, *J* = 1.0 Hz, 3H), 1.34 (d, *J* = 6.7 Hz, 3H). **^{13}C{1H} NMR** (126 MHz, CDCl3) δ 168.2, 149.0, 144.1, 141.9, 140.4, 136.6, 135.3, 133.6, 133.4, 131.2, 131.1, 130.2, 128.5, 128.0, 124.4, 123.5, 76.4, 22.3, 18.9. **HRMS-ESI** (m/z): calcd for $C_{19}H_{15}BrNO_2^+$ [M + H]$^+$ 368.0281; found 368.0270.

9-(3,4-Dimethylphenyl)-1,7-dimethylfuro[3,4-*b*]quinolin-3(1*H*)-one (B21):

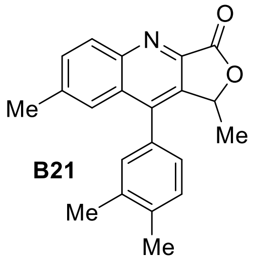

B21

GP was followed using (*E*)-4-(3,4-dimethylphenyl)but-3-en-2-yl *p*-tolylglycinate (**A21**, 65 mg, 0.2 mmol), TBAI (19 mg, 25 mol%). After 4 h, purification by column chromatography in silica gel (100−200 mesh) using 12% ethyl acetate in hexane yielded (**B21**, 51 mg, 81%) mixture of rotamers as a white solid. ^{1}H NMR (400 MHz, CDCl3) δ 8.30 (d, *J* = 8.6 Hz, 1H), 7.71 – 7.62 (m, 1H), 7.58 (d, *J* = 6.5 Hz, 1H), 7.38 – 7.32 (m, 1H), 7.20 – 7.02 (m, 2H), 5.86 – 5.68 (m, 1H), 2.50 and 2.49 (s,

3H), 2.41 (s, 4H), 2.38 and 2.37 (s, 3H), 1.27 – 1.21 (m, 3H). **^{13}C{1H} NMR** (126 MHz, $CDCl_3$) δ 168.5, 149.2, 143.6, 143.5, 139.7, 138.0, 138.0, 137.4, 136.9, 136.9, 133.1, 131.3, 131.0, 130.8, 130.7, 130.1, 129.2, 128.7, 128.6, 127.2, 125.7, 124.7, 76.7, 22.2, 22.2, 20.1, 20.0, 19.8, 19.5, 19.5. **HRMS-ESI** (m/z): calcd for $C_{21}H_{20}NO_2^+$ $[M + H]^+$ 318.1489; found 318.1480.

7-methyl-9-(*p*-tolyl)furo[3,4-*b*]quinolin-3(1*H*)-one (B22):

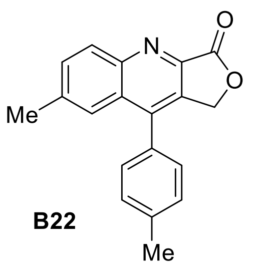

B22

GP was followed using (*E*)-3-(*p*-tolyl)allyl *p*-tolylglycinate (**A22**, 59 mg, 0.2 mmol), TBAI (19 mg, 25 mol%). After 4 h, purification by column chromatography in silica gel (100−200 mesh) using 12% ethyl acetate in hexane yielded (**B22**, 52 mg, 89%) as a white solid. **^{1}H NMR** (500 MHz, $CDCl_3$) δ 8.24 – 8.17 (m, 1H), 7.68 – 7.52 (m, 2H), 7.34 (d, J = 7.6 Hz, 2H), 7.25 (d, J = 7.6 Hz, 2H), 5.27 (s, 2H), 2.43 (s, 3H), 2.42 (s, 3H). **^{13}C{1H} NMR** (126 MHz, $CDCl_3$) δ 169.0, 149.4, 143.4, 143.1, 139.9, 139.6, 133.1, 132.6, 131.0, 130.8, 130.1, 128.8, 128.1, 124.5, 68.0, 22.2, 21.5. **HRMS-ESI** (m/z): calcd for $C_{19}H_{16}NO_2^+$ $[M + H]^+$ 290.1176; found 290.1168.

9-(4-(*tert*-butyl)phenyl)-7-methylfuro[3,4-*b*]quinolin-3(1*H*)-one(B23):

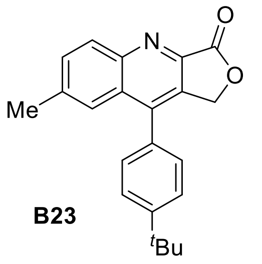

B23

GP was followed using (*E*)-3-(4-(*tert*-butyl)phenyl)allyl *p*-tolylglycinate (**A23**, 67 mg, 0.2 mmol), TBAI (19 mg, 25 mol%). After 4 h, purification by column chromatography in silica gel (100−200 mesh) using 13% ethyl acetate in hexane yielded (**B23**, 58 mg, 87%) as a white solid. **^{1}H NMR** (500 MHz, $CDCl_3$) δ 8.27 (d, J = 8.7 Hz, 1H), 7.71 – 7.66 (m, 1H), 7.67 – 7.56 (m, 3H), 7.39 – 7.35 (m, 2H), 5.36 (s, 2H), 2.51 (s, 3H), 1.43 (s, 9H). **^{13}C{1H} NMR** (126 MHz, $CDCl_3$) δ 169.1, 152.7, 149.4, 143.3, 143.1, 139.9, 133.1, 132.7, 131.0, 130.7, 128.7, 128.1, 126.3, 124.5, 68.1, 35.0, 31.4, 22.2. **HRMS-ESI** (m/z): calcd for $C_{22}H_{22}NO_2^+$ $[M + H]^+$ 332.1645; found 332.1637.

9-(3-methoxyphenyl)-7-methylfuro[3,4-*b*]quinolin-3(1*H*)-one (B24):

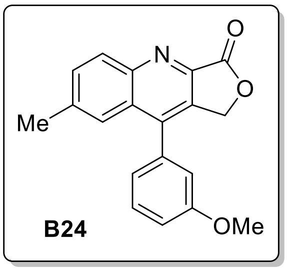

GP was followed using (*E*)-3-(4-methoxyphenyl)allyl *p*-tolylglycinate (**A24**, 62 mg, 0.2 mmol), TBAI (19 mg, 25 mol%). After 4 h, purification by column chromatography in silica gel (100−200 mesh) using 12% ethyl acetate in hexane yielded (**B24**, 51 mg, 84%) as a white solid. **^{1}H NMR** (500 MHz, $CDCl_3$) δ 8.29 (d, *J* = 8.7 Hz, 1H), 7.71 – 7.61 (m, 2H), 7.58 – 7.47 (m, 1H), 7.10 (d, *J* = 8.4 Hz, 2H), 7.06 – 6.90 (m, 1H), 5.35 (s, 2H), 3.88 (d, *J* = 2.1 Hz, 3H), 2.51 (s, 3H). **^{13}C{1H} NMR** (126 MHz, $CDCl_3$) δ 168.9, 160.2, 149.4, 143.4, 142.8, 140.1, 135.1, 133.2, 132.5, 131.0, 130.6, 128.0, 124.4, 121.1, 114.8, 114.6, 67.9, 55.6, 22.2. **HRMS-ESI** (m/z): calcd for $C_{19}H_{16}NO_3^+$ [M + H]$^+$ 306.1125; found 306.1120.

7-methyl-9-(2-nitrophenyl)furo[3,4-b]quinolin-3(1*H*)-one (B25):

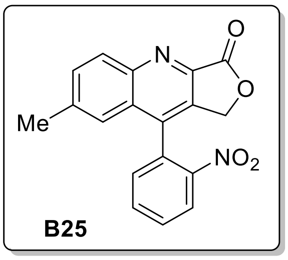

GP was followed using (E)-3-(2-nitrophenyl)allyl p-tolylglycinate (**A25**, 65 mg, 0.2 mmol), TBAI (19 mg, 25 mol%). After 4 h, purification by column chromatography in silica gel (100−200 mesh) using 24% ethyl acetate in hexane yielded (**B25**, 39 mg, 61%) as a pale yellow semi-solid. **^{1}H NMR** (500 MHz, CDCl3) δ 8.36 – 8.31 (m, 2H), 7.88 (td, *J* = 7.5, 1.3 Hz, 1H), 7.85 – 7.78 (m, 1H), 7.68 (dd, *J* = 8.8, 1.9 Hz, 1H), 7.44 (dd, *J* = 7.5, 1.5 Hz, 1H), 7.13 (s, 1H), 5.24 (d, *J* = 4.4 Hz, 2H), 2.46 (s, 3H).. **^{13}C{1H} NMR** (126 MHz, $CDCl_3$) δ 168.5, 149.1, 148.4, 143.5, 141.0, 139.4, 134.4, 133.6, 132.7, 131.6, 131.5, 131.0, 129.1, 127.7, 125.7, 123.2, 67.4, 22.3. **HRMS-ESI** (m/z): calcd for $C_{18}H_{13}N_2O_4^+$ [M + H]$^+$ 321.0870; found 321.0877.

7-methyl-9-phenyl-2-(*p*-tolyl)-1,2-dihydro-3H-pyrrolo[3,4-*b*]quinolin-3-one (B26):

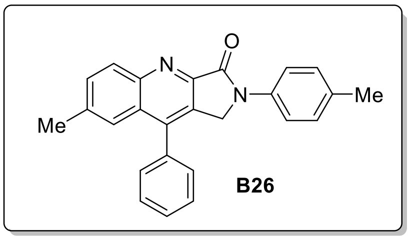

GP was followed using *N*-cinnamyl-*N*-(*p*-tolyl)-2-(*p*-tolylamino)acetamide (**A26**, 74 mg, 0.2 mmol), (19 mg, 25 mol%). After 4 h, purification by column chromatography in silica gel (100−200 mesh) using 30% ethyl acetate in hexane yielded (**B26**, 57 mg, 78%) as a pale yellow semi-solid. **^{1}H NMR** (400 MHz, $CDCl_3$) δ 8.34 (d, *J* = 8.6 Hz, 1H), 7.86 – 7.70 (m, 2H), 7.65 –

7.58 (m, 4H), 7.54 (d, J = 1.8 Hz, 1H), 7.49 – 7.46 (m, 2H), 7.22 – 7.17 (m, 2H), 4.78 (s, 2H), 2.48 (s, 3H), 2.33 (s, 3H). **^{13}C{1H} NMR** (126 MHz, $CDCl_3$) δ 165.5, 138.7, 136.8, 135.2, 134.7, 132.5, 130.9, 129.9, 129.3, 129.2, 129.2, 128.0, 127.8, 124.5, 119.8, 48.4, 22.1, 21.0. **HRMS-ESI** (m/z): calcd for $C_{25}H_{21}N_2O^+$ $[M + H]^+$ 365.1648; found 365.1643.

7. Mechanistic Studies:

a) Without electricity:

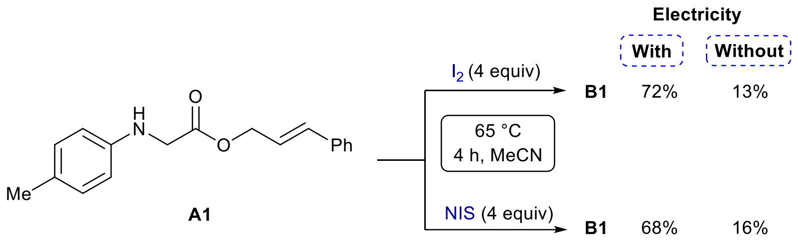

To figure out the role of TBAI in the reaction mechanism, we conducted two experiments with 1a one using I_2 and another one using NIS(N-iodosuccinimide) instead of TBAI with or without electricity by keeping all other conditions the same as the optimized one. In both cases, the product yield was detected.

b) Divided cell experiment:

Electrolysis at anode: cinnamyl *p*-tolylglycinate (**1a**, 56 mg, 0.2 mmol), TBAI (19 mg, 25 mol%). and lithium perchlorate (0.3 M) were taken in the anodic chamber of an oven dry divided cell (separated by G3 sintered) and dissolved in 3 mL of MeCN. The cathode chamber was charged with lithium perchlorate (0.3 M) and 3 mL of MeCN. The cell was equipped with graphite (5.2 cm × 0.8 cm × 0.2 cm) electrodes. The reaction mixture was continuously stirred and electrolyzed with an AXIOMET AX-3003P power at a constant current of 10 mA under air at 65 °C in an oil bath for 1 h. The progress of the reaction was monitored by TLC and After electrolysis, the solvent was evaporated under reduced pressure and purification of the product. was performed by column chromatography using 12% ethyl acetate in hexane with 84% isolated yield of **C1**.

b)

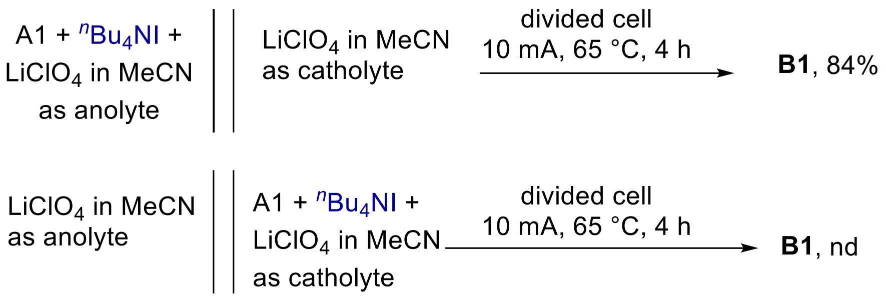

Electrolysis at cathode: cinnamyl *p*-tolylglycinate (**1a**, 56 mg, 0.2 mmol), TBAI (19 mg, 25 mol%). and lithium perchlorate (0.3 M) were taken in the cathodic chamber of an oven dry divided cell (separated by G3 sintered) and dissolved in 3 mL of MeCN. The anode chamber was charged with lithium perchlorate (0.3 M) and 3 mL of MeCN. The cell was equipped with graphite (5.2 cm × 0.8 cm × 0.2 cm) electrodes. The reaction mixture was continuously stirred and electrolyzed with an AXIOMET AX-3003P power at a constant current of 10 mA under air at 65 °C in an oil bath for 1 h. The progress of the reaction was monitored by TLC and evaporated under reduced pressure and ^{1}H NMR confirms no formation of **B1**.

8. Cyclic Voltammetry Experiment:

Cyclic Voltametric experiments were conducted on a “computer-controlled CH Instrument Electrochemical Analyzer [Klyte Research Model 263A]” in a three-electrode cell (beaker-type cell) at room temperature (25 ± 2 ºC) using Pt wire working electrode, Ag/AgCl as reference electrode, Glassy carbon electrode as the auxiliary electrode, and 0.1 M LiClO4 as electrolyte with 10 mM solution of the sample in MeCN. The surface area of the glassy carbon electrode is 7.06 sq. mm and the surface of the electrode is round shaped. The electrode is polished with figureeight motions on a cloth polishing pad in a water-alumina slurry. [Cp2Fe]+/0 couple was used as an internal standard and thereby the [Cp2Fe]+/0 couple potential was compared with the literature to acquire the actual potential of the catalyst vs SCE. For the cyclic voltammetric measurement, IUPAC convention was followed. The starting point for the CV curve was at 0.0 volt and measured in the positive direction. For the CV experiments, initial potential was 0.0 V, switching potential was +2.5 V and the scan rate was 100 mV/s. All solutions used for the voltametric experiments were deoxygenated by purging with high purity argon gas up to 5 mins and measurements were performed in open air at room temperature (25 ± 2 ºC). HPLC purity acetonitrile (MeCN) was purchased from Merck. The supporting electrolyte, lithium perchlorate ($LiClO_4$), was purchased from commercial suppliers TCI. Diagrams are standardized with respect to Fc/Fc^+.

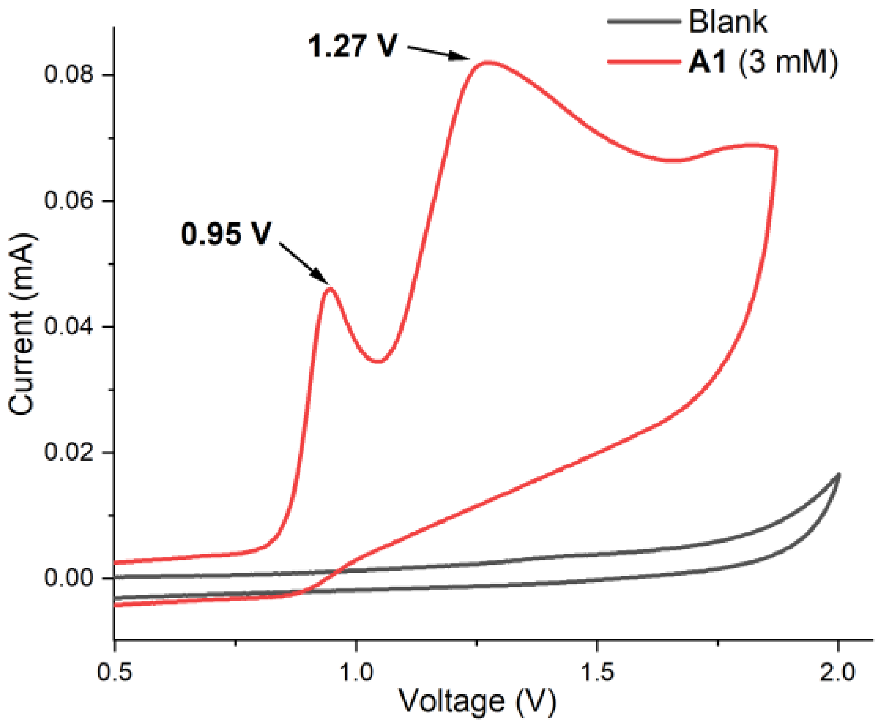
Blank
A1 (3 mM)
1.27 V
0.95 V
0.08
0.06
0.04
0.02
0.00
Current (mA)
0.5
1.0
1.5
2.0
Voltage (V)

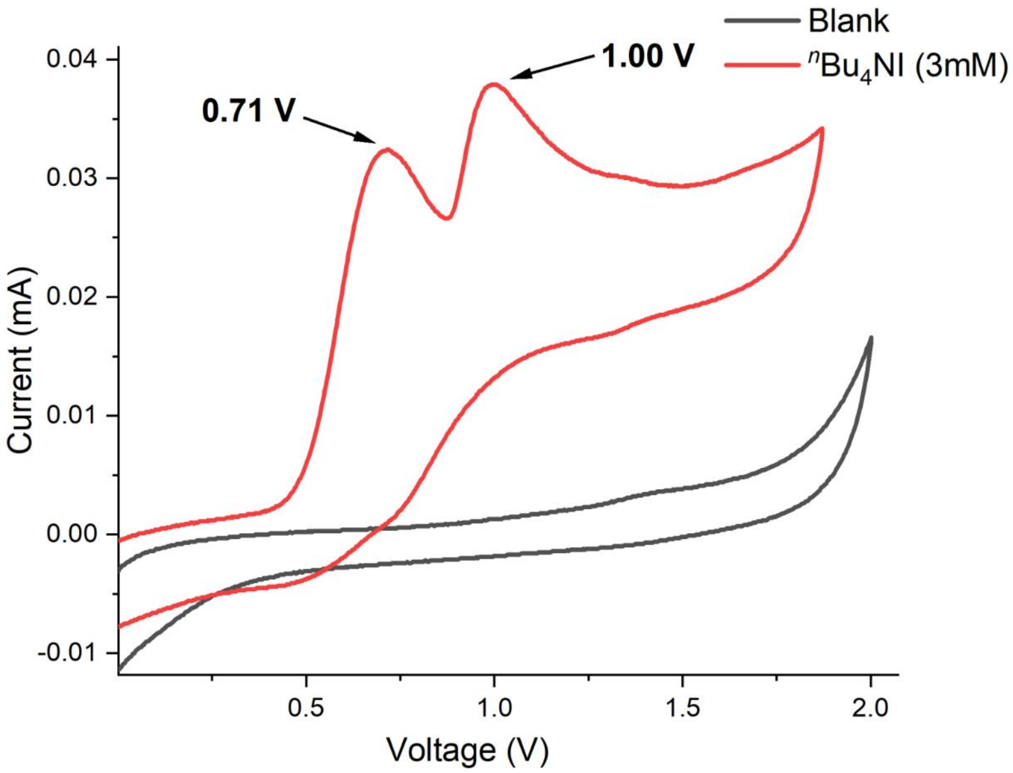
Blank
$^nBu_4NI$ (3mM)
1.00 V
0.71 V
0.04
0.03
0.02
0.01
0.00
-0.01
Current (mA)
0.5
1.0
1.5
2.0
Voltage (V)

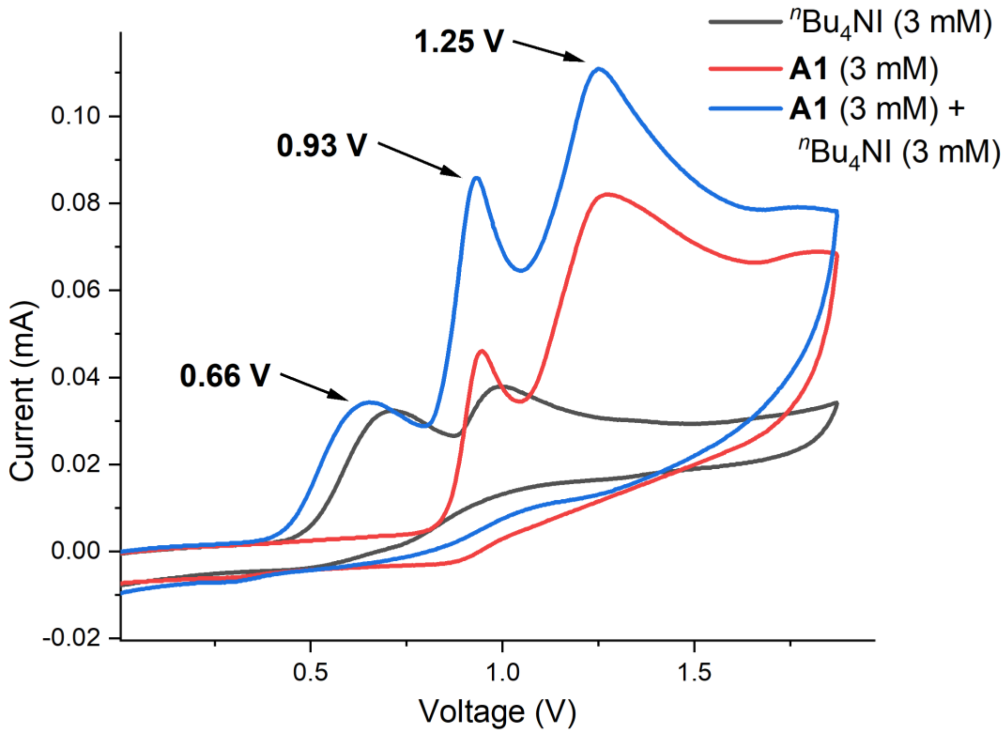

9) Failed Substrates:

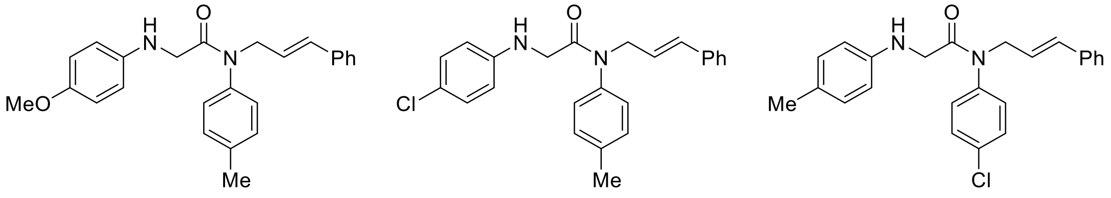

10) References:

(1) More, D. A.; Shinde, G. H.; Shaikh, A. C.; Muthukrishnan, M. *RSC Adv.* **2019**, *9*, 30277–30291.

(2) Dong, W.; Hu, B.; Gao, X.; Li, Y.; Xie, X.; Zhang, Z. *J. Org. Chem.* **2016,** *81*, 8770–8776.

(3) Xing, P.; Zang, W.; Huang, Z. G.; Zhan, Y. X.; Zhu, C. J.; Jiang, B. A Mild Method for Indium(III)-Catalyzed 1,4-Hydrosilylation of α,β-Enone Esters with Triethylsilane and Trifluoroacetic Acid. *Synlett* **2012**, *23*, 2269–2273.

(4) Gandomkar, S.; Jost, E.; Loidolt, D.; Swoboda, A.; Pickl, M.; Elaily, W.; Daniel, B.; Fraaije, M. W.; Macheroux, P.; Kroutil, W. *Adv. Synth. Catal.* **2019**, *361*, 5264–5271.

(5) Einaru, S.; Shitamichi, K.; Nagano, T.; Matsumoto, A.; Asano, K.; Matsubara, S. *Angew. Chem. Int. Ed.* **2018**, *57*, 13863–13867.

11) NMR Spectra of Synthesized Compounds:

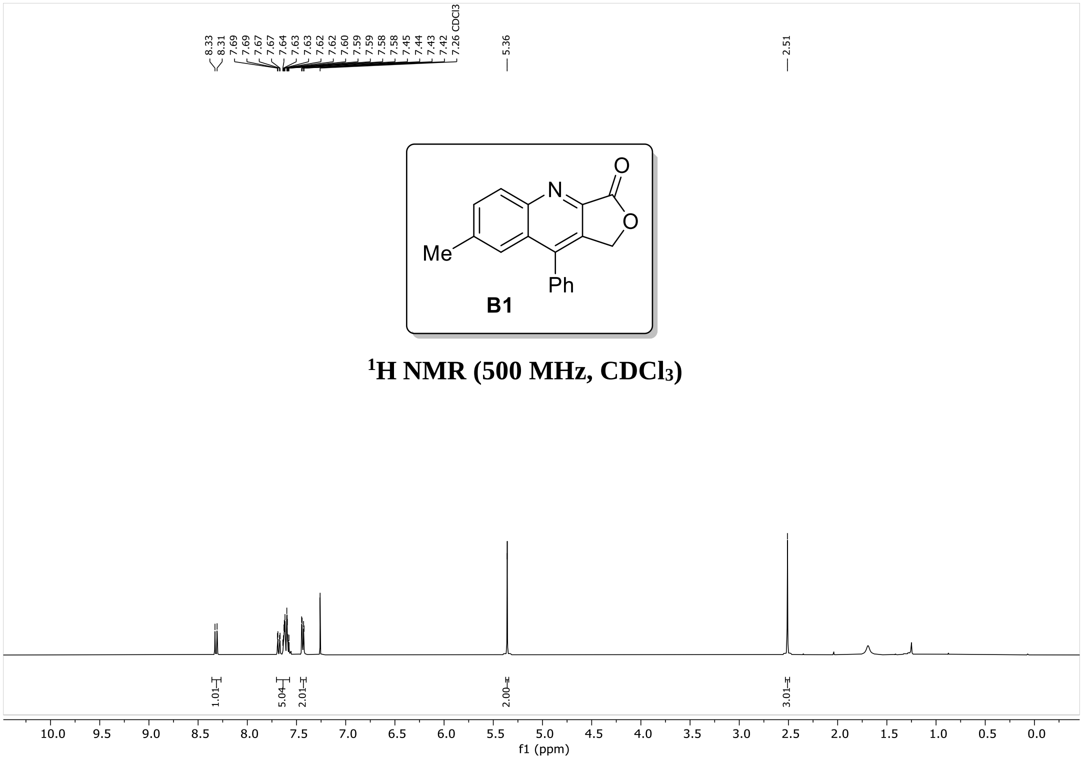

^{1}H NMR (500 MHz, $CDCl_3$)

.

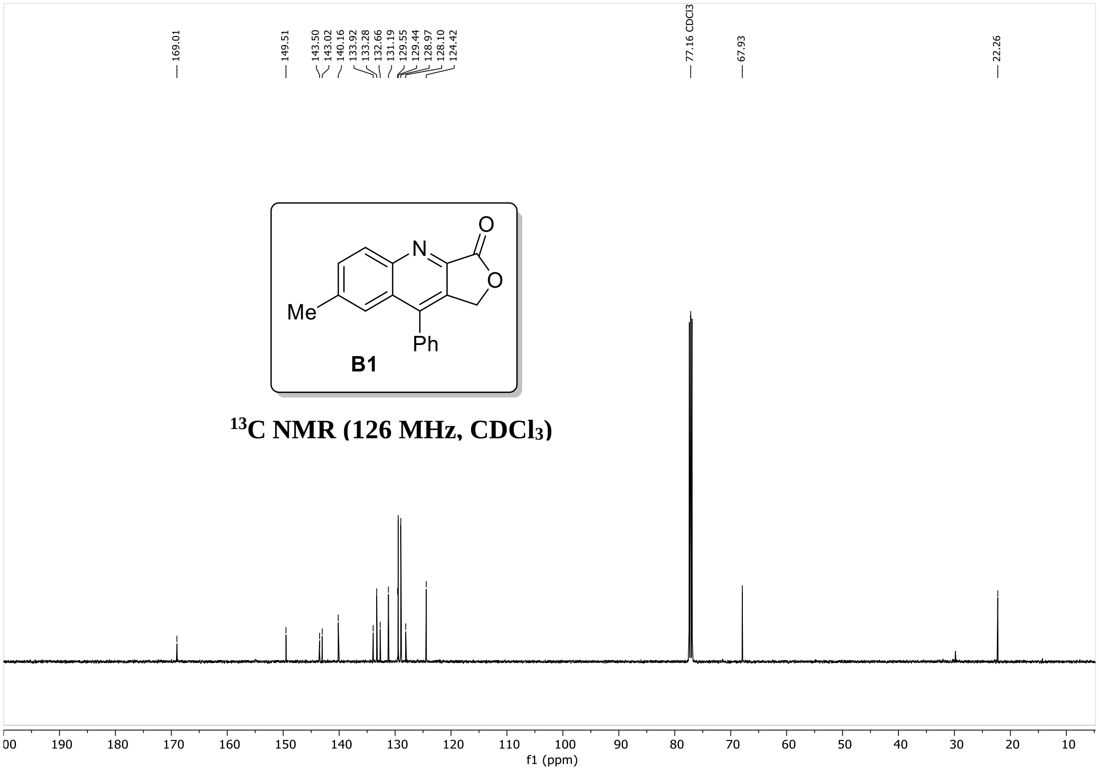

^{13}C NMR (126 MHz, $CDCl_3$)

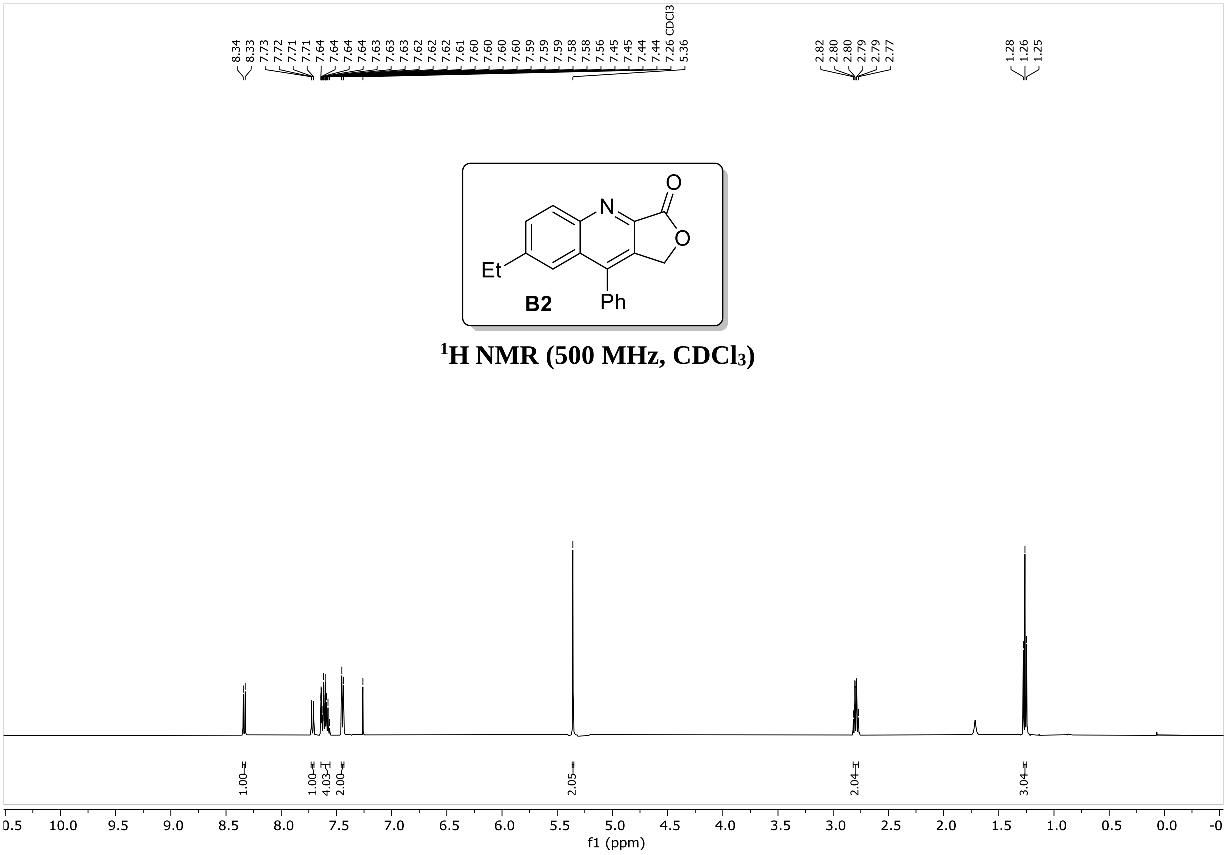

B2
Et
Ph
N
O
1H NMR (500 MHz, CDCl3)
f1 (ppm)

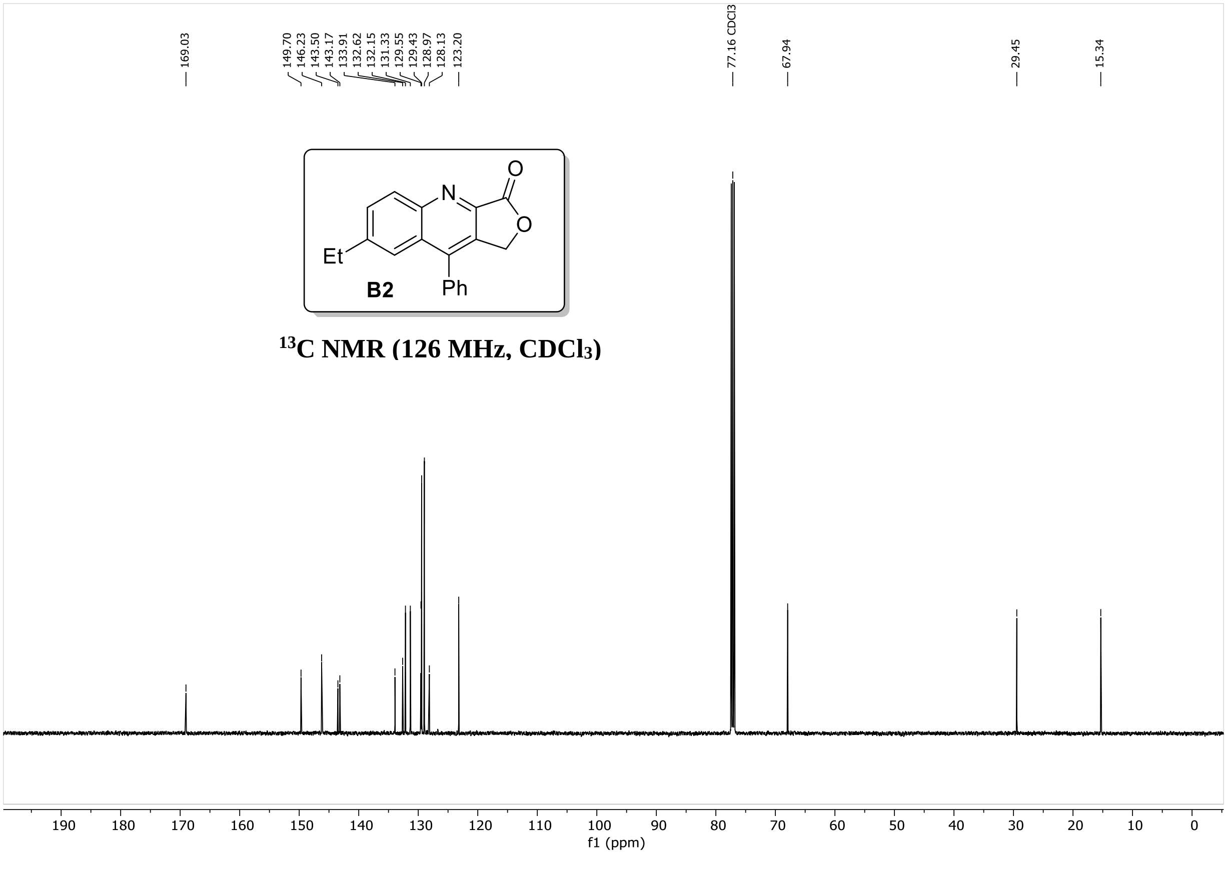

B2
Et
Ph
N
O
13C NMR (126 MHz, CDCl3)
f1 (ppm)

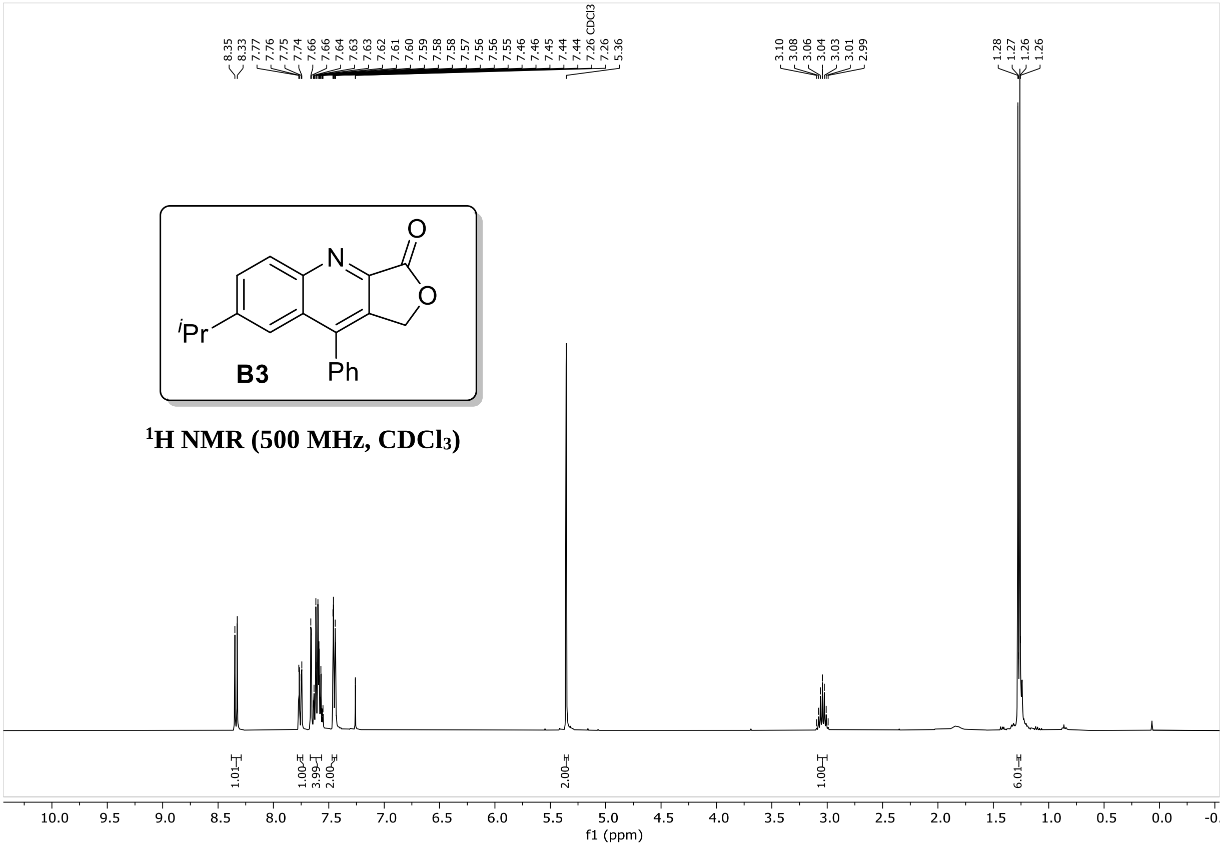
8.35
8.33
7.77
7.76
7.75
7.74
7.66
7.66
7.64
7.63
7.63
7.62
7.61
7.60
7.59
7.58
7.58
7.57
7.56
7.56
7.55
7.46
7.46
7.45
7.45
7.44
7.44
7.26 CDCl3
7.26
5.36
3.10
3.08
3.06
3.04
3.03
3.01
2.99
1.28
1.27
1.26
1.26
N
O
O
iPr
B3
Ph
1H NMR (500 MHz, CDCl3)
1.01
1.00
3.99
2.00
2.00
1.00
6.01
f1 (ppm)

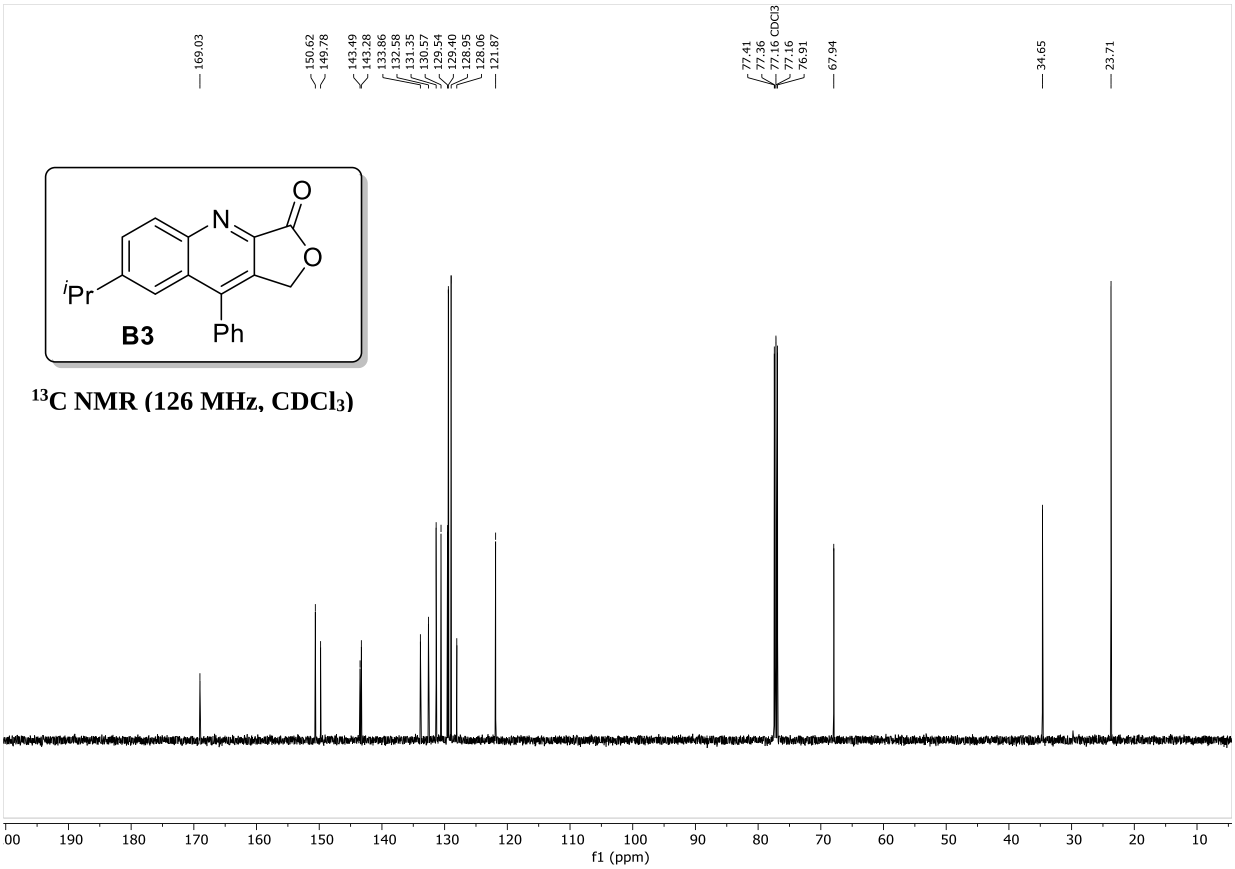
169.03
150.62
149.78
143.49
143.28
133.86
132.58
131.35
130.57
129.54
129.40
128.95
128.06
121.87
77.41
77.36
77.16 CDCl3
77.16
76.91
67.94
34.65
23.71
N
O
O
iPr
B3
Ph
13C NMR (126 MHz, CDCl3)
f1 (ppm)

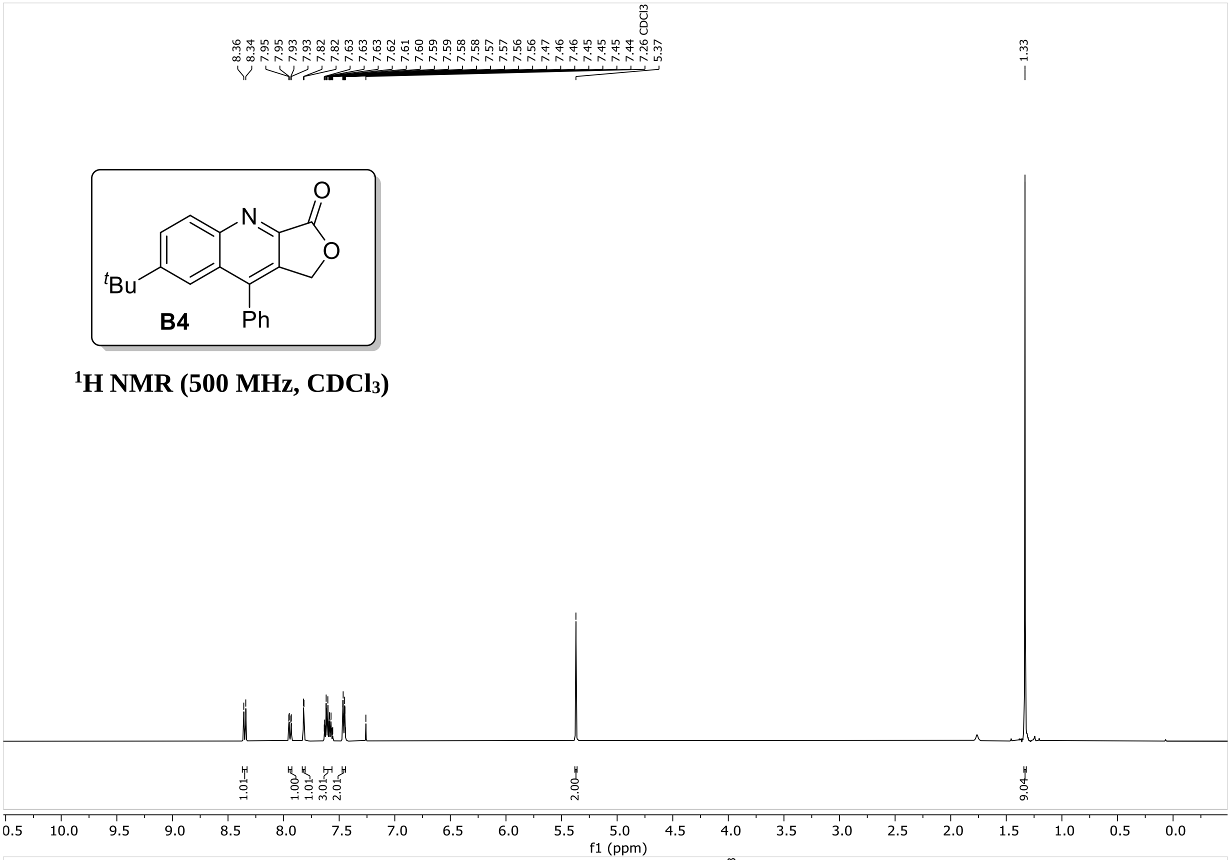

^{1}H NMR (500 MHz, CDCl$_3$)

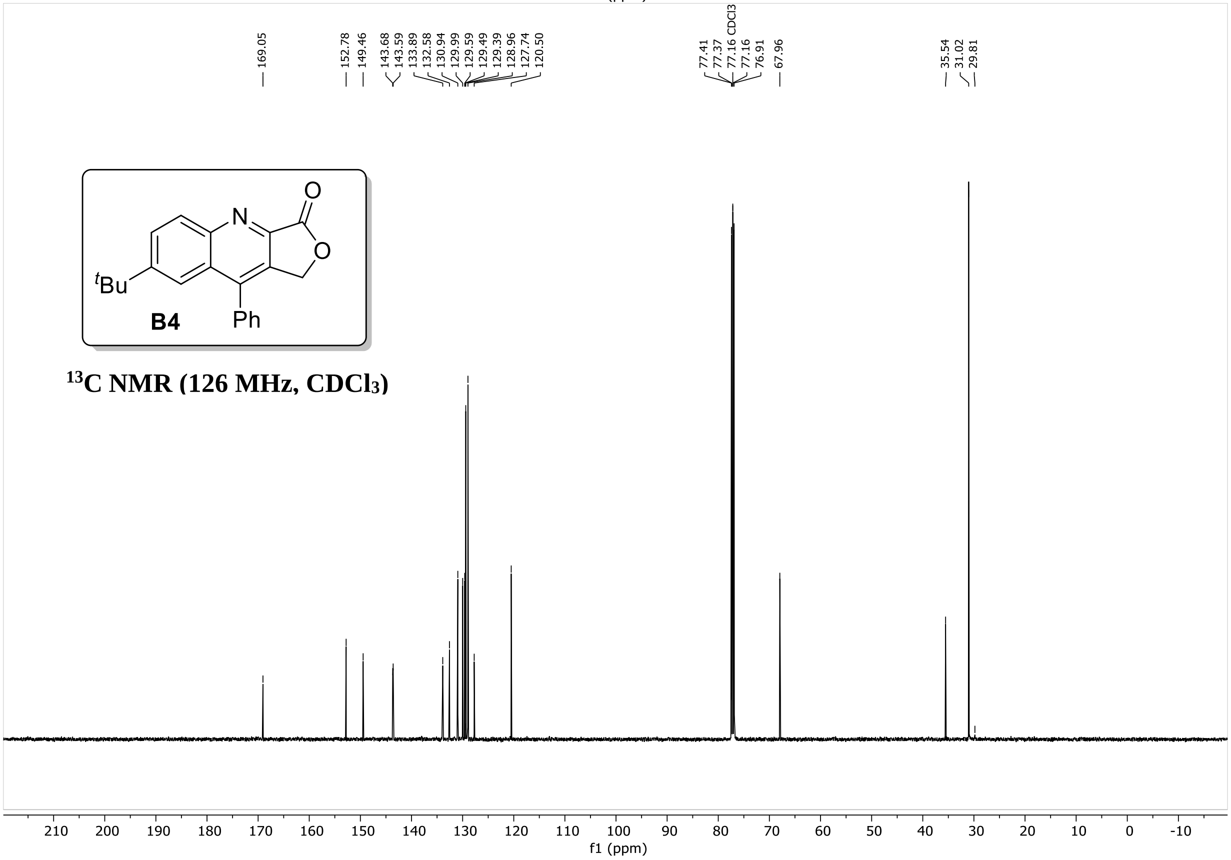

^{13}C NMR (126 MHz, CDCl$_3$)

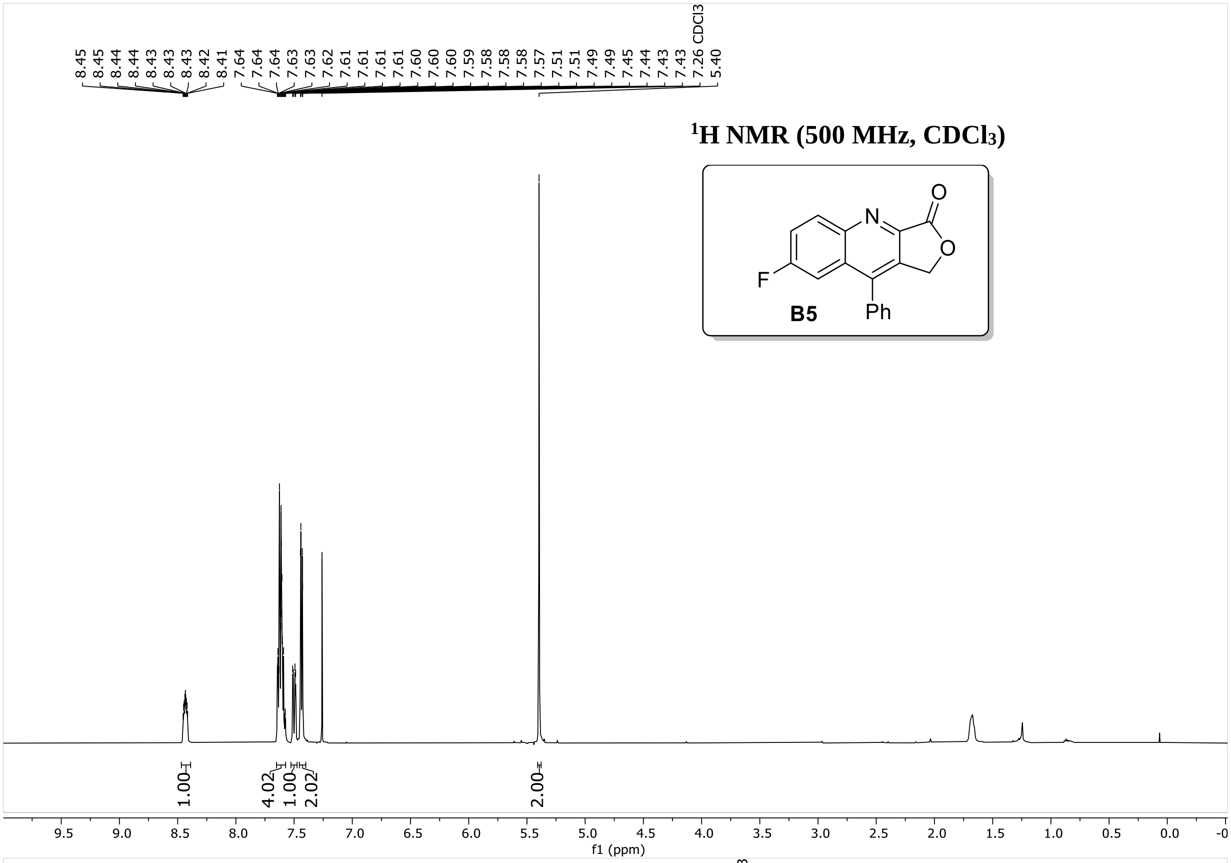
1H NMR (500 MHz, CDCl3)
B5
Ph
F
N
O
f1 (ppm)

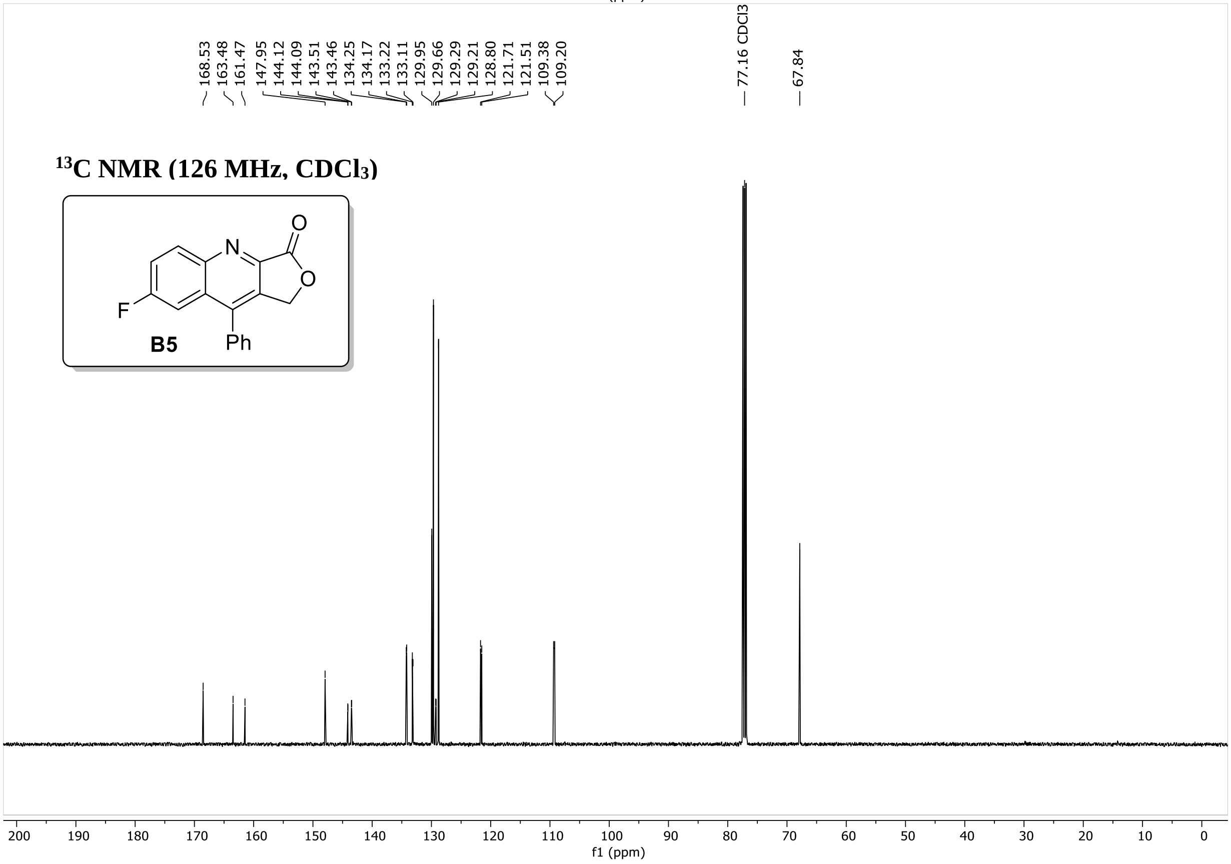
13C NMR (126 MHz, CDCl3)
B5
Ph
F
N
O
f1 (ppm)

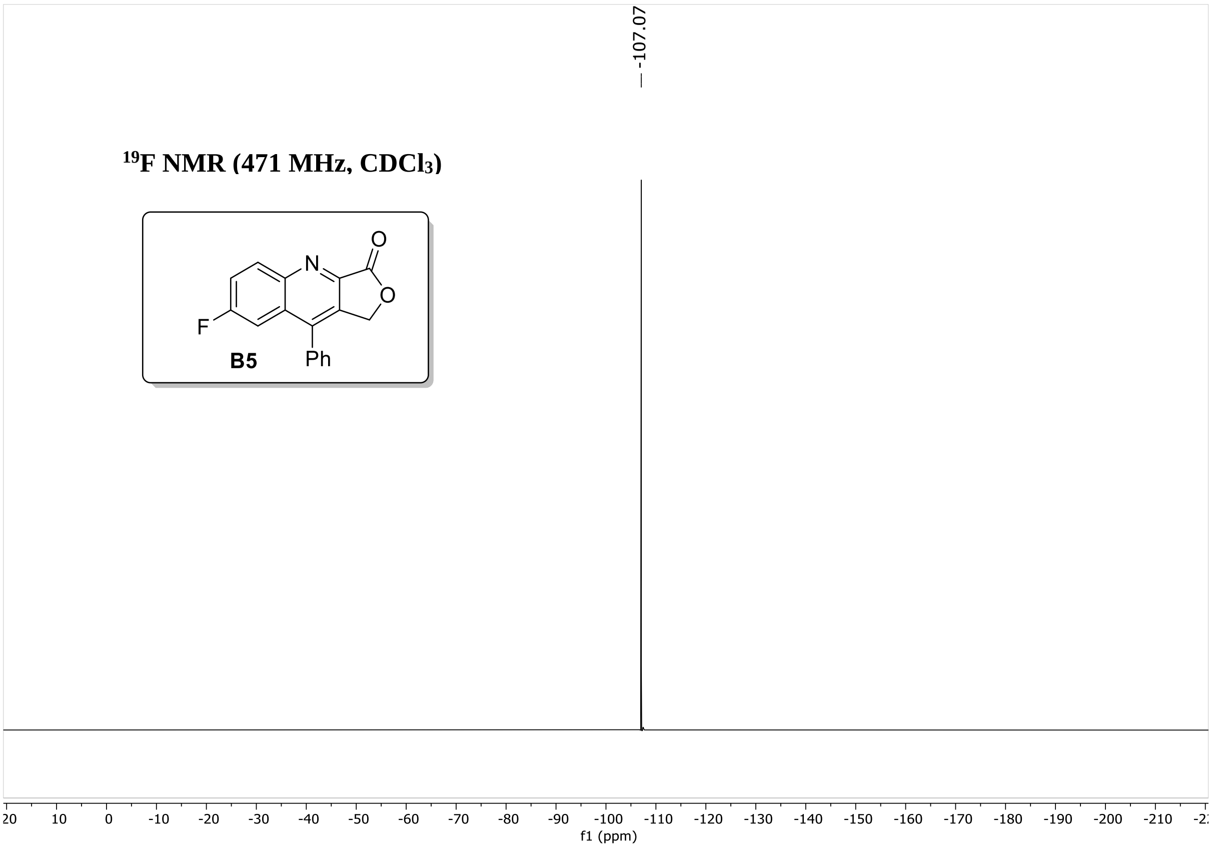
-107.07
19F NMR (471 MHz, CDCl3)
N
O
O
F
B5
Ph
20 10 0 -10 -20 -30 -40 -50 -60 -70 -80 -90 -100 -110 -120 -130 -140 -150 -160 -170 -180 -190 -200 -210
f1 (ppm)

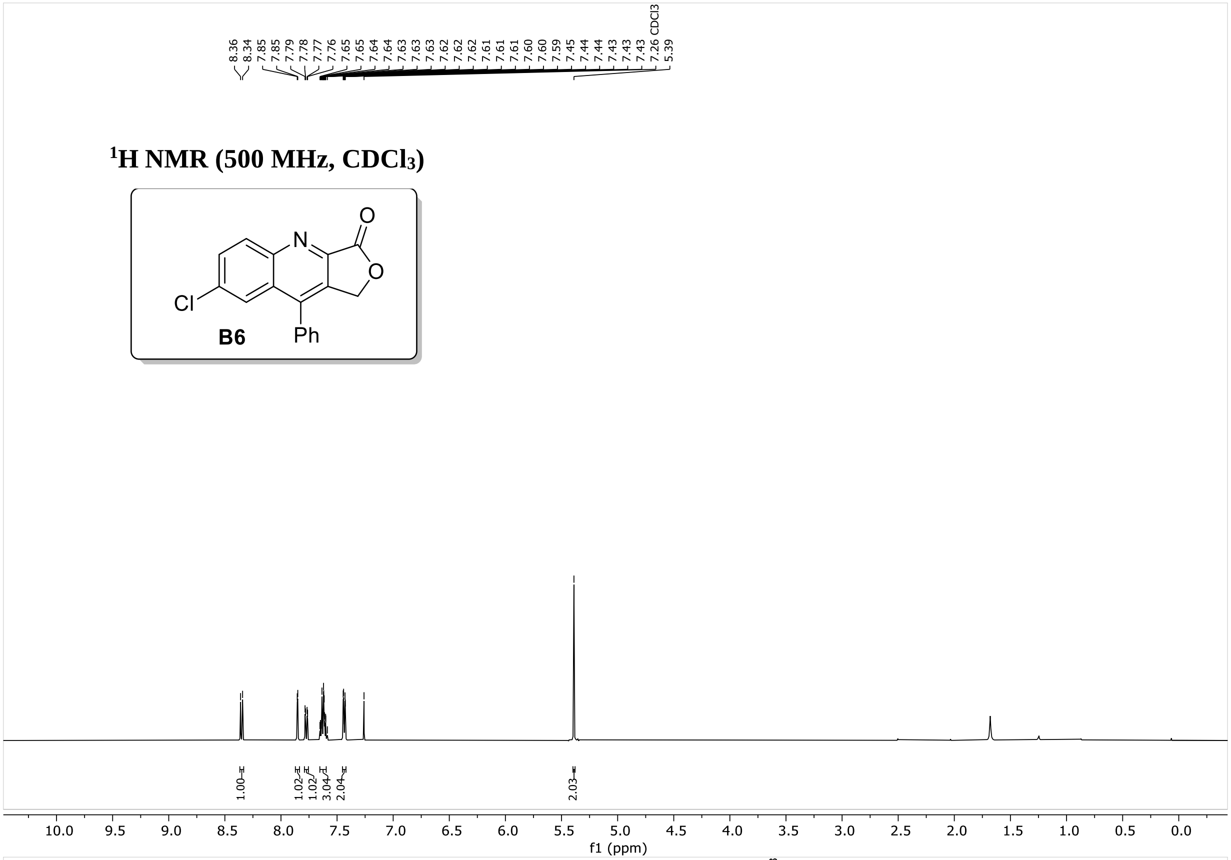

8.36
8.34
7.85
7.85
7.79
7.78
7.77
7.76
7.65
7.65
7.64
7.63
7.63
7.63
7.62
7.62
7.61
7.61
7.61
7.60
7.60
7.59
7.45
7.44
7.44
7.43
7.43
7.43
7.26 CDCl3
5.39
¹H NMR (500 MHz, CDCl₃)
N
O
O
Cl
B6
Ph
1.00
1.02
1.02
3.04
2.04
2.03
10.0
9.5
9.0
8.5
8.0
7.5
7.0
6.5
6.0
5.5
5.0
4.5
4.0
3.5
3.0
2.5
2.0
1.5
1.0
0.5
0.0
f1 (ppm)

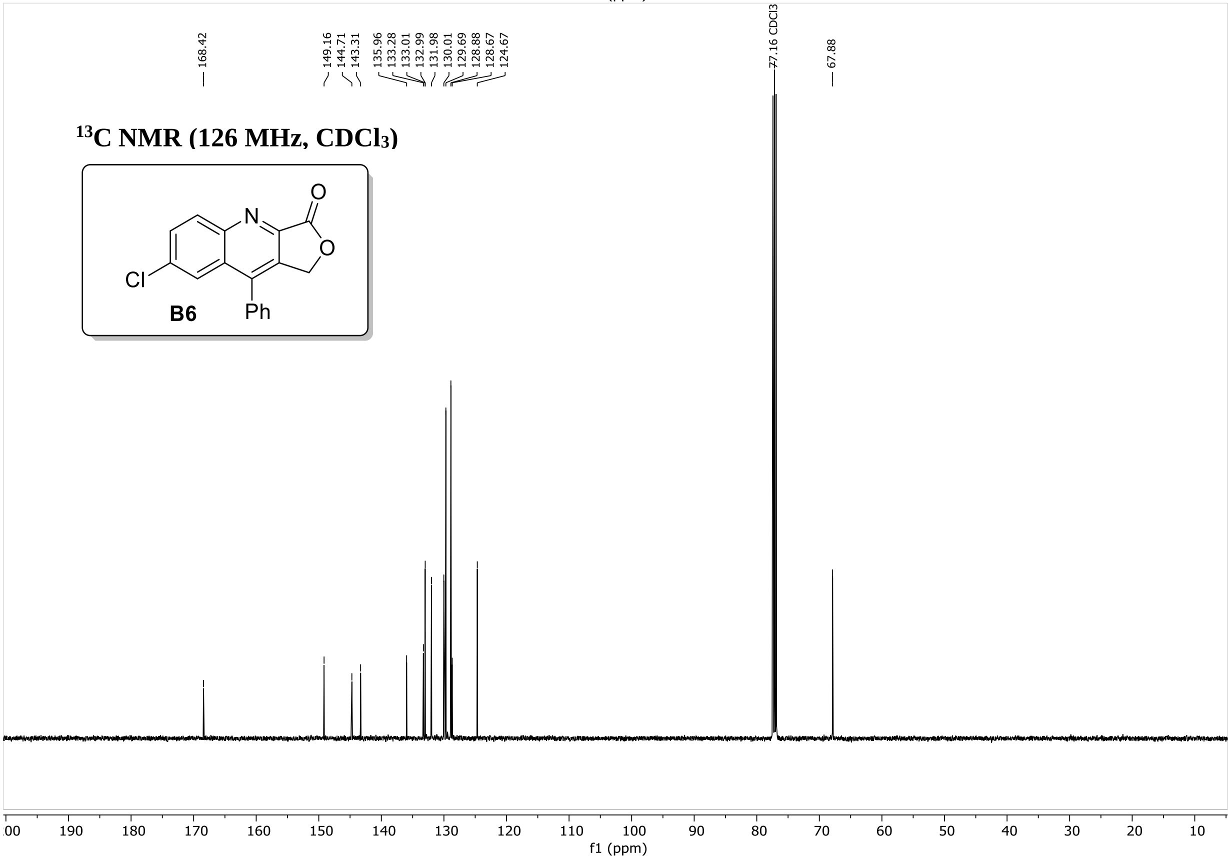

168.42
149.16
144.71
143.31
135.96
133.28
133.01
132.99
131.98
130.01
129.69
128.88
128.67
124.67
77.16 CDCl3
67.88
¹³C NMR (126 MHz, CDCl₃)
N
O
O
Cl
B6
Ph
00
190
180
170
160
150
140
130
120
110
100
90
80
70
60
50
40
30
20
10
f1 (ppm)

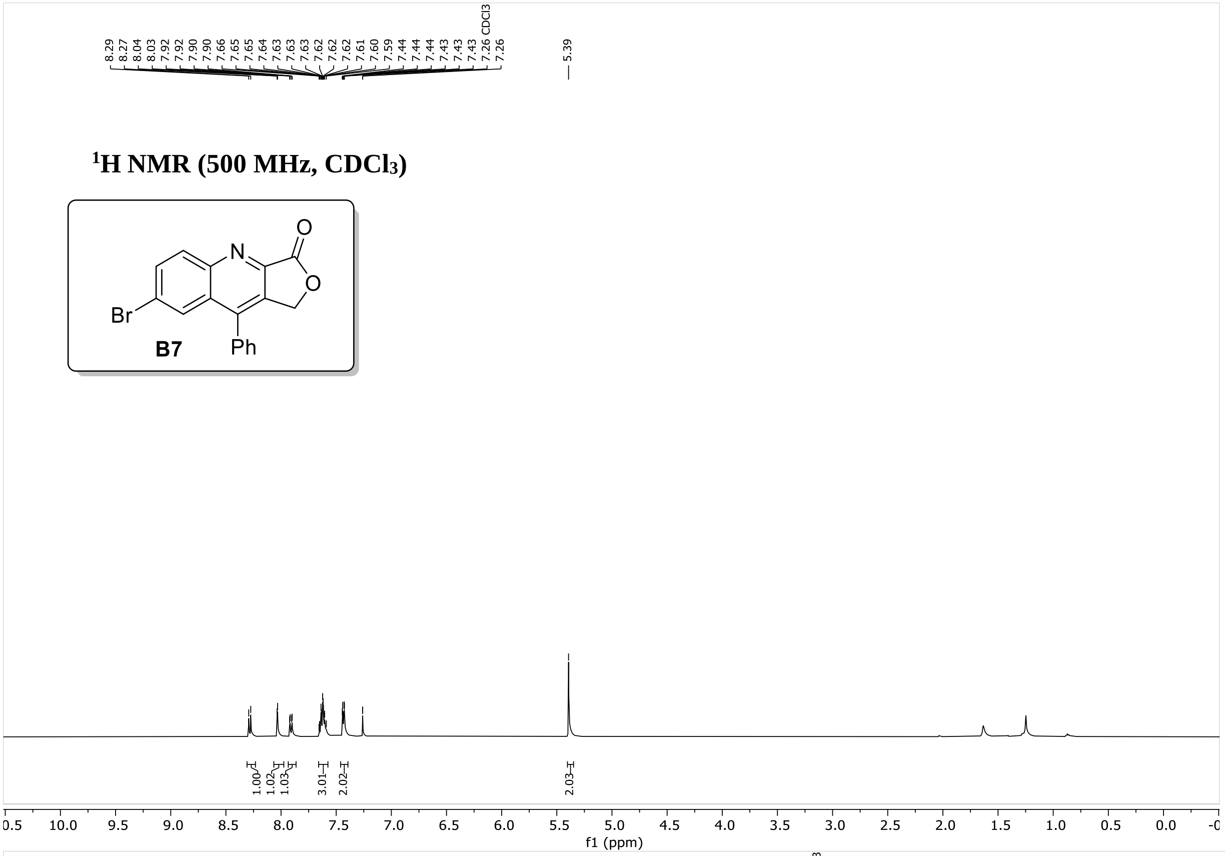
8.29
8.27
8.04
8.03
7.92
7.92
7.90
7.90
7.66
7.65
7.65
7.64
7.63
7.63
7.63
7.62
7.62
7.62
7.61
7.60
7.59
7.44
7.44
7.44
7.43
7.43
7.43
7.26 CDCl3
7.26
5.39
¹H NMR (500 MHz, CDCl₃)
Br
N
O
O
B7
Ph
1.00
1.02
1.03
3.01
2.02
2.03
f1 (ppm)

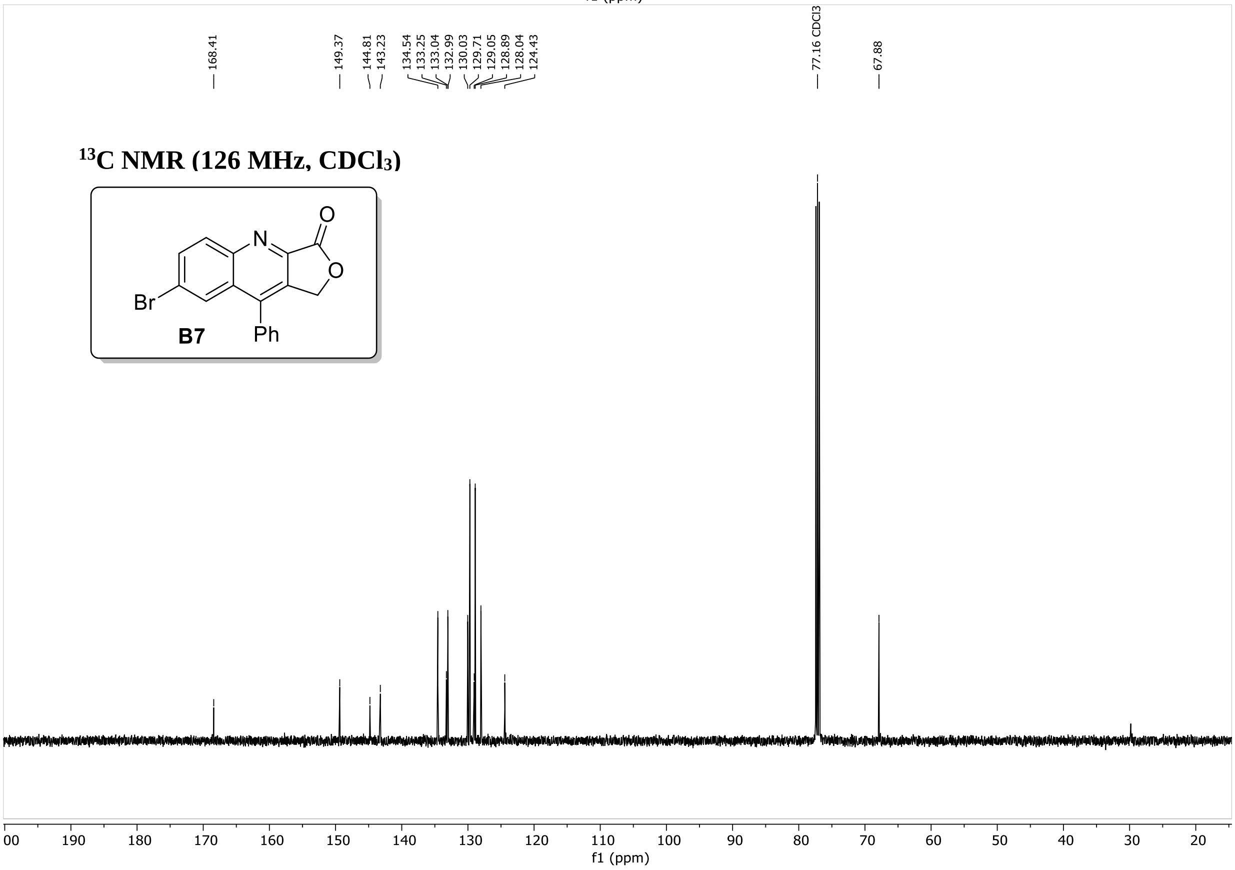
168.41
149.37
144.81
143.23
134.54
133.25
133.04
132.99
130.03
129.71
129.05
128.89
128.04
124.43
77.16 CDCl3
67.88
¹³C NMR (126 MHz, CDCl₃)
Br
N
O
O
B7
Ph
f1 (ppm)

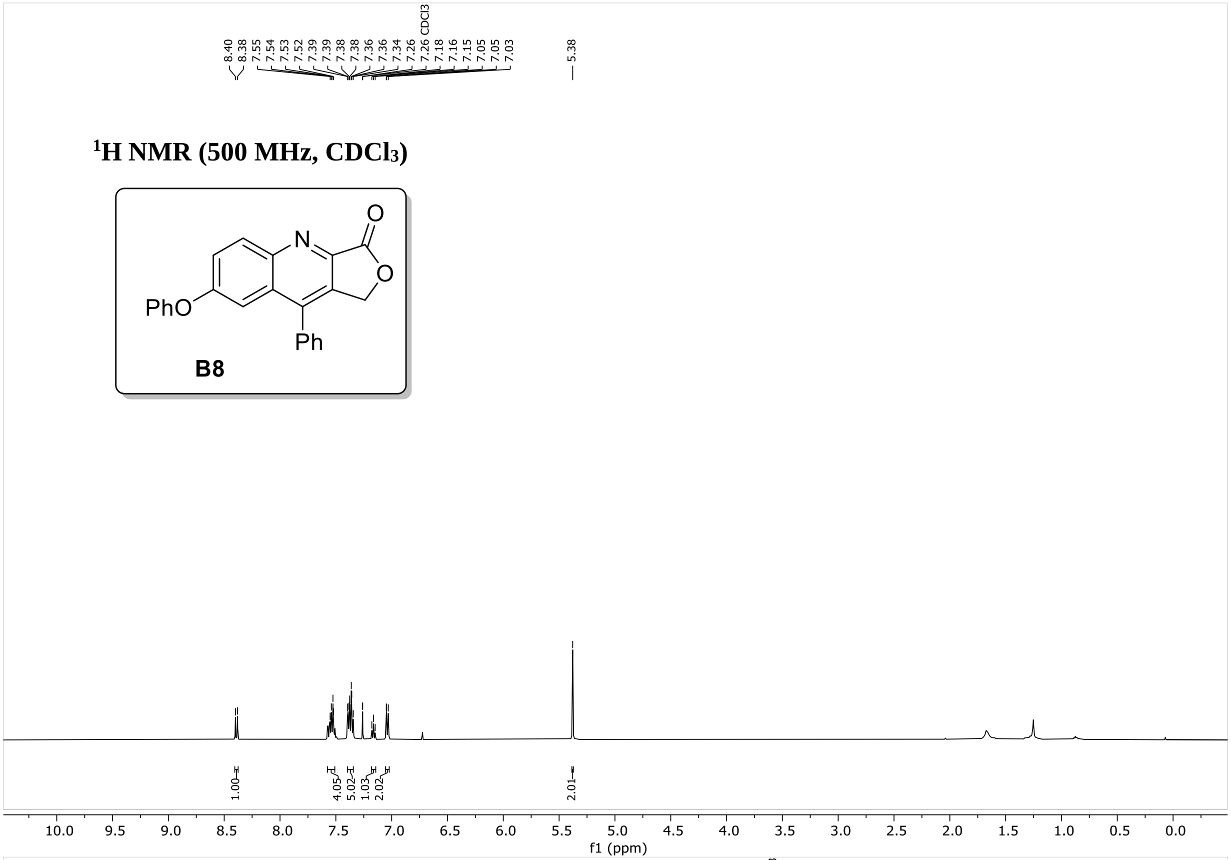
8.40
8.38
7.55
7.54
7.53
7.52
7.39
7.39
7.38
7.38
7.36
7.36
7.34
7.26 CDCl3
7.18
7.16
7.15
7.05
7.05
7.03
5.38
¹H NMR (500 MHz, CDCl₃)
N
O
O
PhO
Ph
B8
1.00
4.05
5.02
1.03
2.02
2.01
f1 (ppm)

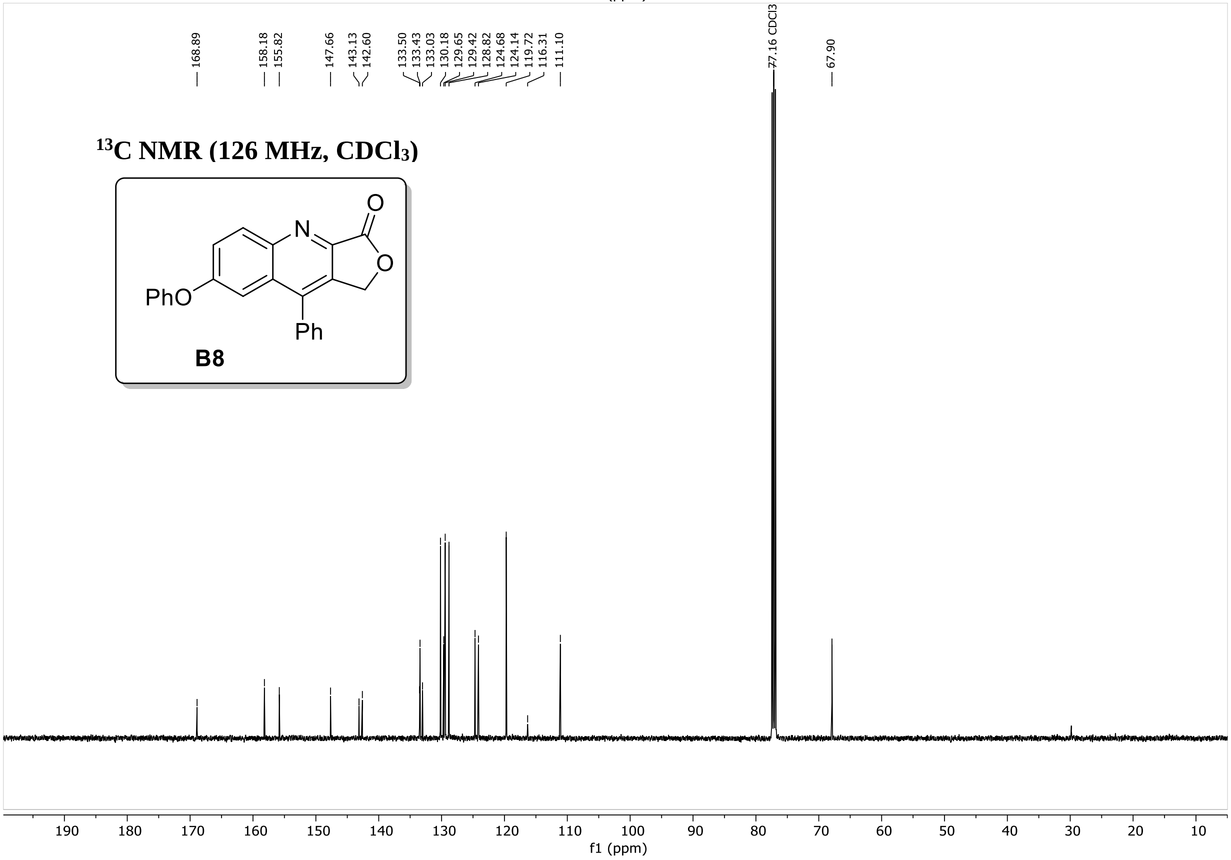
168.89
158.18
155.82
147.66
143.13
142.60
133.50
133.43
133.03
130.18
129.65
129.42
128.82
124.68
124.14
119.72
116.31
111.10
77.16 CDCl3
67.90
¹³C NMR (126 MHz, CDCl₃)
N
O
O
PhO
Ph
B8
f1 (ppm)

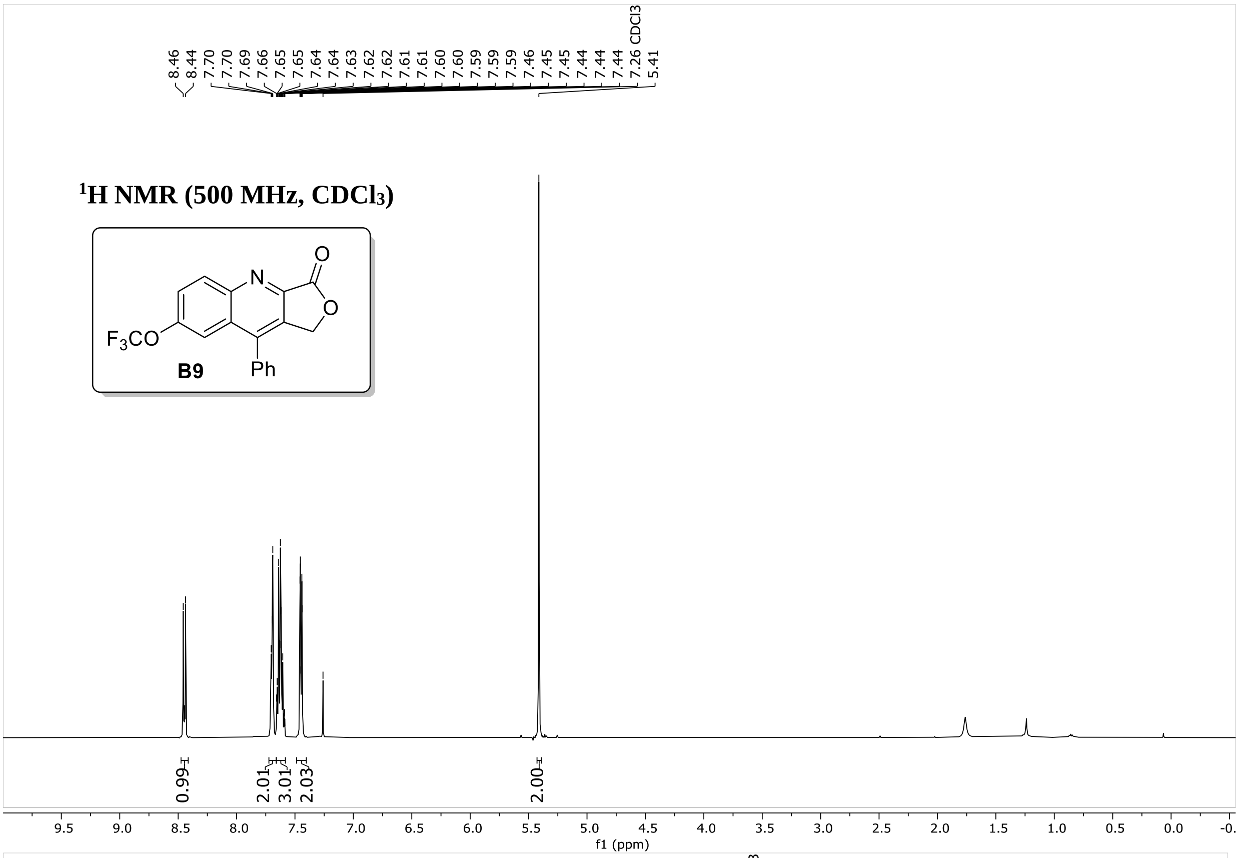

1H NMR (500 MHz, CDCl3)
F3CO
B9
Ph
f1 (ppm)

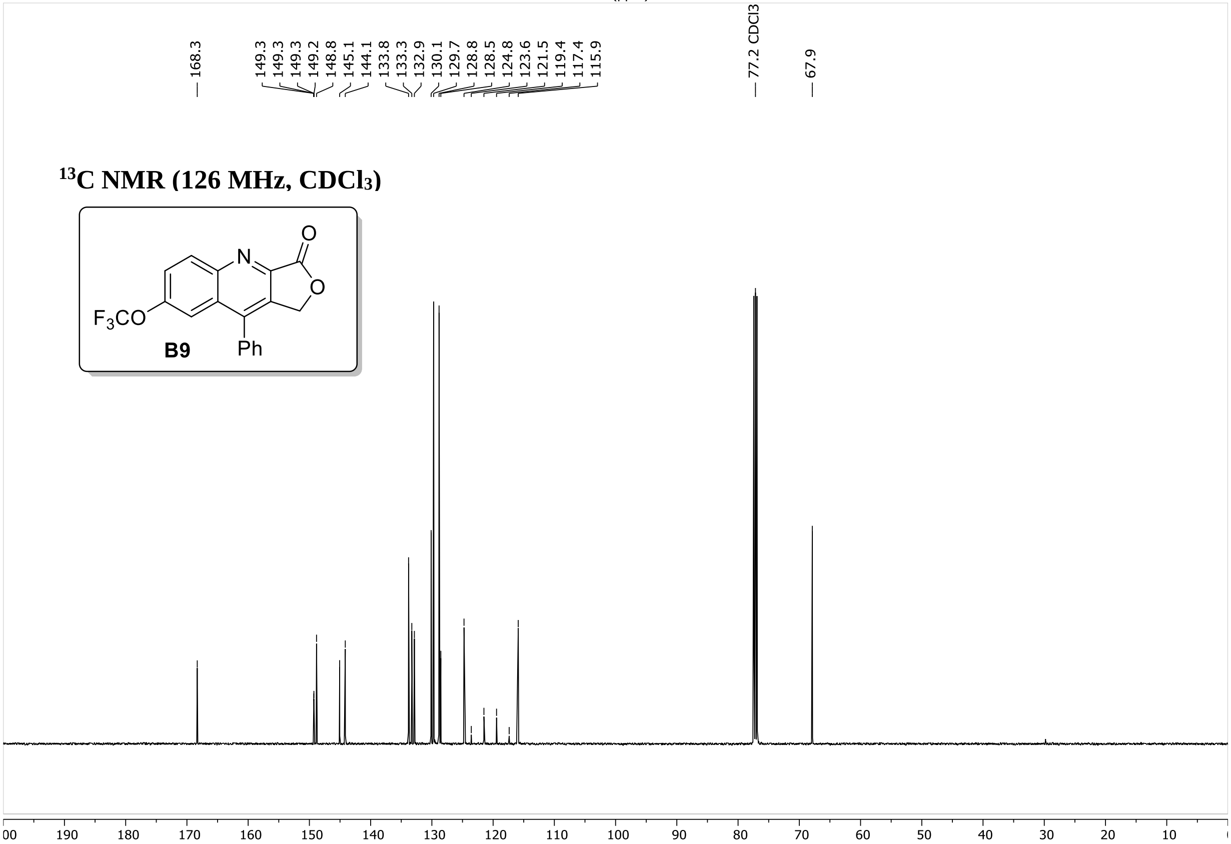

13C NMR (126 MHz, CDCl3)
F3CO
B9
Ph
f1 (ppm)

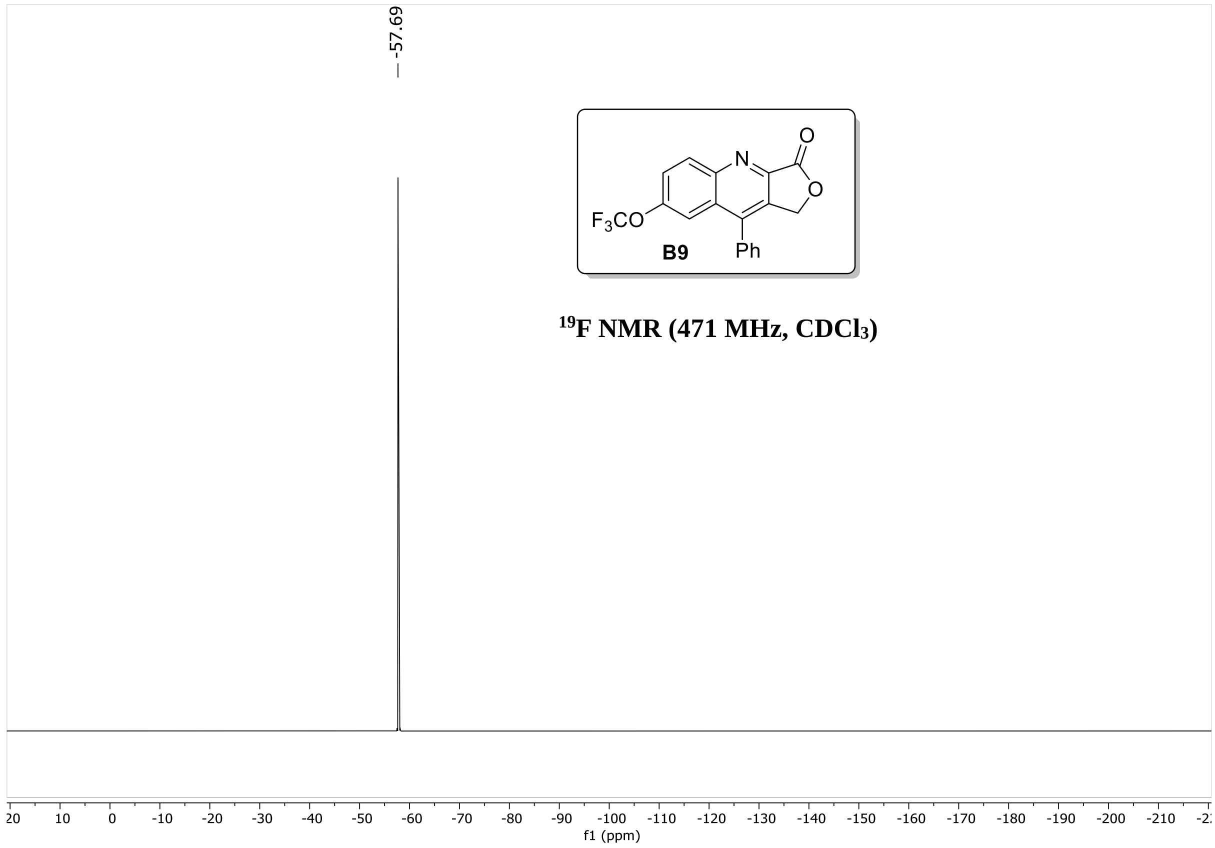
-57.69
N
O
O
F3CO
B9
Ph
19F NMR (471 MHz, CDCl3)
20 10 0 -10 -20 -30 -40 -50 -60 -70 -80 -90 -100 -110 -120 -130 -140 -150 -160 -170 -180 -190 -200 -210
f1 (ppm)

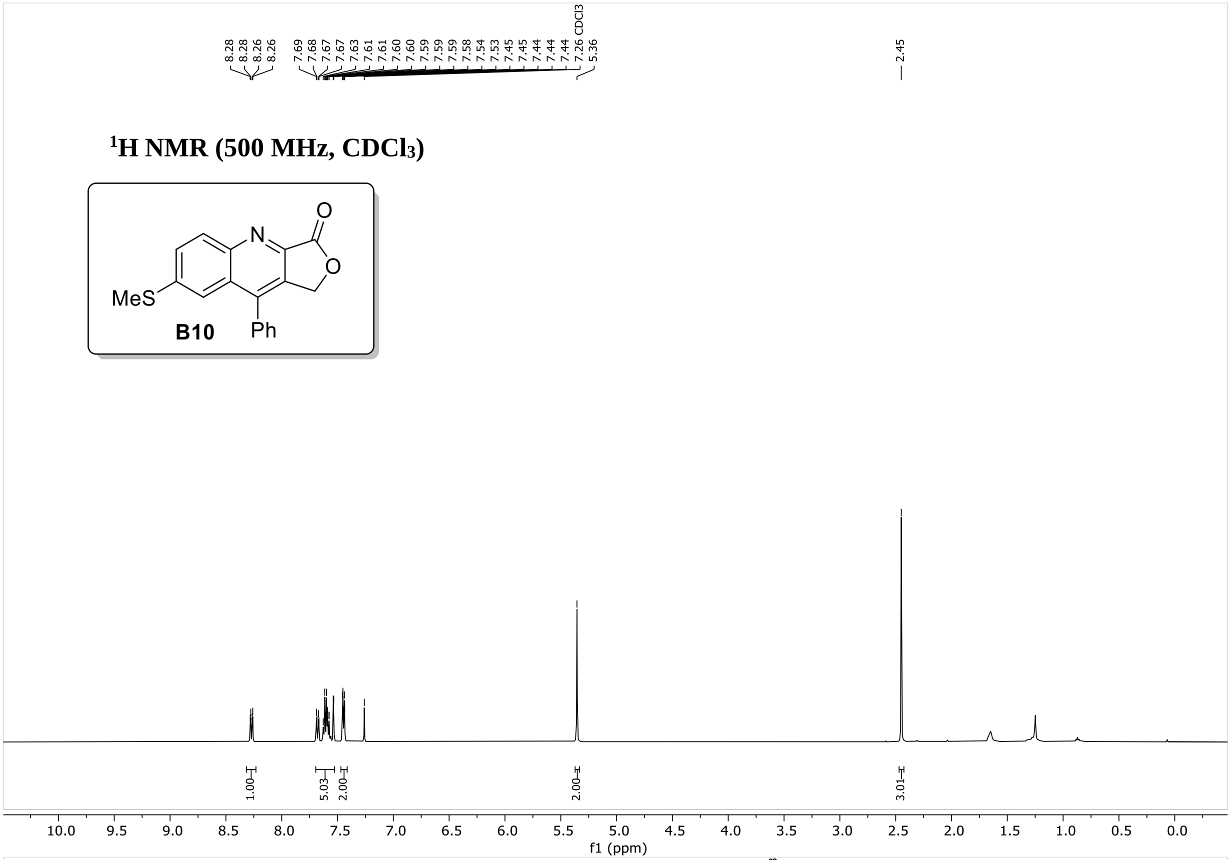

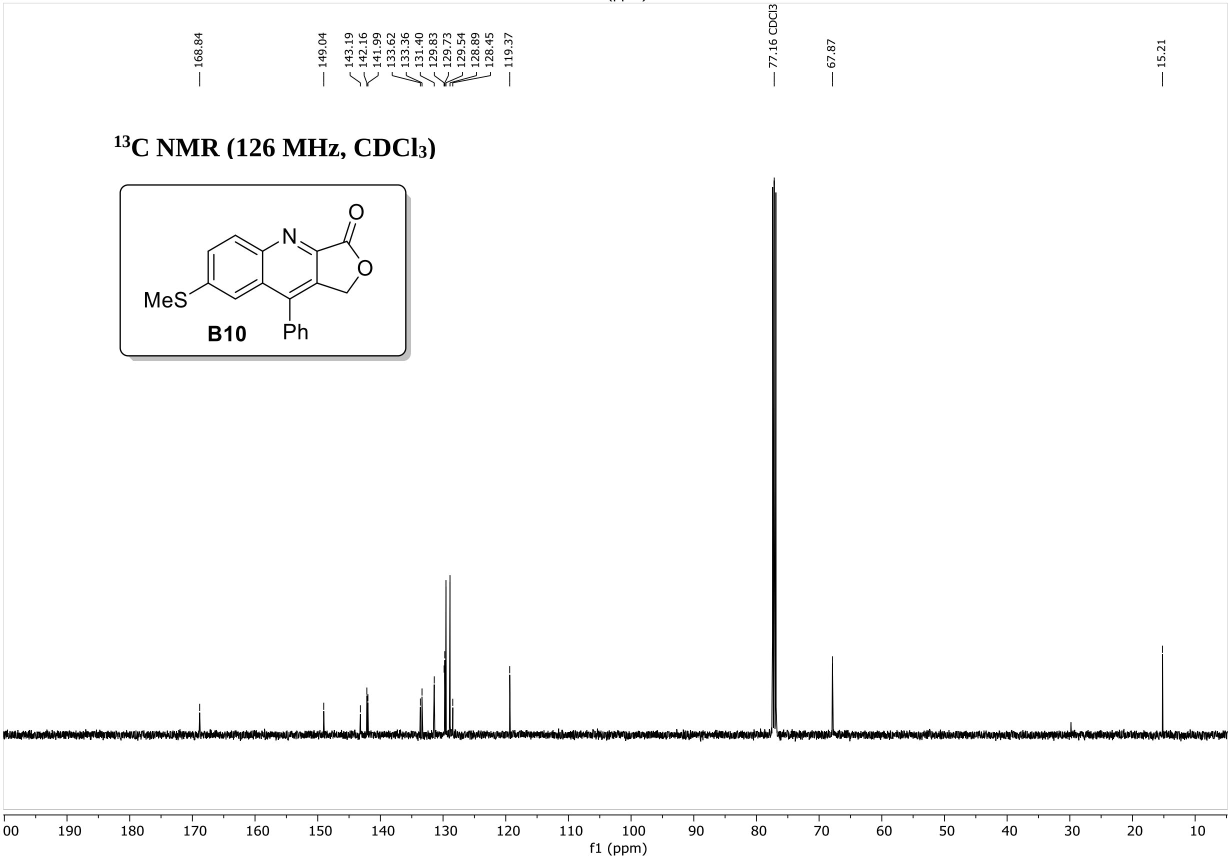

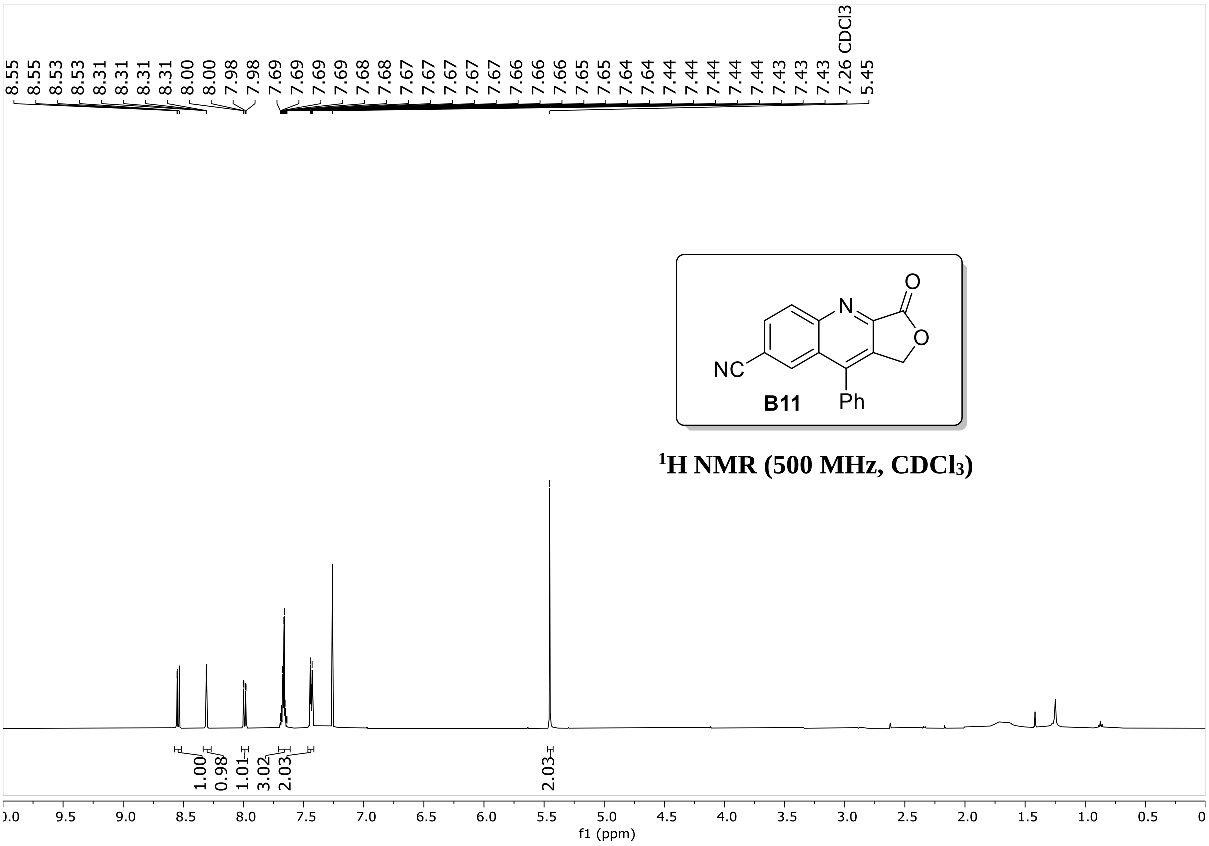

^{1}H NMR (500 MHz, $CDCl_3$)

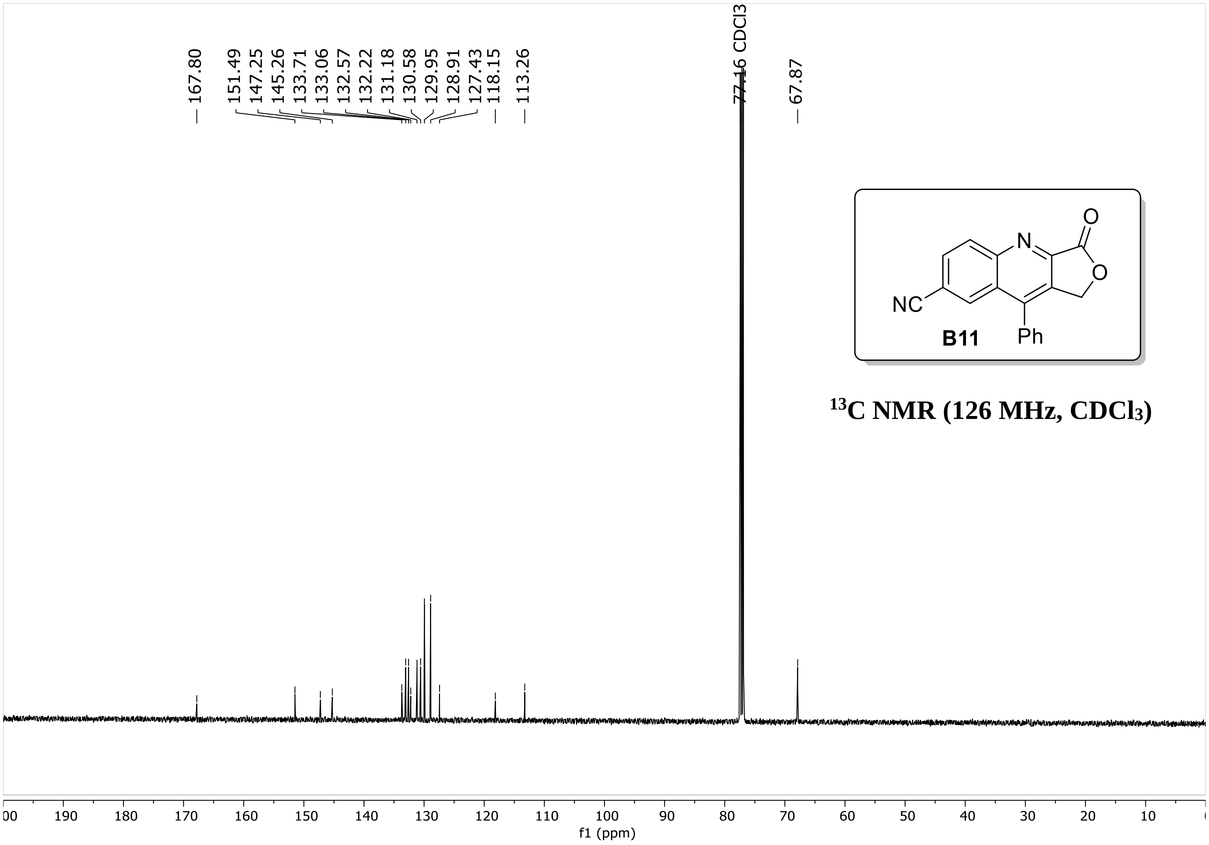

^{13}C NMR (126 MHz, $CDCl_3$)

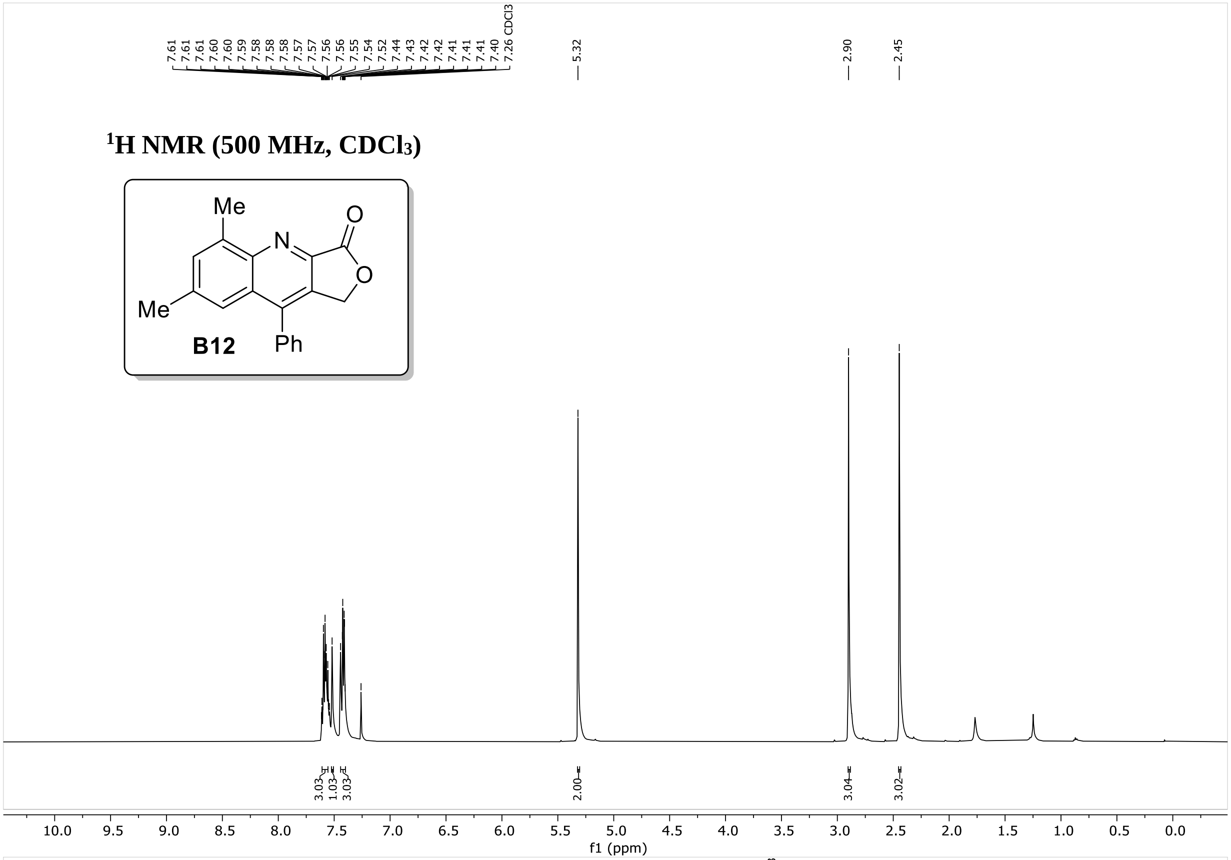

7.61
7.61
7.61
7.60
7.59
7.58
7.58
7.58
7.57
7.57
7.56
7.56
7.55
7.54
7.52
7.44
7.43
7.42
7.42
7.41
7.41
7.41
7.40
7.26 CDCl3
5.32
2.90
2.45
1H NMR (500 MHz, CDCl3)
Me
N
O
O
Me
B12
Ph
3.03
1.03
3.03
2.00
3.04
3.02
10.0 9.5 9.0 8.5 8.0 7.5 7.0 6.5 6.0 5.5 5.0 4.5 4.0 3.5 3.0 2.5 2.0 1.5 1.0 0.5 0.0
f1 (ppm)

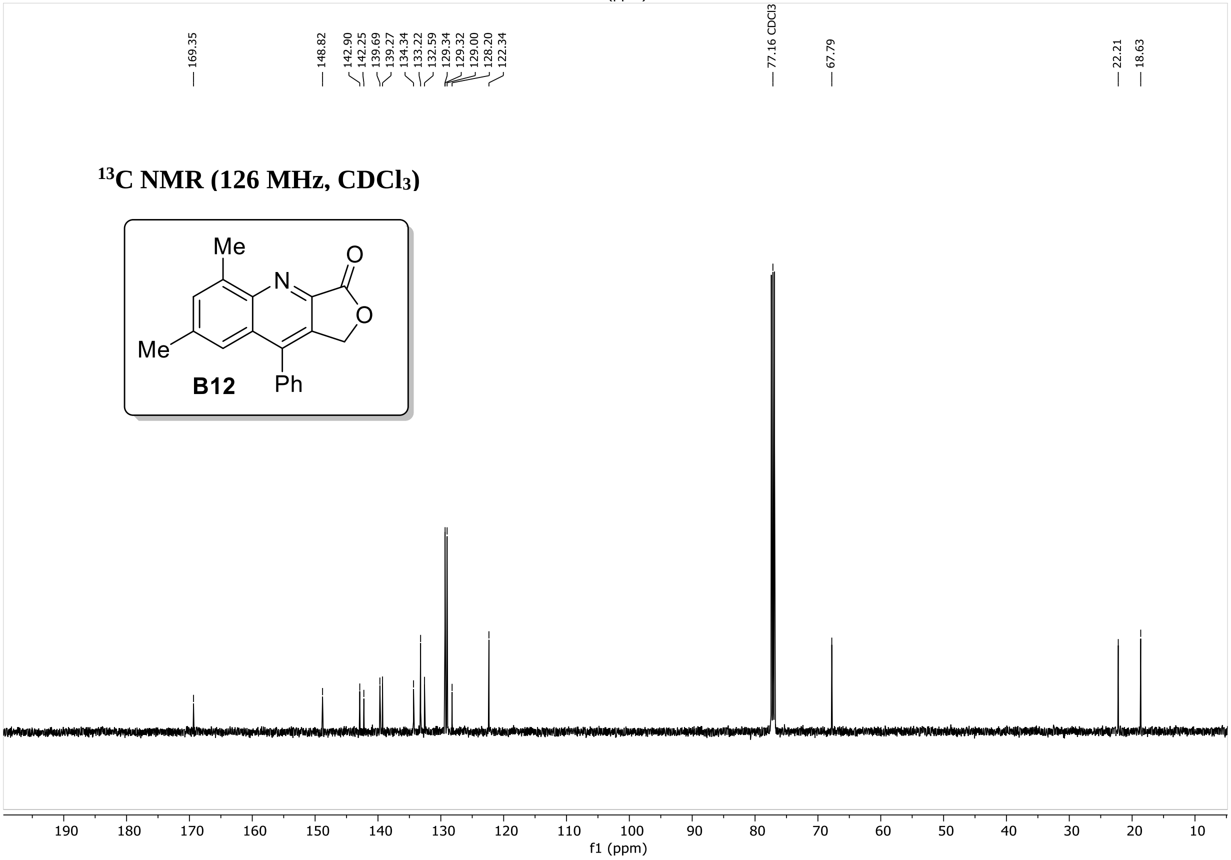

169.35
148.82
142.90
142.25
139.69
139.27
134.34
133.22
132.59
129.34
129.32
129.00
128.20
122.34
77.16 CDCl3
67.79
22.21
18.63
13C NMR (126 MHz, CDCl3)
Me
N
O
O
Me
B12
Ph
190 180 170 160 150 140 130 120 110 100 90 80 70 60 50 40 30 20 10
f1 (ppm)

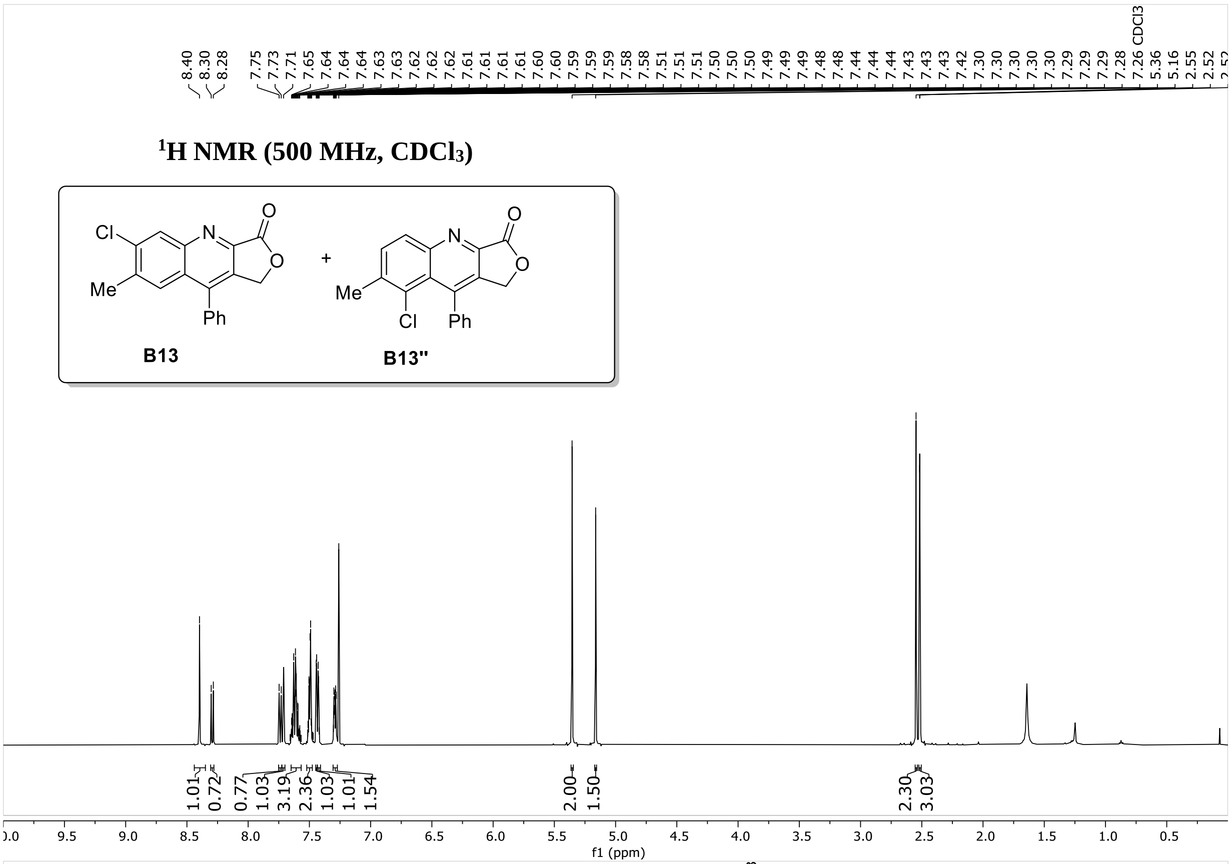

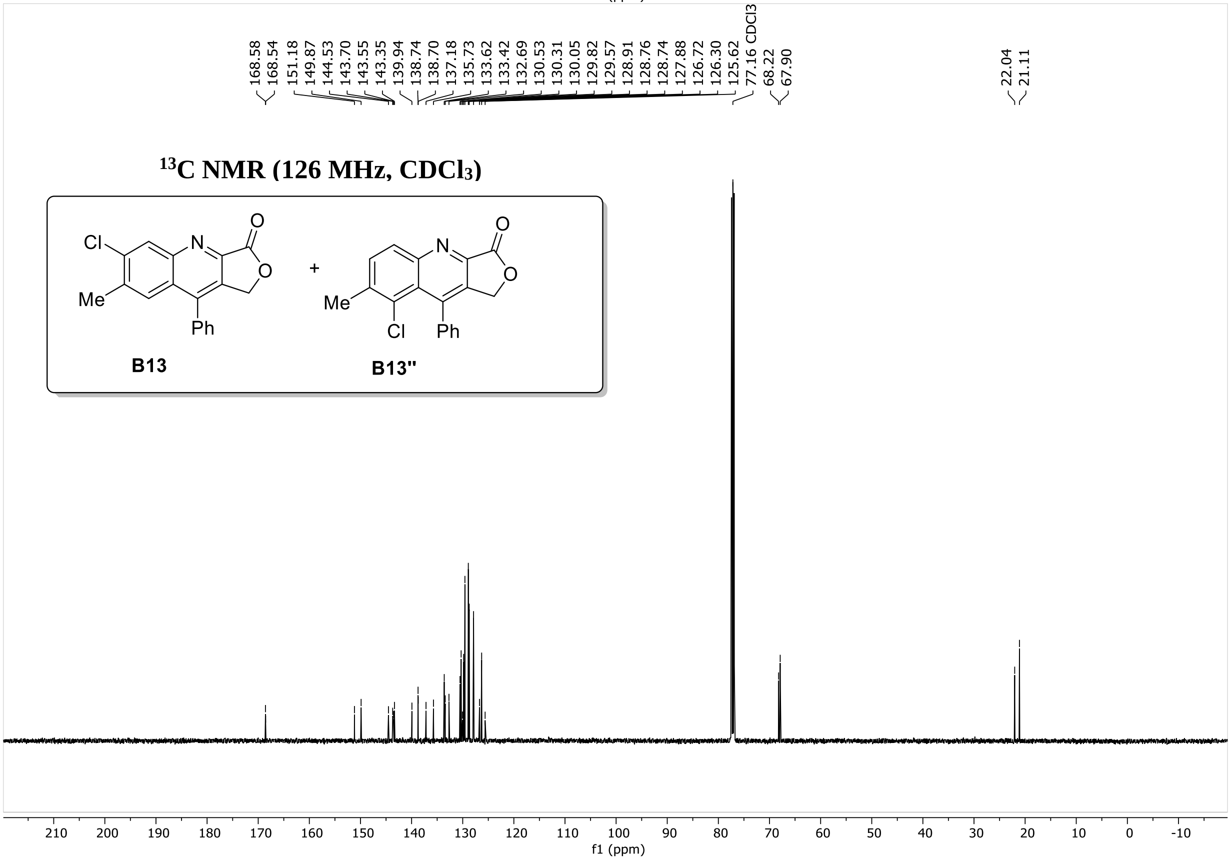

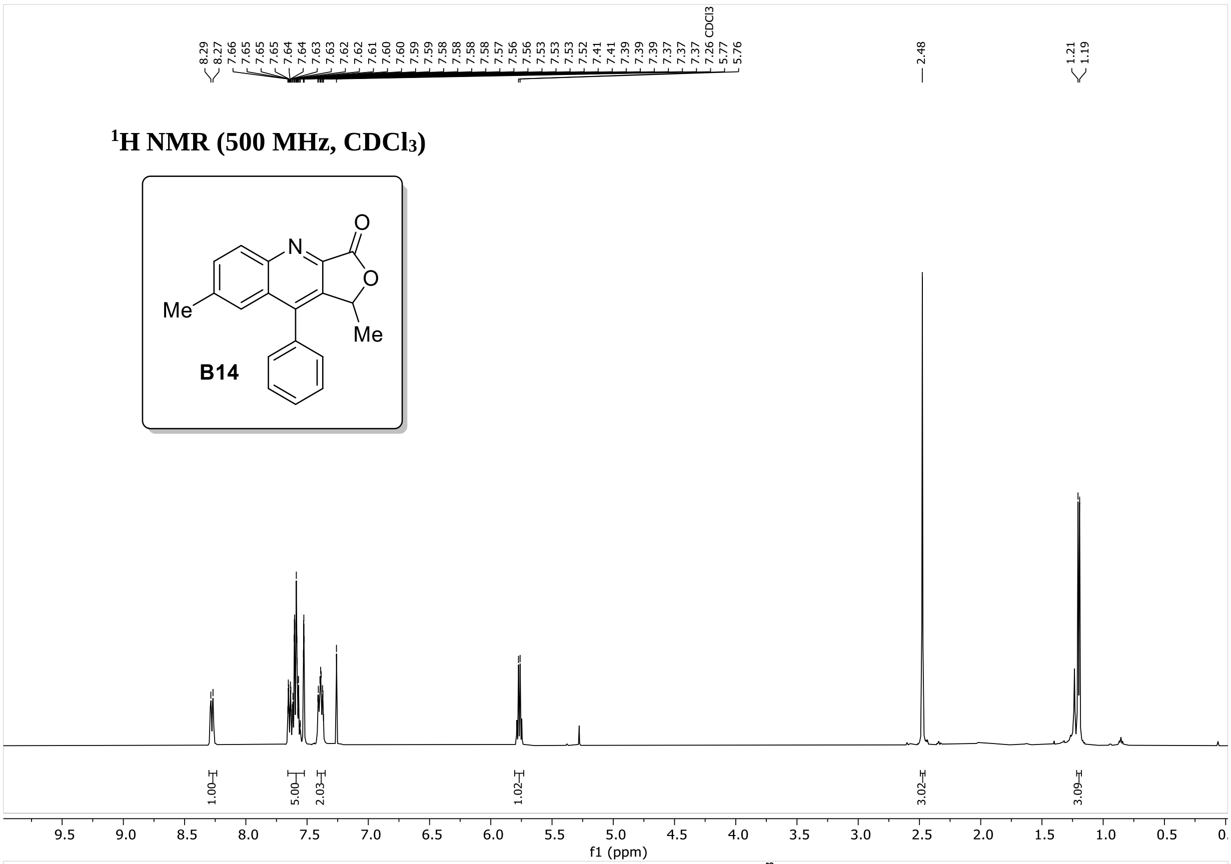
1H NMR (500 MHz, CDCl3)
B14
Me
Me
N
O
O
8.29
8.27
5.77
5.76
2.48
1.21
1.19
CDCl3
1.00
5.00
2.03
1.02
3.02
3.09
f1 (ppm)

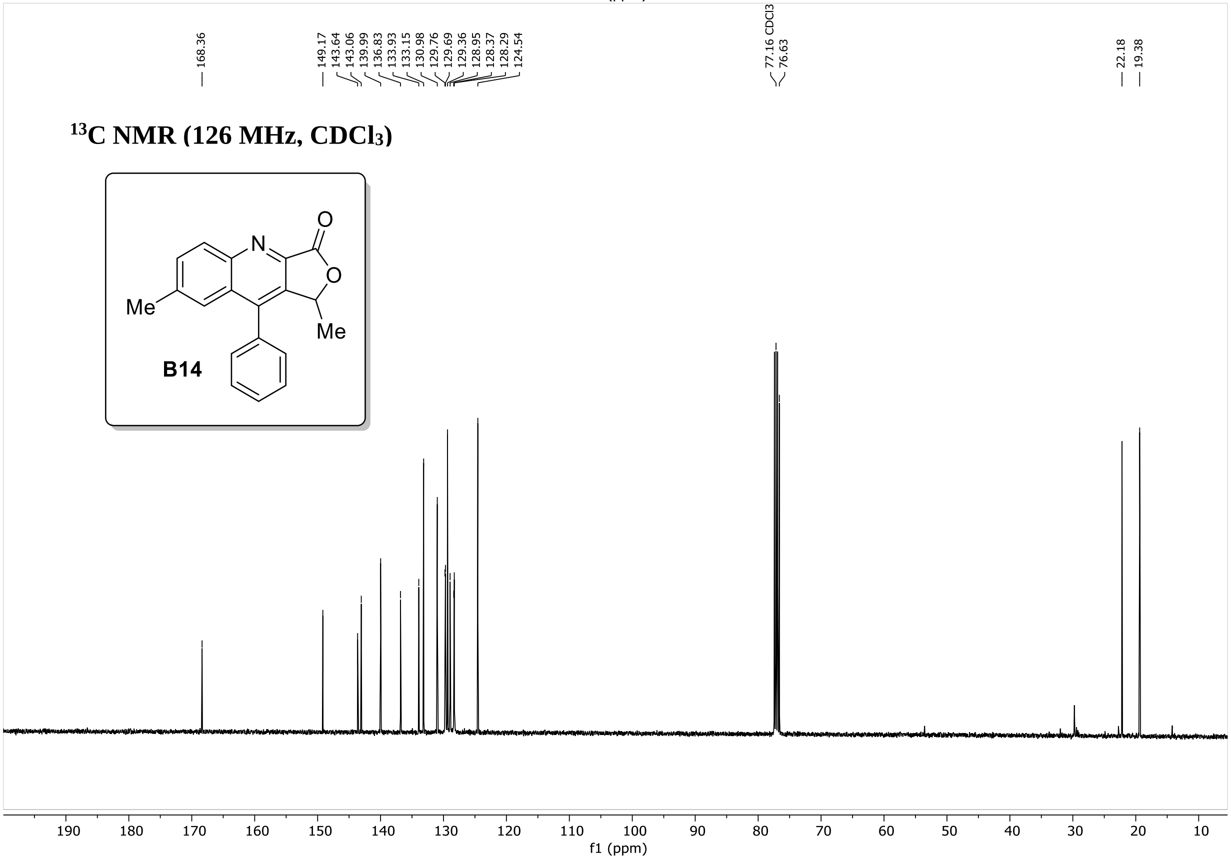
13C NMR (126 MHz, CDCl3)
B14
Me
Me
N
O
O
168.36
149.17
143.64
143.06
139.99
136.83
133.93
133.15
130.98
129.76
129.69
129.36
128.95
128.37
128.29
124.54
77.16 CDCl3
76.63
22.18
19.38
f1 (ppm)

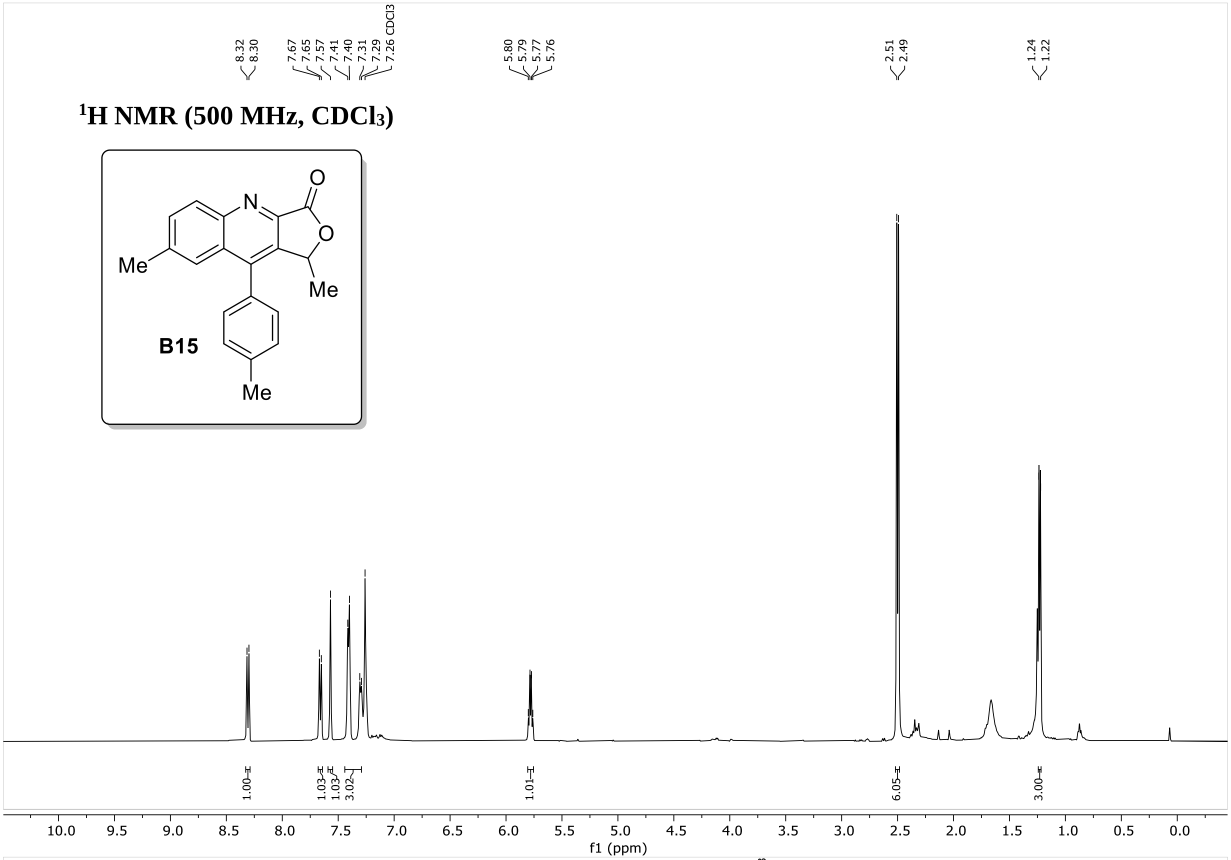

8.32
8.30
7.67
7.65
7.57
7.41
7.40
7.31
7.29
7.26 CDCl3
5.80
5.79
5.77
5.76
2.51
2.49
1.24
1.22
¹H NMR (500 MHz, CDCl₃)
N
O
O
Me
Me
B15
Me
1.00
1.03
1.03
3.02
1.01
6.05
3.00
10.0
9.5
9.0
8.5
8.0
7.5
7.0
6.5
6.0
5.5
5.0
4.5
4.0
3.5
3.0
2.5
2.0
1.5
1.0
0.5
0.0
f1 (ppm)

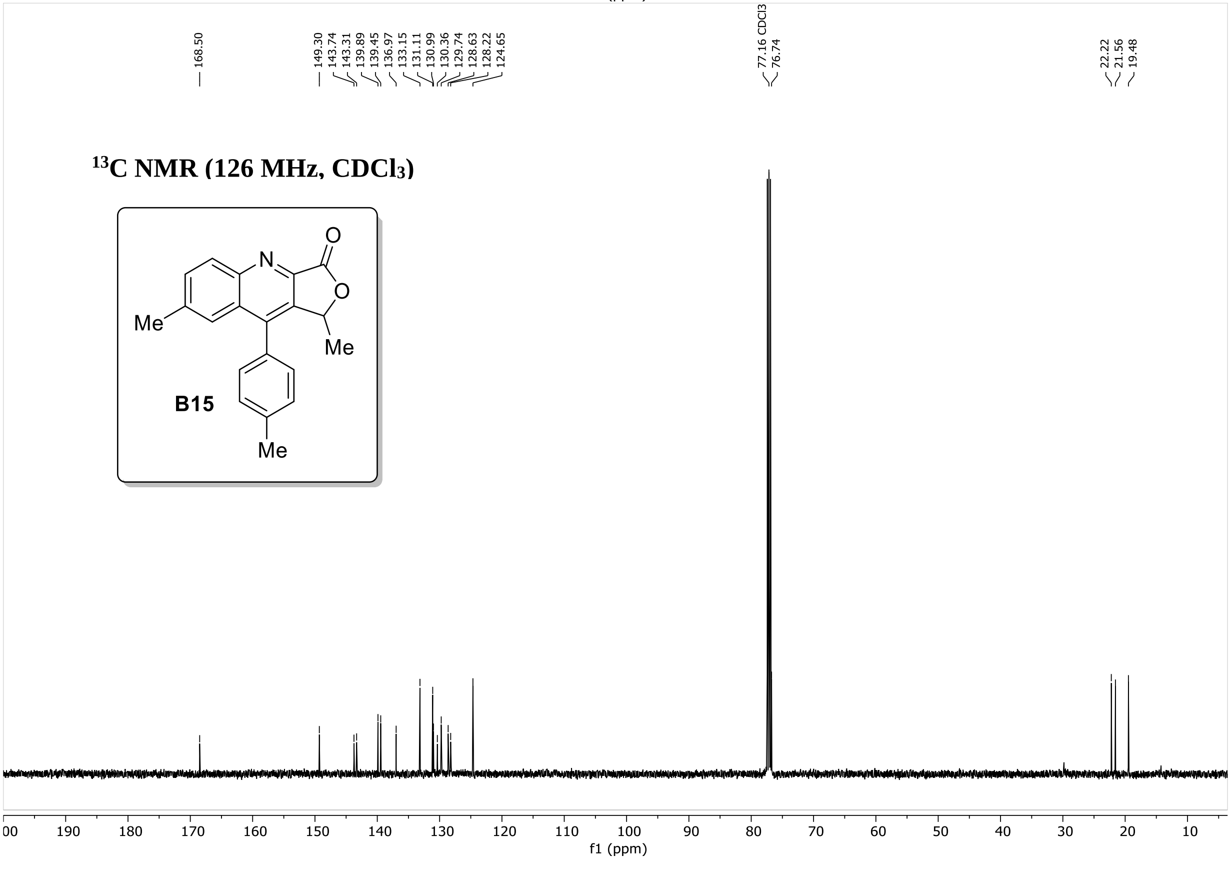

168.50
149.30
143.74
143.31
139.89
139.45
136.97
133.15
131.11
130.99
130.36
129.74
128.63
128.22
124.65
77.16 CDCl3
76.74
22.22
21.56
19.48
¹³C NMR (126 MHz, CDCl₃)
N
O
O
Me
Me
B15
Me
00
190
180
170
160
150
140
130
120
110
100
90
80
70
60
50
40
30
20
10
f1 (ppm)

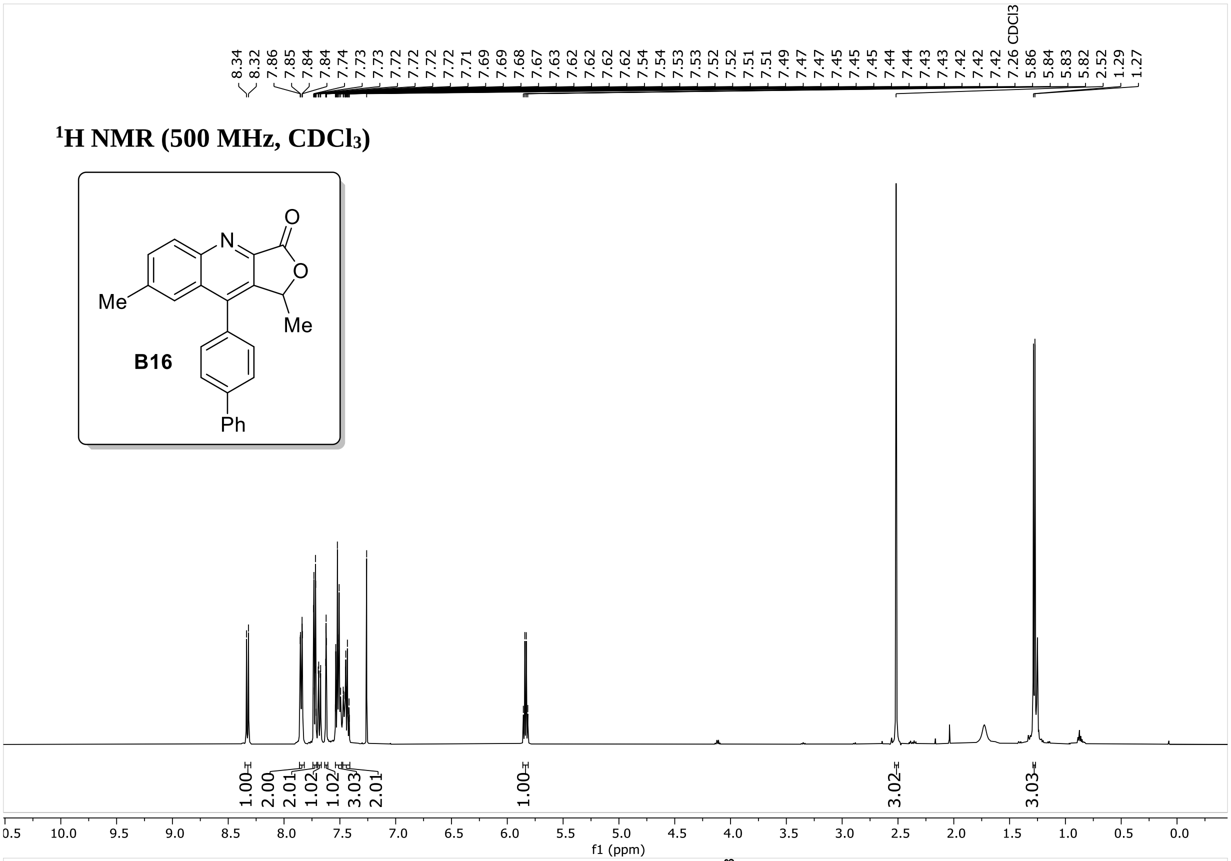

8.34
8.32
7.86
7.85
7.84
7.84
7.74
7.73
7.73
7.72
7.72
7.72
7.72
7.71
7.69
7.68
7.67
7.63
7.62
7.62
7.62
7.62
7.54
7.54
7.53
7.53
7.52
7.52
7.51
7.51
7.49
7.47
7.47
7.45
7.45
7.45
7.44
7.44
7.43
7.43
7.42
7.42
7.42
7.26 CDCl3
5.86
5.84
5.83
5.82
2.52
1.29
1.27
^{1}H NMR (500 MHz, CDCl$_3$)
O
N
O
Me
Me
B16
Ph
1.00
2.00
2.01
1.02
1.02
3.03
2.01
1.00
3.02
3.03
f1 (ppm)

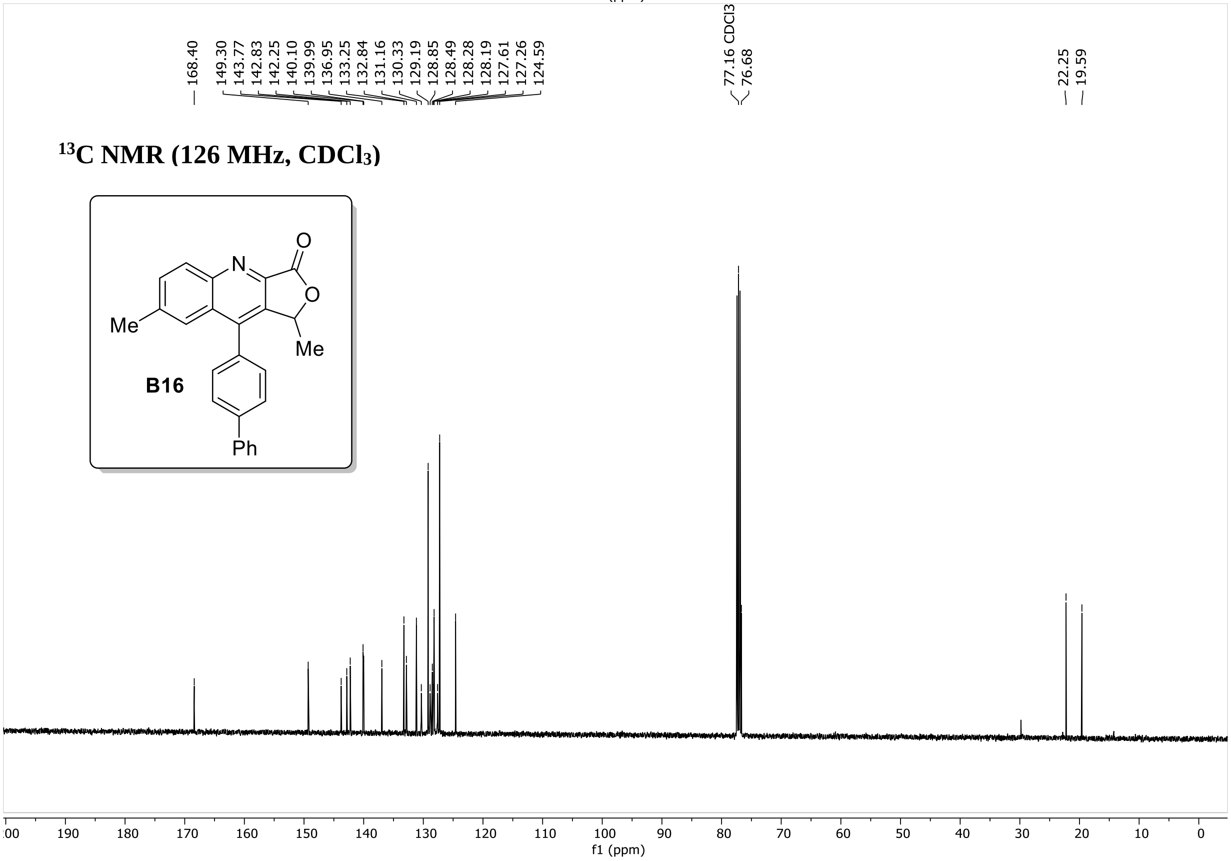

168.40
149.30
143.77
142.83
142.25
140.10
139.99
136.95
133.25
132.84
131.16
130.33
129.19
128.85
128.49
128.28
128.19
127.61
127.26
124.59
77.16 CDCl3
76.68
22.25
19.59
^{13}C NMR (126 MHz, CDCl$_3$)
O
N
O
Me
Me
B16
Ph
f1 (ppm)

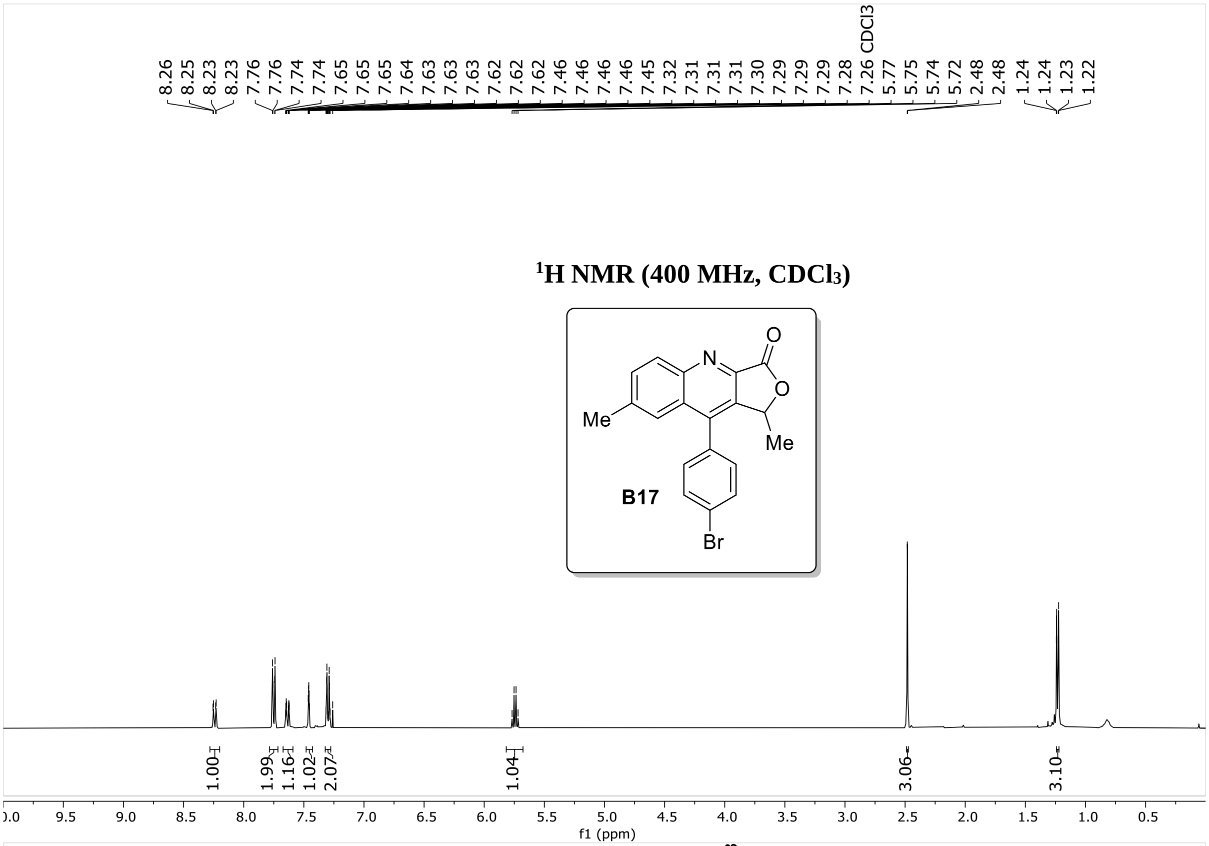
8.26 8.25 8.23 8.23 7.76 7.76 7.74 7.74 7.65 7.65 7.65 7.64 7.63 7.63 7.63 7.62 7.62 7.62 7.46 7.46 7.46 7.46 7.45 7.32 7.31 7.31 7.31 7.30 7.29 7.29 7.29 7.28 7.26 CDCl3 5.77 5.75 5.74 5.72 2.48 2.48 1.24 1.24 1.23 1.22
1H NMR (400 MHz, CDCl3)
N
O
Me
Me
B17
Br
1.00 1.99 1.16 1.02 2.07 1.04 3.06 3.10
f1 (ppm)

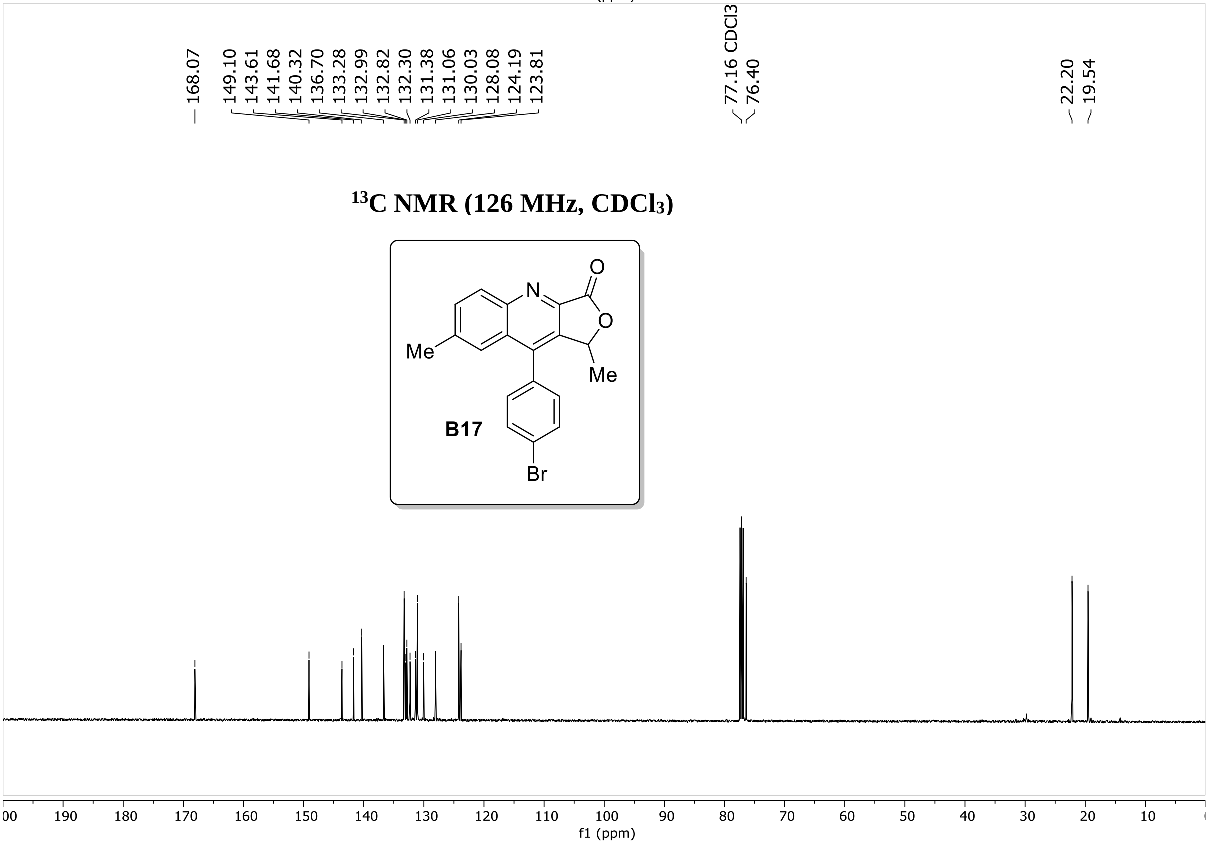
168.07 149.10 143.61 141.68 140.32 136.70 133.28 132.99 132.82 132.30 131.38 131.06 130.03 128.08 124.19 123.81 77.16 CDCl3 76.40 22.20 19.54
13C NMR (126 MHz, CDCl3)
N
O
Me
Me
B17
Br
f1 (ppm)

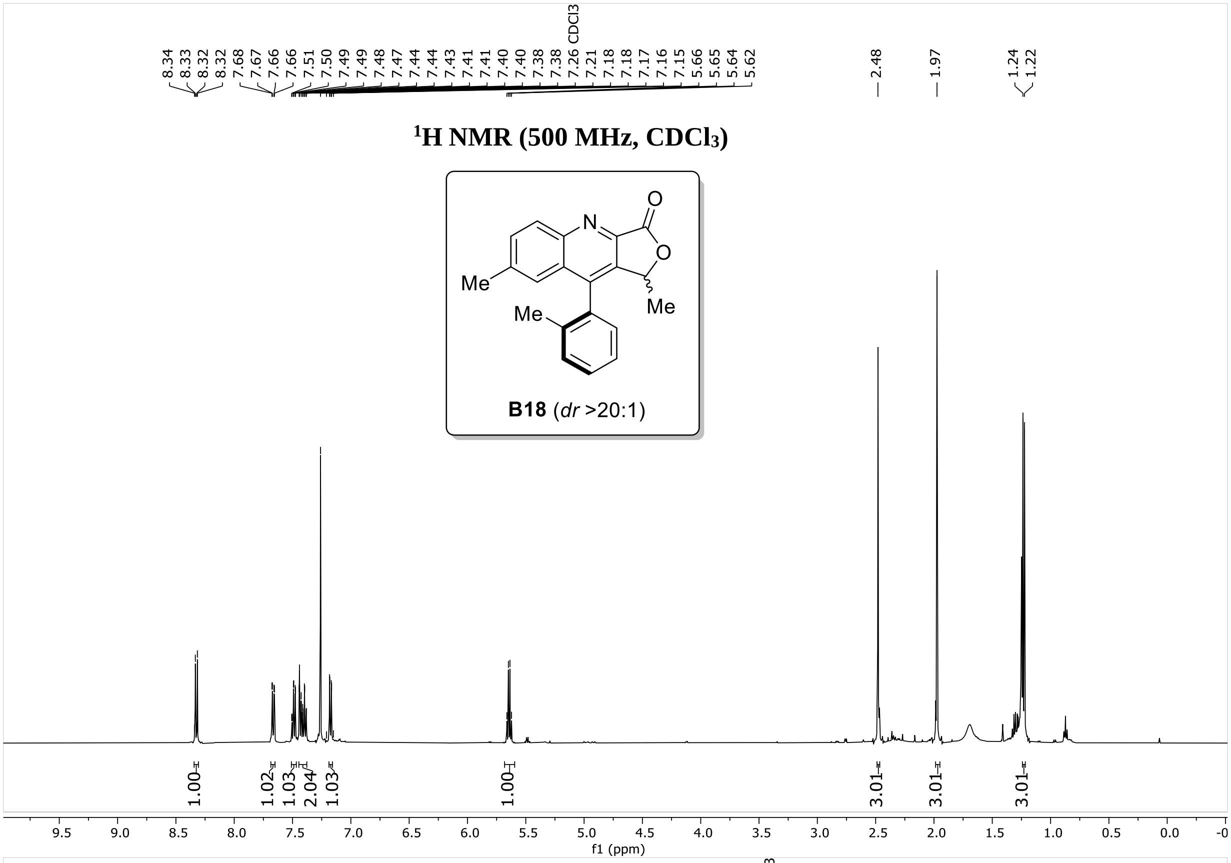

¹H NMR (500 MHz, CDCl₃)
B18 (dr >20:1)
f1 (ppm)

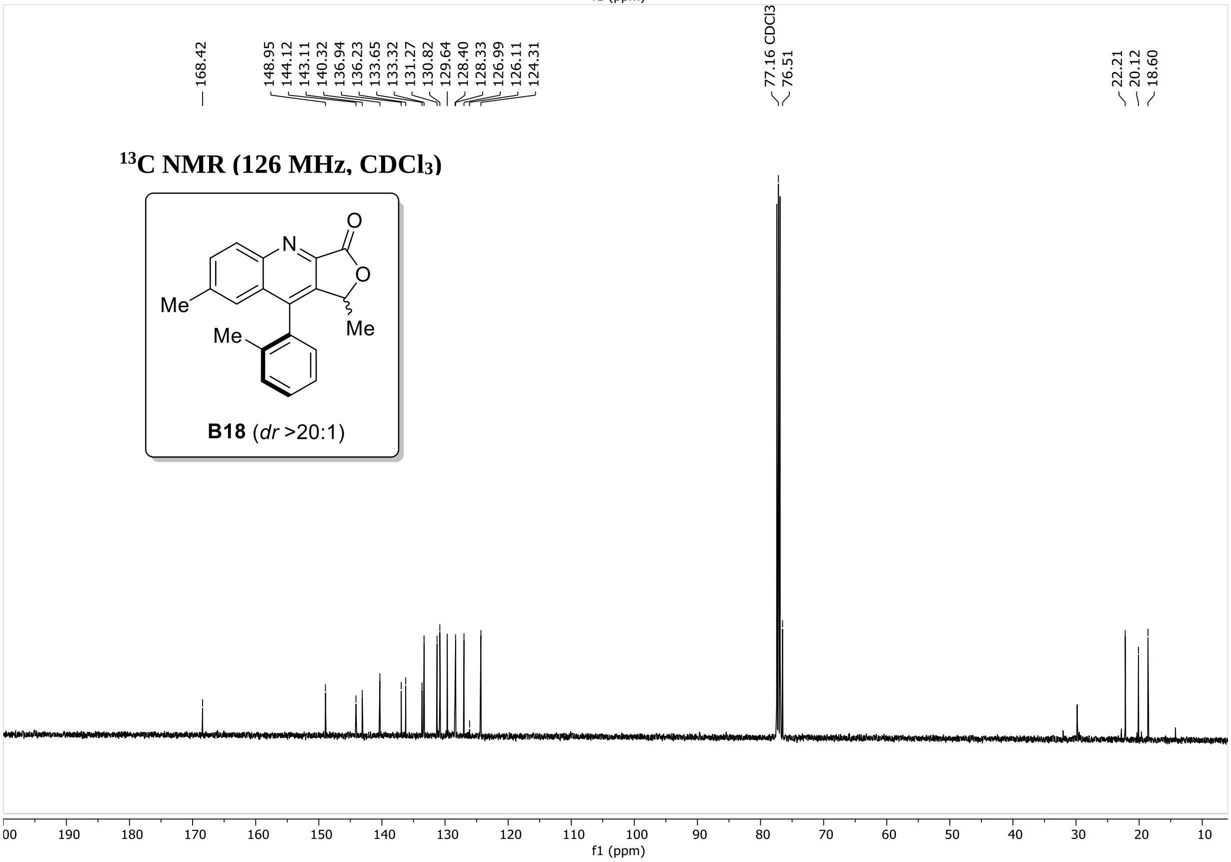

¹³C NMR (126 MHz, CDCl₃)
B18 (dr >20:1)
f1 (ppm)

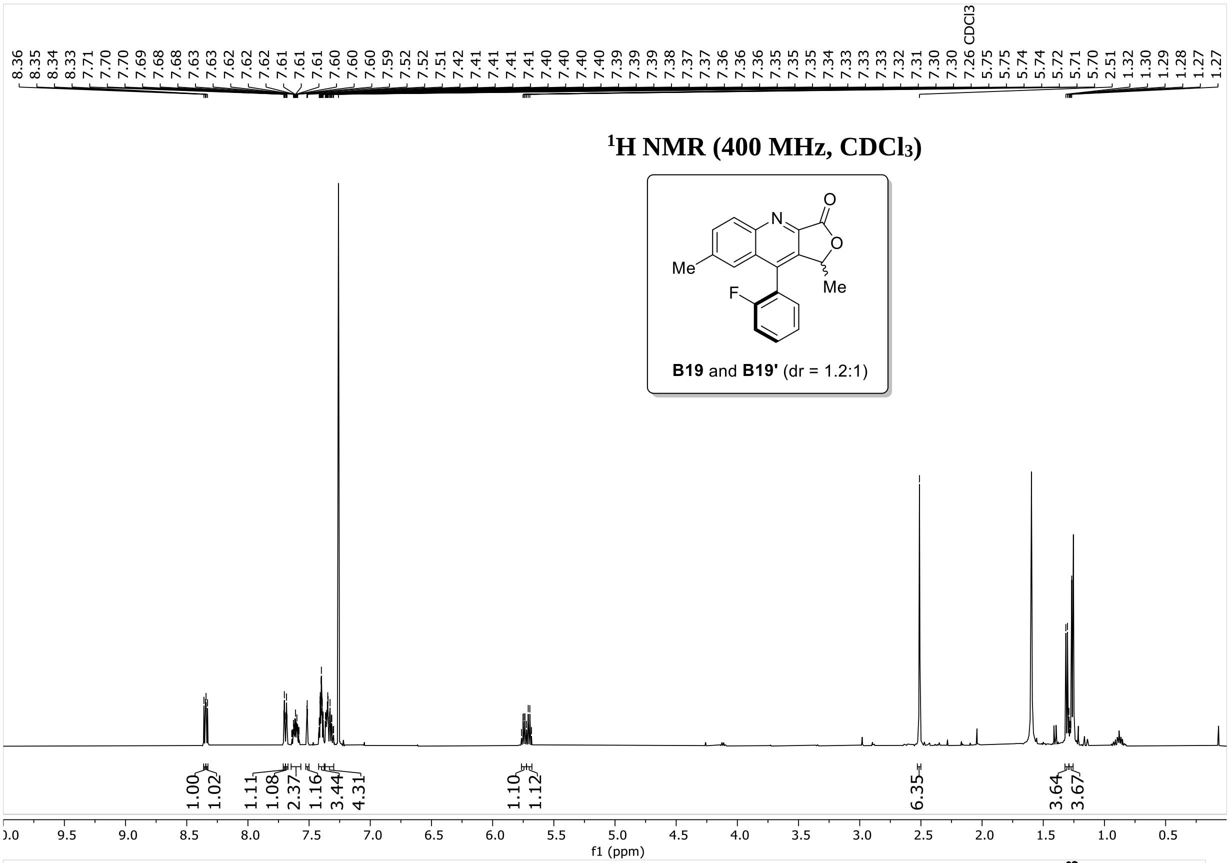

¹H NMR (400 MHz, CDCl₃)
B19 and B19' (dr = 1.2:1)
f1 (ppm)

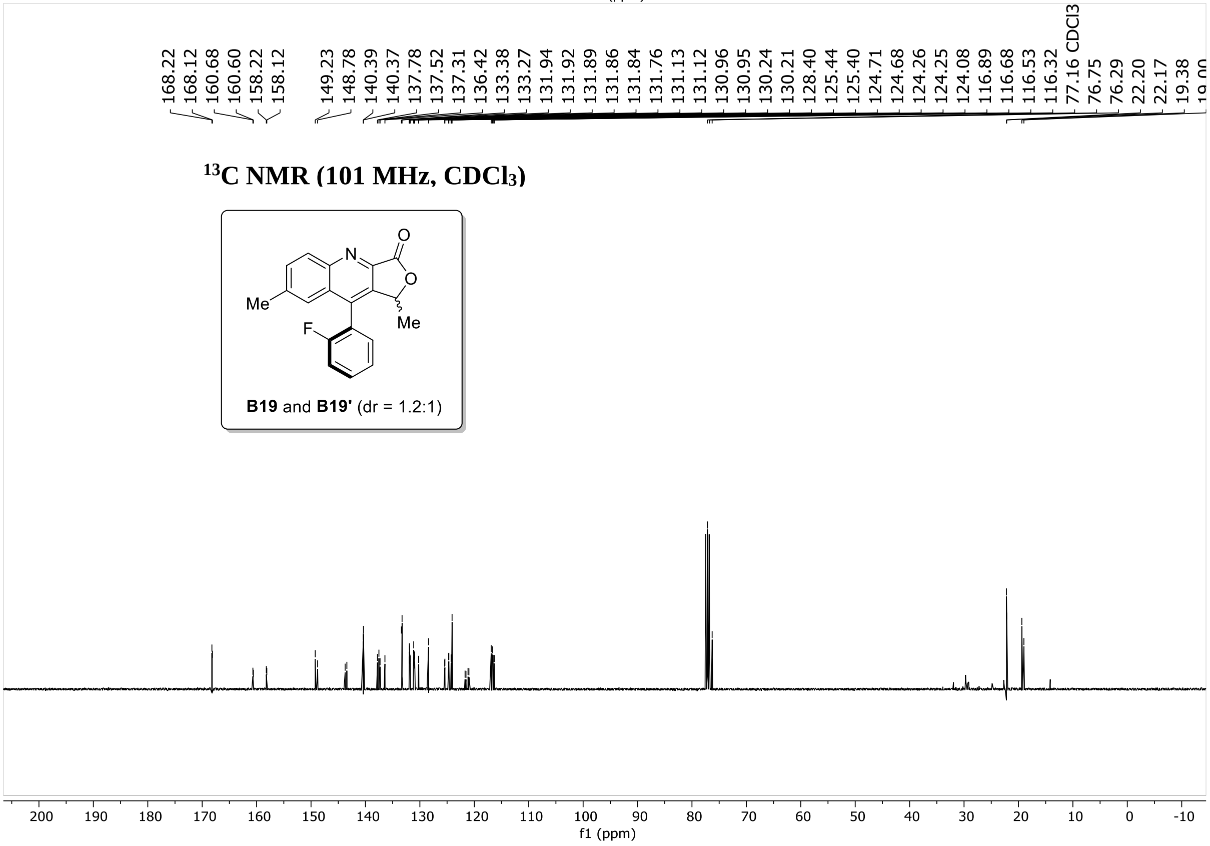

¹³C NMR (101 MHz, CDCl₃)
B19 and B19' (dr = 1.2:1)
f1 (ppm)

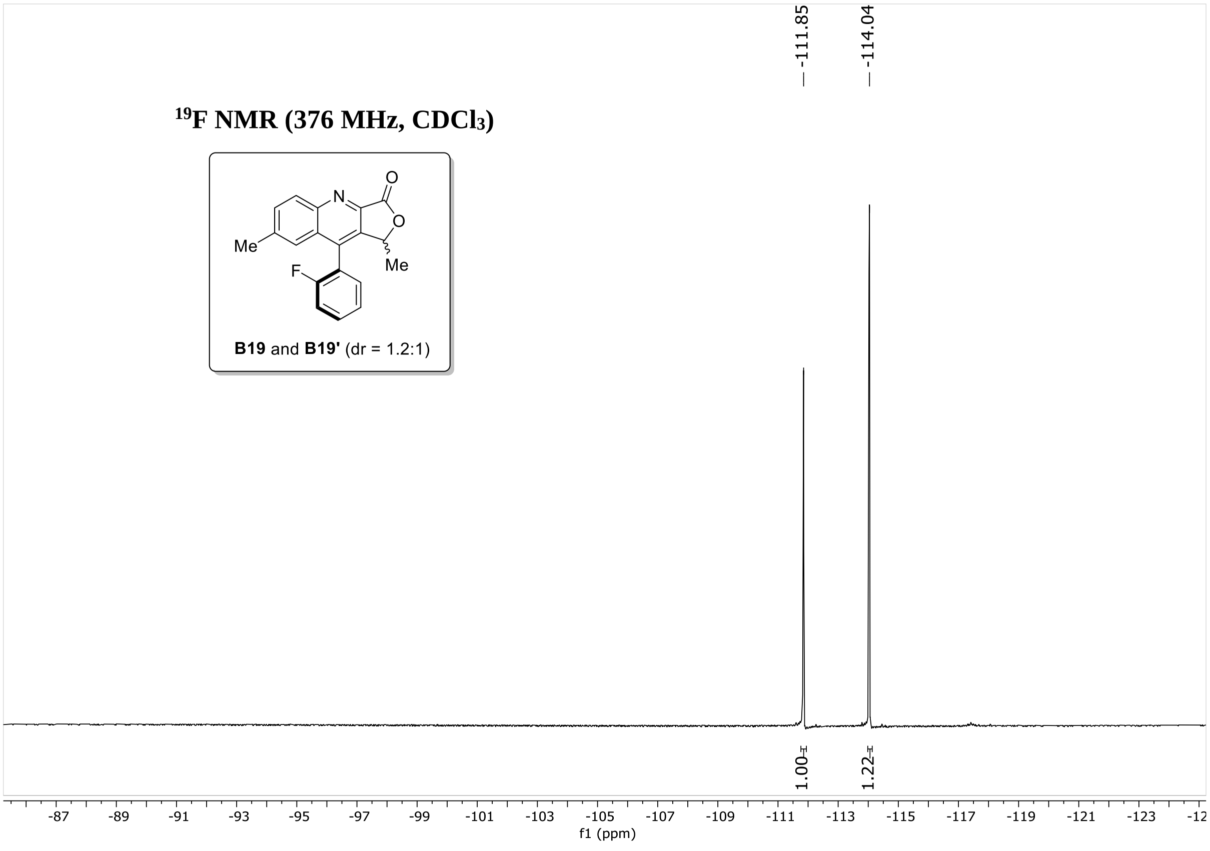

−111.85
−114.04
19F NMR (376 MHz, CDCl3)
N
O
O
Me
Me
F
B19 and B19' (dr = 1.2:1)
1.00
1.22
-87
-89
-91
-93
-95
-97
-99
-101
-103
-105
-107
-109
-111
-113
-115
-117
-119
-121
-123
f1 (ppm)

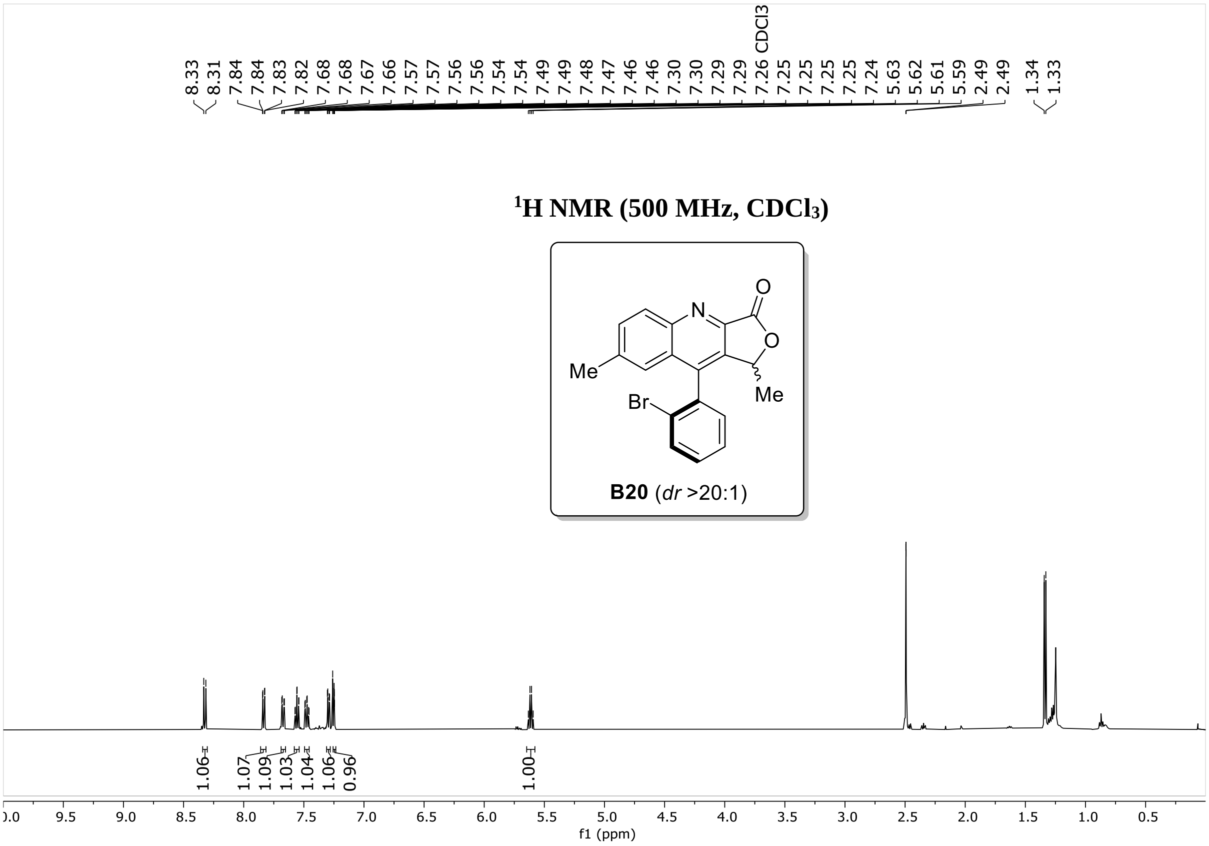
8.33 8.31 7.84 7.84 7.83 7.82 7.68 7.68 7.67 7.66 7.57 7.57 7.56 7.56 7.54 7.54 7.49 7.49 7.48 7.47 7.46 7.30 7.30 7.29 7.29 7.26 CDCl3 7.25 7.25 7.25 7.25 7.24 5.63 5.62 5.61 5.59 2.49 2.49 1.34 1.33
¹H NMR (500 MHz, CDCl₃)
N
O
Me
Br
B20 (dr >20:1)
1.06 1.07 1.09 1.03 1.04 1.06 0.96 1.00
f1 (ppm)

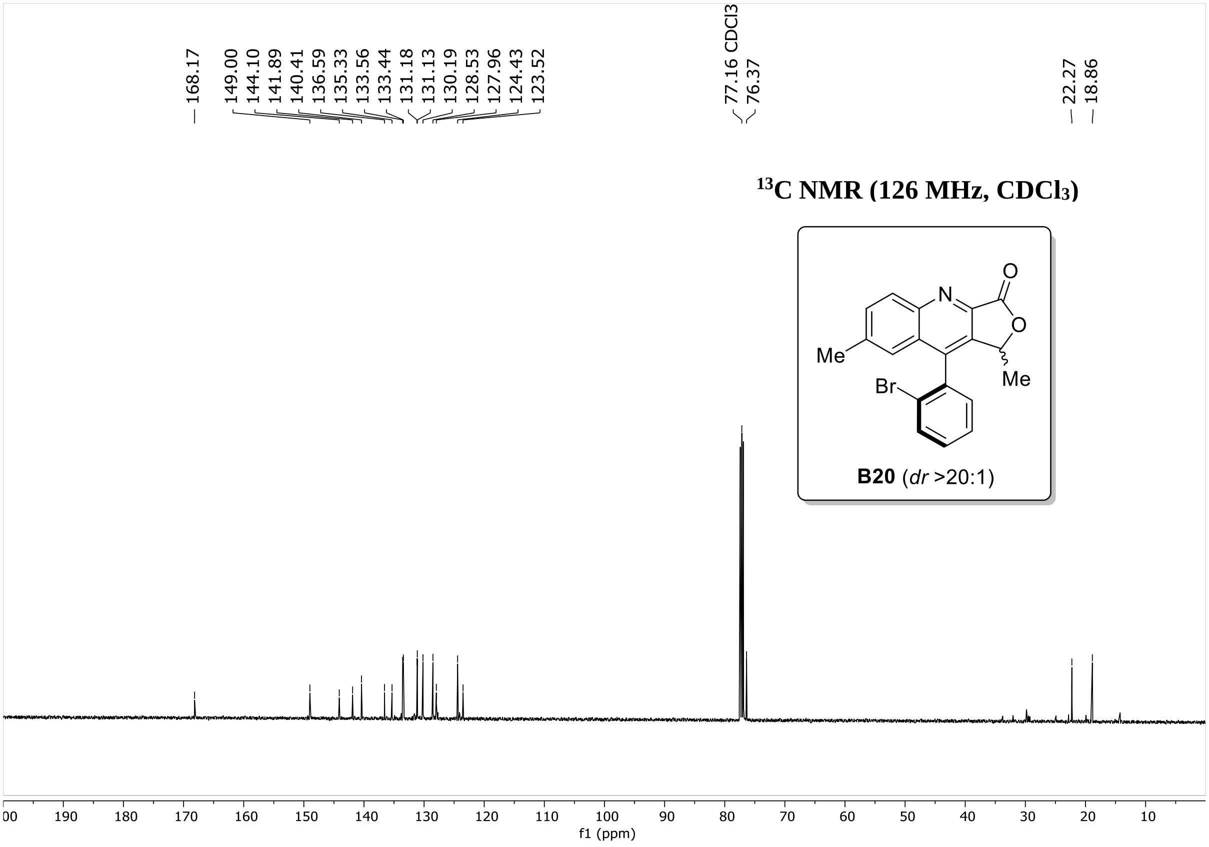
168.17 149.00 144.10 141.89 140.41 136.59 135.33 133.56 133.44 131.18 131.13 130.19 128.53 127.96 124.43 123.52 77.16 CDCl3 76.37 22.27 18.86
¹³C NMR (126 MHz, CDCl₃)
N
O
Me
Br
B20 (dr >20:1)
f1 (ppm)

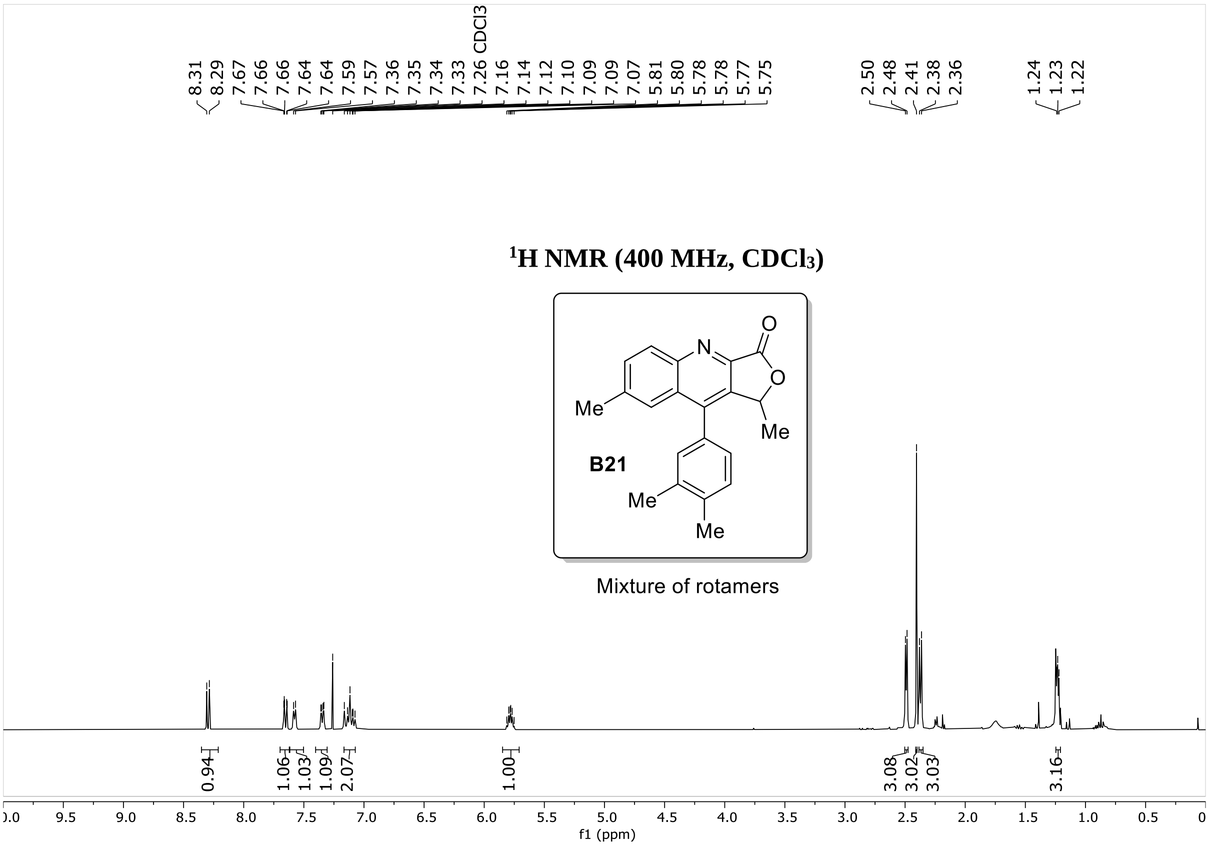

8.31
8.29
7.67
7.66
7.66
7.64
7.64
7.59
7.57
7.36
7.35
7.34
7.33
7.26 CDCl3
7.16
7.14
7.12
7.10
7.09
7.09
7.07
5.81
5.80
5.78
5.78
5.77
5.75
2.50
2.48
2.41
2.38
2.36
1.24
1.23
1.22
1H NMR (400 MHz, CDCl3)
N
O
O
Me
Me
B21
Me
Me
Mixture of rotamers
0.94
1.06
1.03
1.09
2.07
1.00
3.08
3.02
3.03
3.16
f1 (ppm)

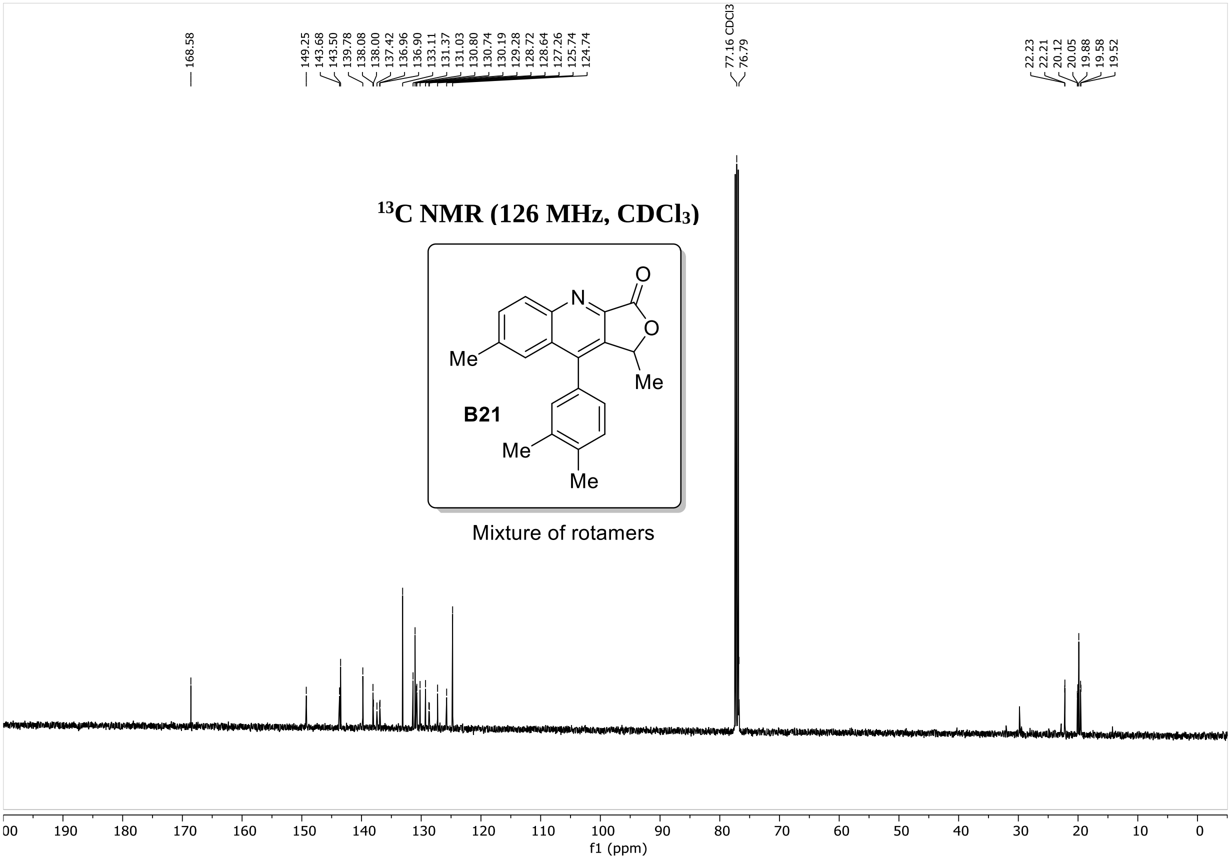

168.58
149.25
143.68
143.50
139.78
138.08
138.00
137.42
136.96
136.90
133.11
131.37
131.03
130.80
130.74
130.19
129.28
128.72
128.64
127.26
125.74
124.74
77.16 CDCl3
76.79
22.23
22.21
20.12
20.05
19.88
19.58
19.52
13C NMR (126 MHz, CDCl3)
N
O
O
Me
Me
B21
Me
Me
Mixture of rotamers
f1 (ppm)

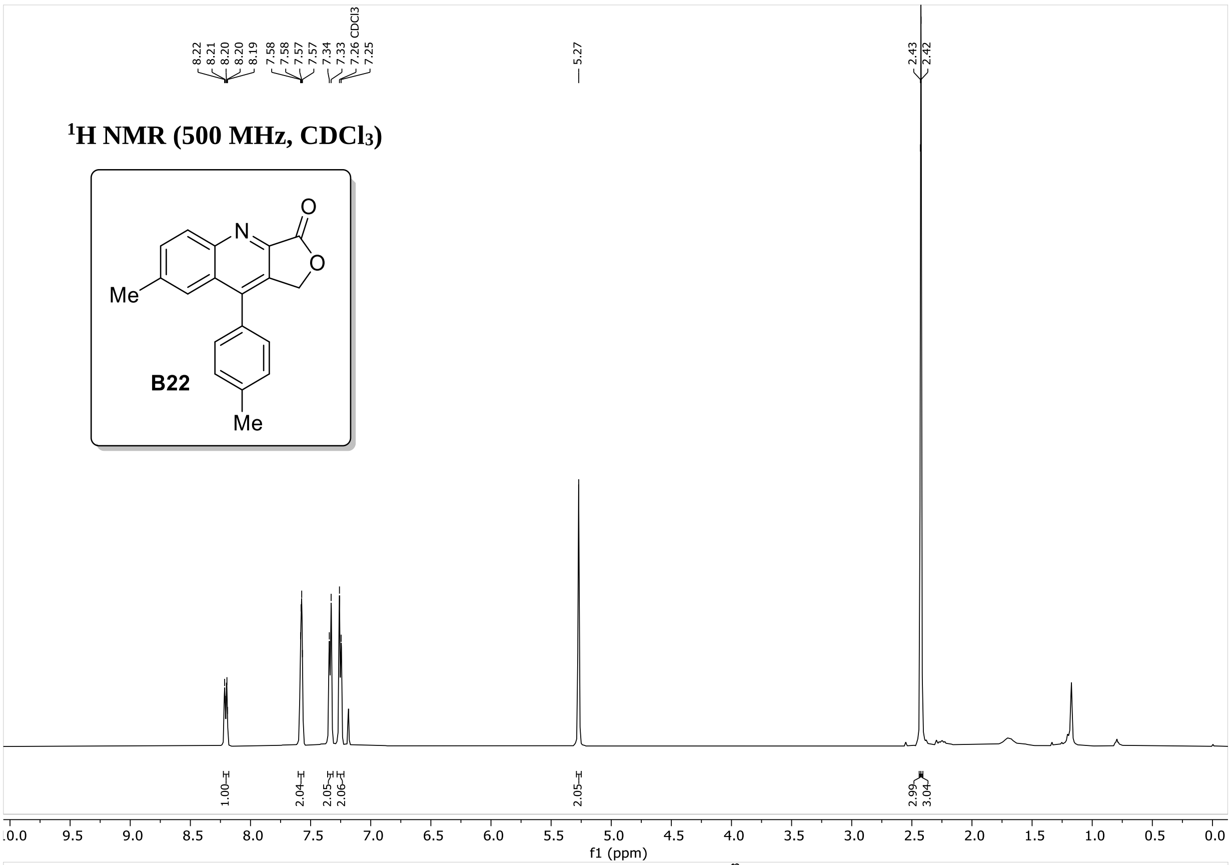
8.22
8.21
8.20
8.20
8.19
7.58
7.58
7.57
7.57
7.34
7.33
7.26 CDCl3
7.25
5.27
2.43
2.42
1H NMR (500 MHz, CDCl3)
Me
N
O
O
B22
Me
1.00
2.04
2.05
2.06
2.05
2.99
3.04
f1 (ppm)

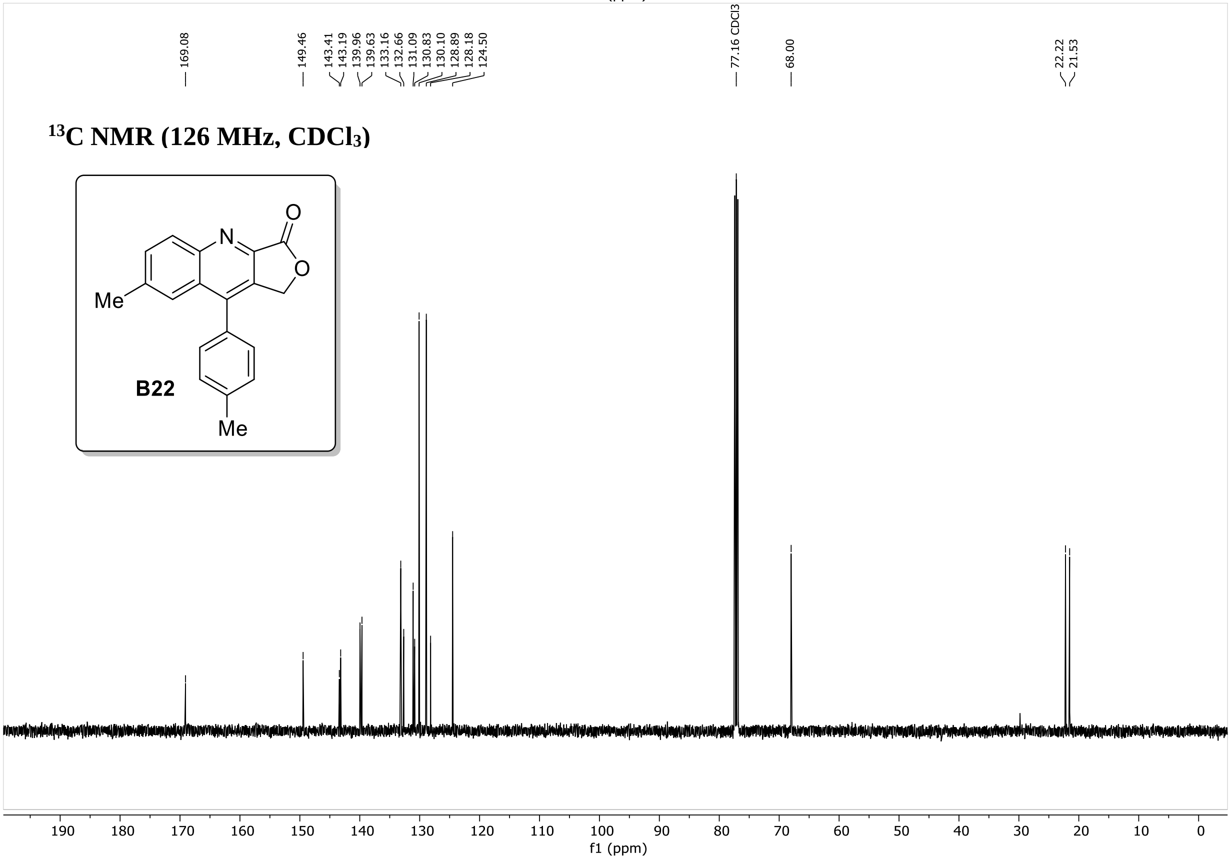
169.08
149.46
143.41
143.19
139.96
139.63
133.16
132.66
131.09
130.83
130.10
128.89
128.18
124.50
77.16 CDCl3
68.00
22.22
21.53
13C NMR (126 MHz, CDCl3)
Me
N
O
O
B22
Me
f1 (ppm)

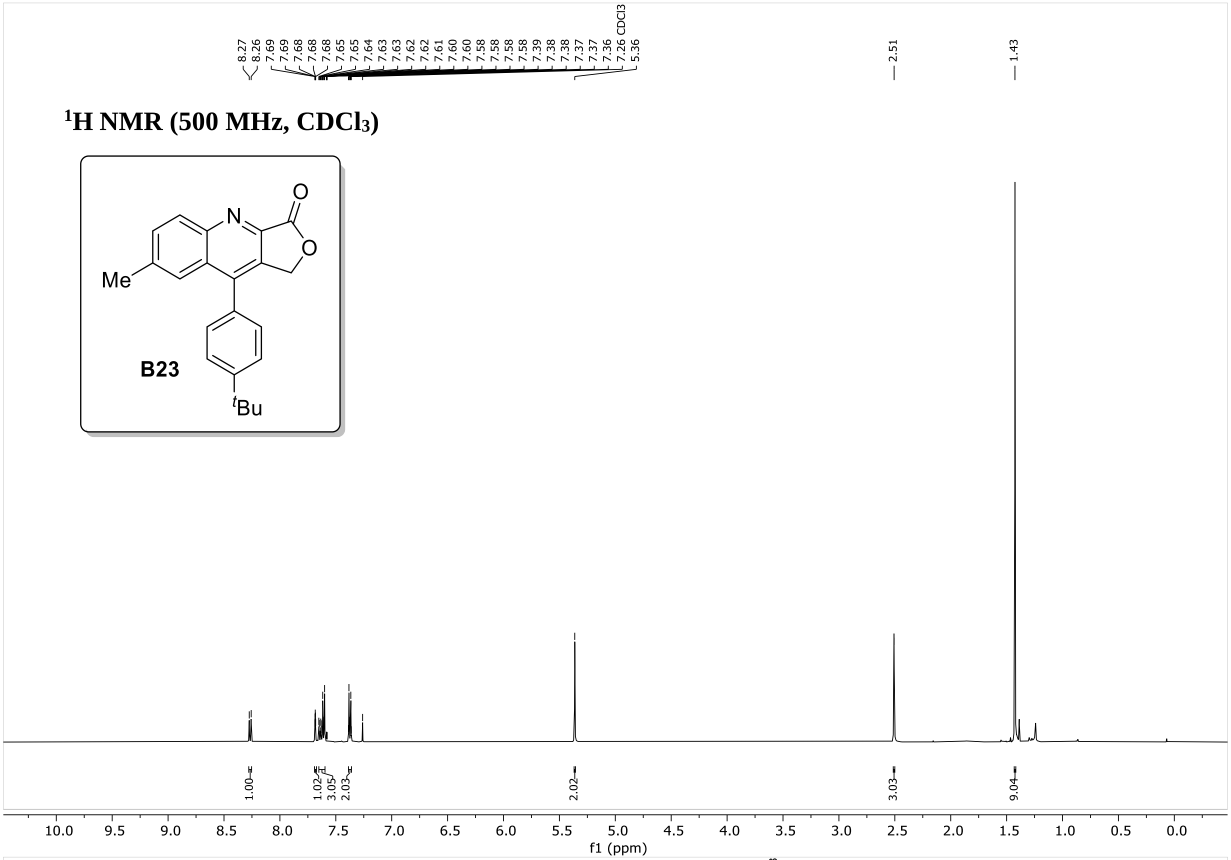

8.27
8.26
7.69
7.69
7.68
7.68
7.68
7.65
7.65
7.64
7.63
7.63
7.62
7.62
7.61
7.60
7.60
7.58
7.58
7.58
7.58
7.39
7.38
7.38
7.37
7.37
7.36
7.26 CDCl3
5.36
2.51
1.43
¹H NMR (500 MHz, CDCl₃)
N
O
O
Me
B23
ᵗBu
1.00
1.02
3.05
2.03
2.02
3.03
9.04
f1 (ppm)

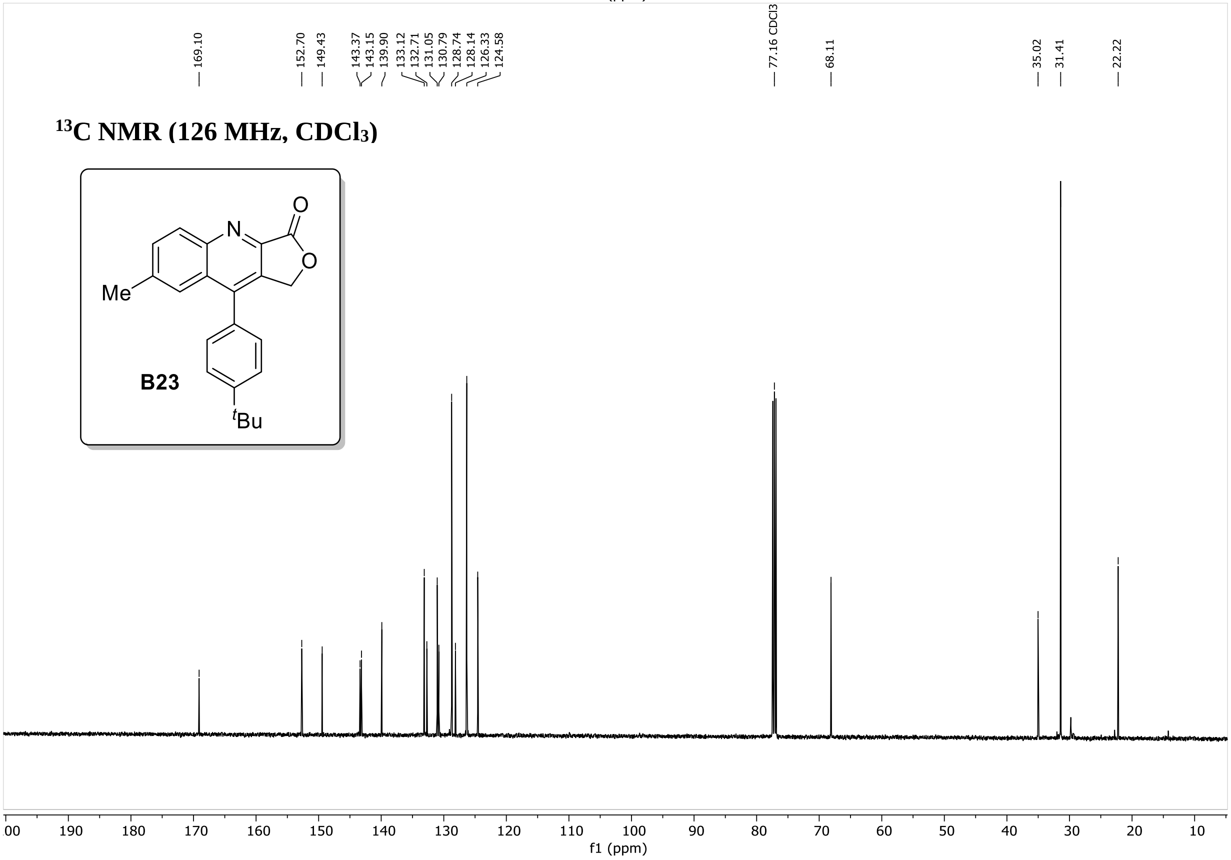

169.10
152.70
149.43
143.37
143.15
139.90
133.12
132.71
131.05
130.79
128.74
128.14
126.33
124.58
77.16 CDCl3
68.11
35.02
31.41
22.22
¹³C NMR (126 MHz, CDCl₃)
N
O
O
Me
B23
ᵗBu
f1 (ppm)

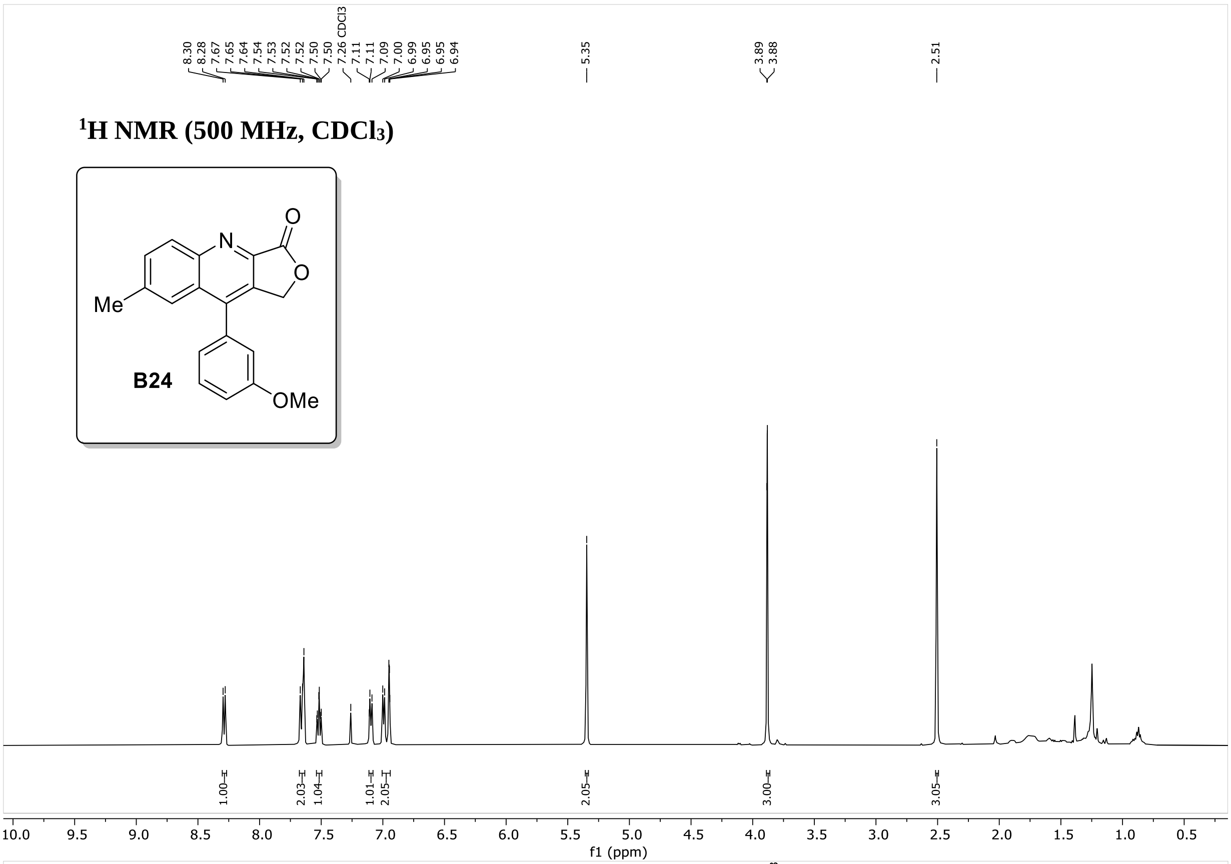
8.30 8.28 7.67 7.65 7.64 7.54 7.53 7.52 7.52 7.50 7.50 7.26 CDCl3 7.11 7.11 7.09 7.00 6.99 6.95 6.95 6.94
5.35
3.89 3.88
2.51
1H NMR (500 MHz, CDCl3)
N
O
O
Me
B24
OMe
1.00 2.03 1.04 1.01 2.05 2.05 3.00 3.05
10.0 9.5 9.0 8.5 8.0 7.5 7.0 6.5 6.0 5.5 5.0 4.5 4.0 3.5 3.0 2.5 2.0 1.5 1.0 0.5
f1 (ppm)

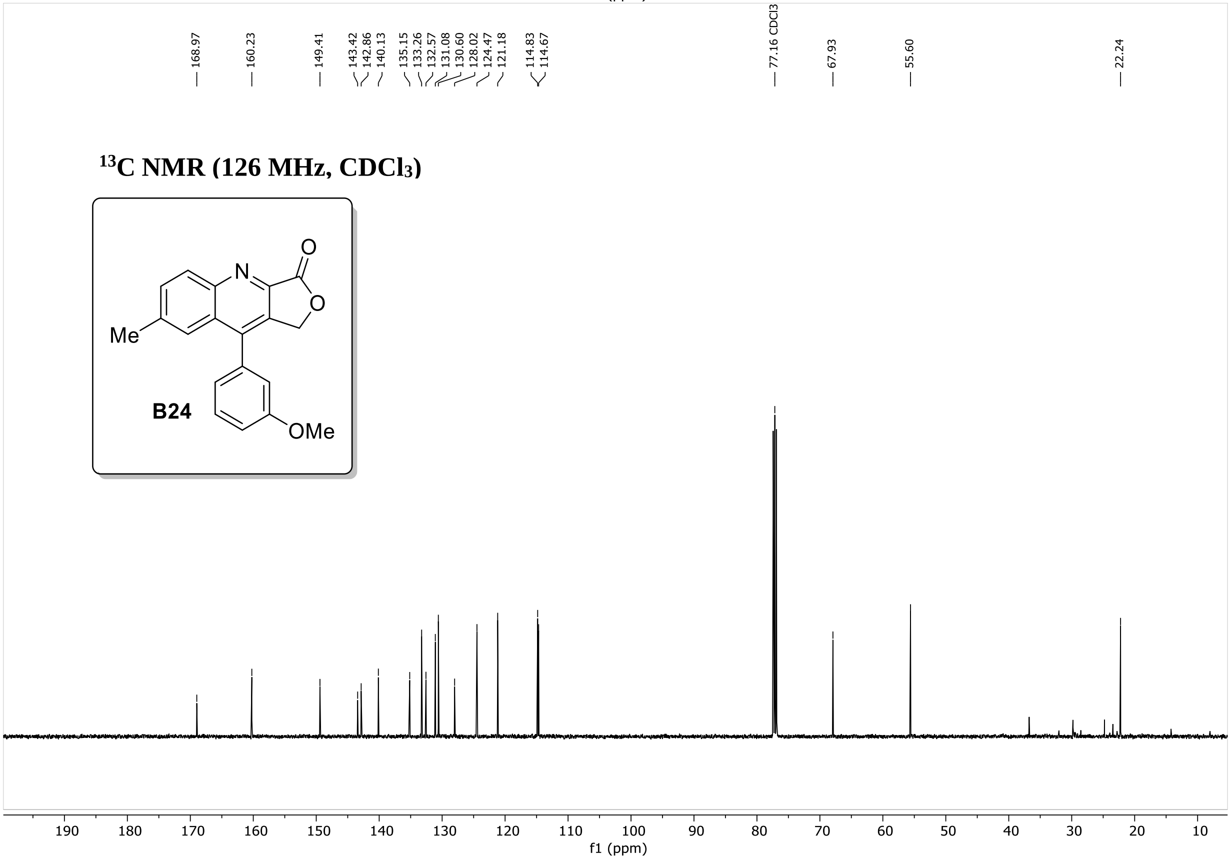
168.97
160.23
149.41
143.42 142.86 140.13
135.15 133.26 132.57 131.08 130.60 128.02 124.47 121.18
114.83 114.67
77.16 CDCl3
67.93
55.60
22.24
13C NMR (126 MHz, CDCl3)
N
O
O
Me
B24
OMe
190 180 170 160 150 140 130 120 110 100 90 80 70 60 50 40 30 20 10
f1 (ppm)

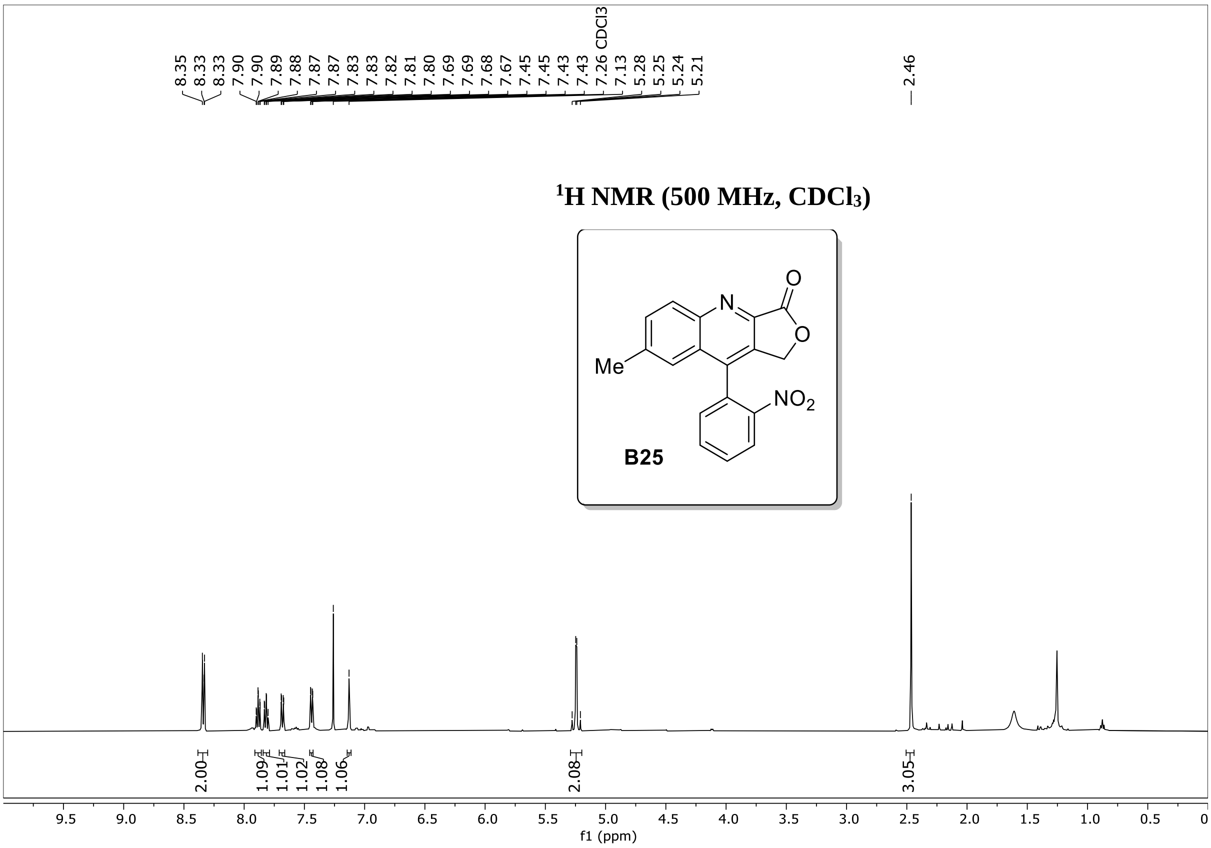
8.35
8.33
8.33
7.90
7.90
7.89
7.88
7.87
7.87
7.83
7.83
7.82
7.81
7.80
7.69
7.69
7.68
7.67
7.45
7.45
7.43
7.43
7.26 CDCl3
7.13
5.28
5.25
5.24
5.21
2.46
1H NMR (500 MHz, CDCl3)
O
N
O
Me
NO2
B25
2.00
1.09
1.01
1.02
1.08
1.06
2.08
3.05
9.5
9.0
8.5
8.0
7.5
7.0
6.5
6.0
5.5
5.0
4.5
4.0
3.5
3.0
2.5
2.0
1.5
1.0
0.5
0
f1 (ppm)

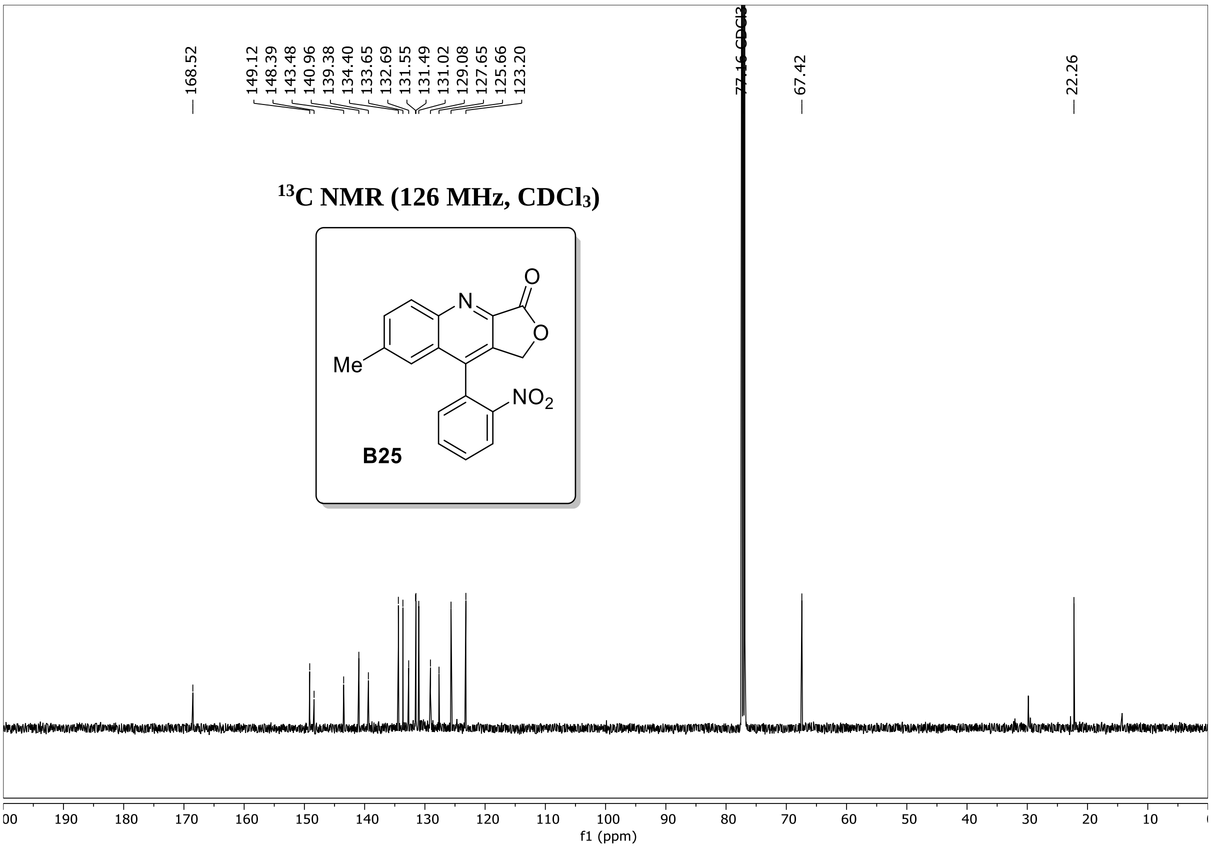
168.52
149.12
148.39
143.48
140.96
139.38
134.40
133.65
132.69
131.55
131.49
131.02
129.08
127.65
125.66
123.20
77.16 CDCl3
67.42
22.26
13C NMR (126 MHz, CDCl3)
O
N
O
Me
NO2
B25
190
180
170
160
150
140
130
120
110
100
90
80
70
60
50
40
30
20
10
f1 (ppm)

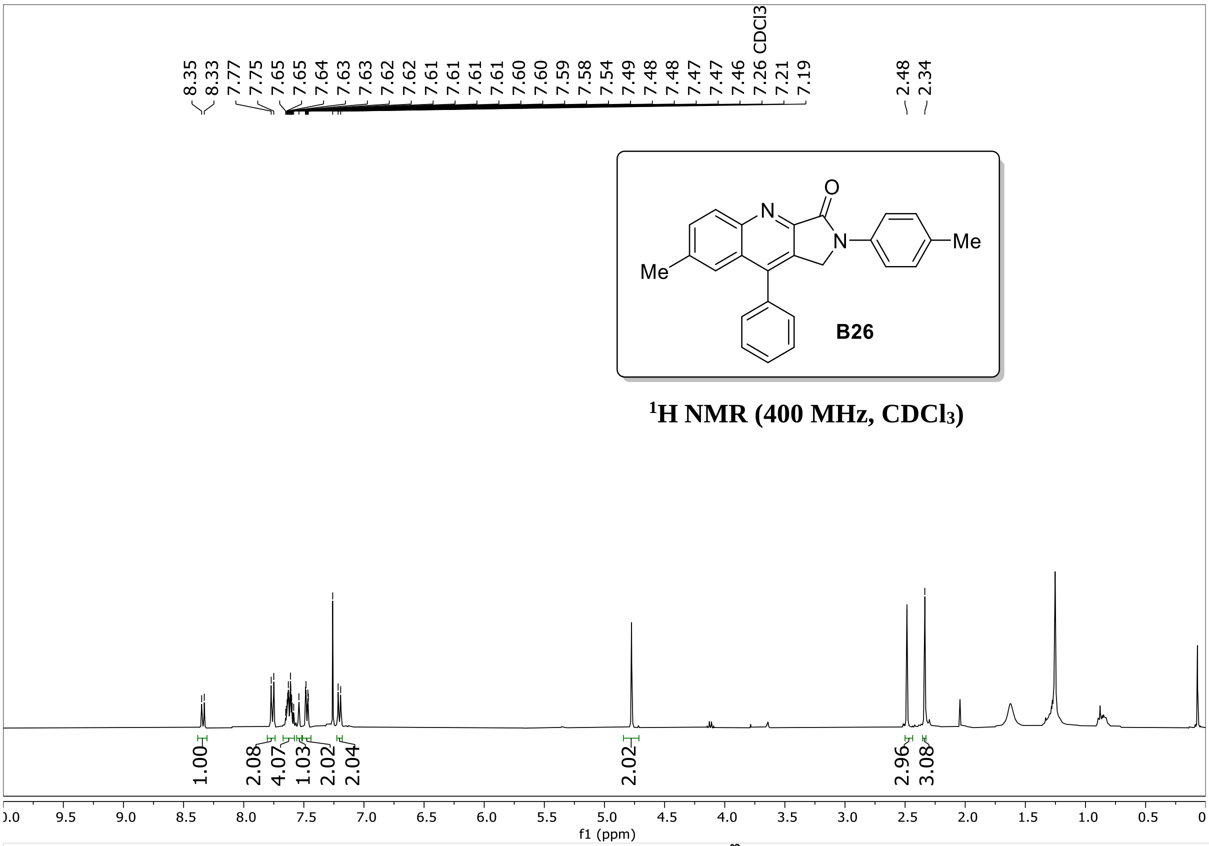

^{1}H NMR (400 MHz, $CDCl_3$)

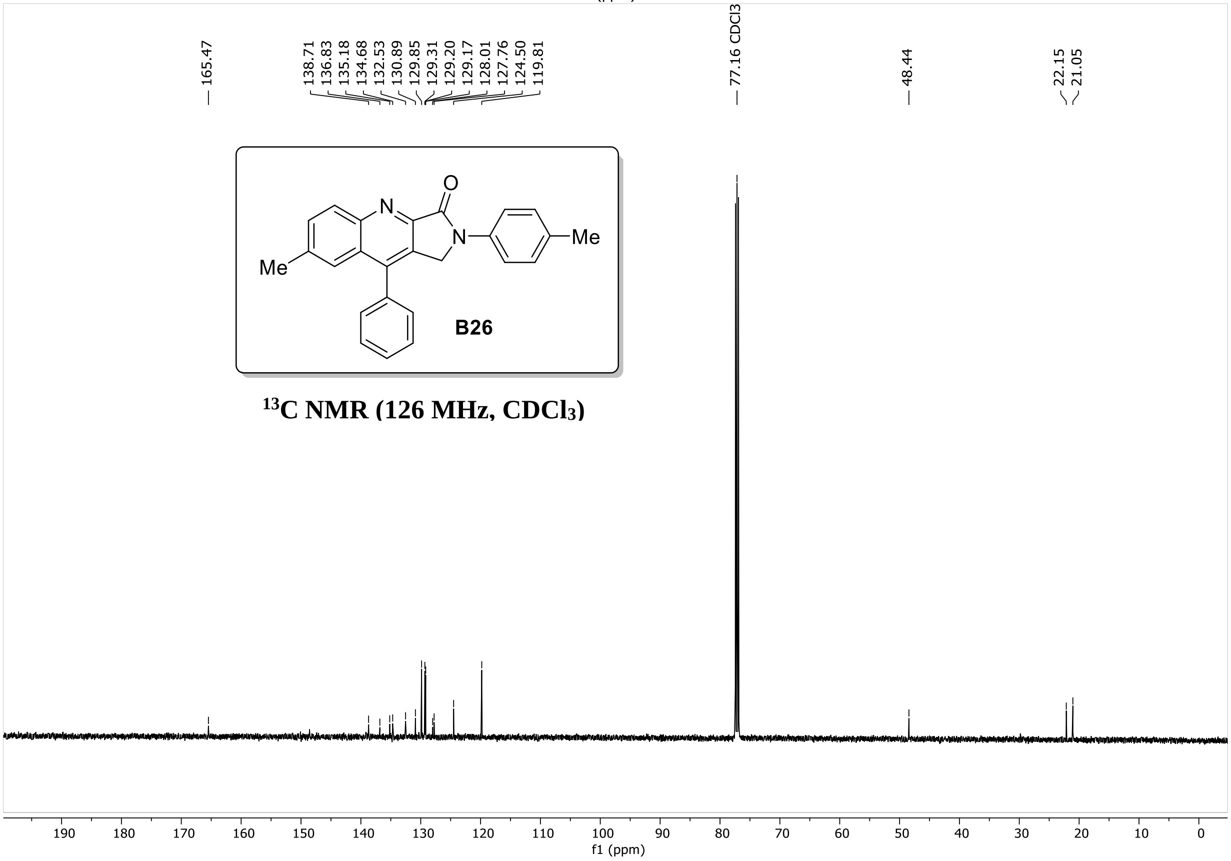

^{13}C NMR (126 MHz, $CDCl_3$)

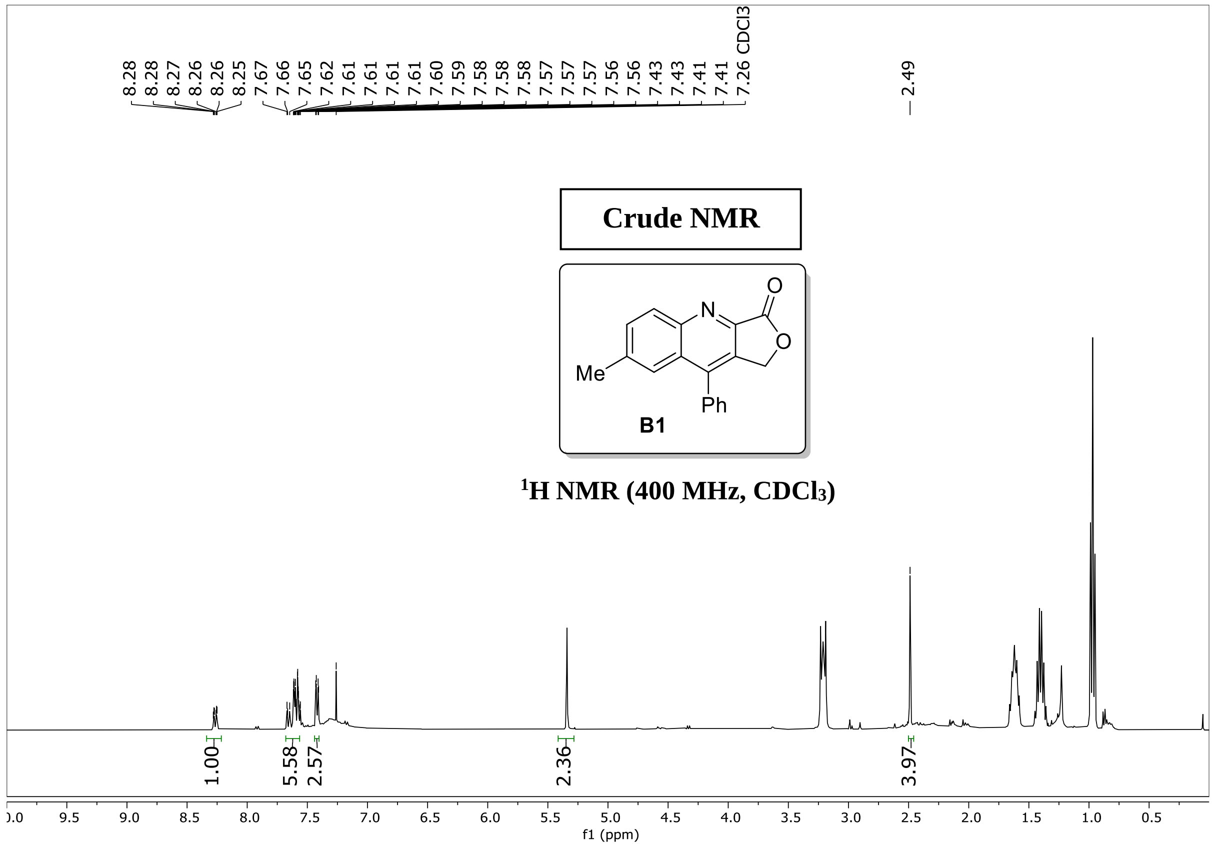
8.28
8.28
8.27
8.26
8.26
8.25
7.67
7.66
7.65
7.62
7.61
7.61
7.61
7.61
7.60
7.59
7.58
7.58
7.58
7.57
7.57
7.57
7.56
7.56
7.43
7.43
7.41
7.41
7.26 CDCl3
2.49
Crude NMR
Me
N
O
O
Ph
B1
^{1}H NMR (400 MHz, CDCl$_3$)
1.00
5.58
2.57
2.36
3.97
f1 (ppm)

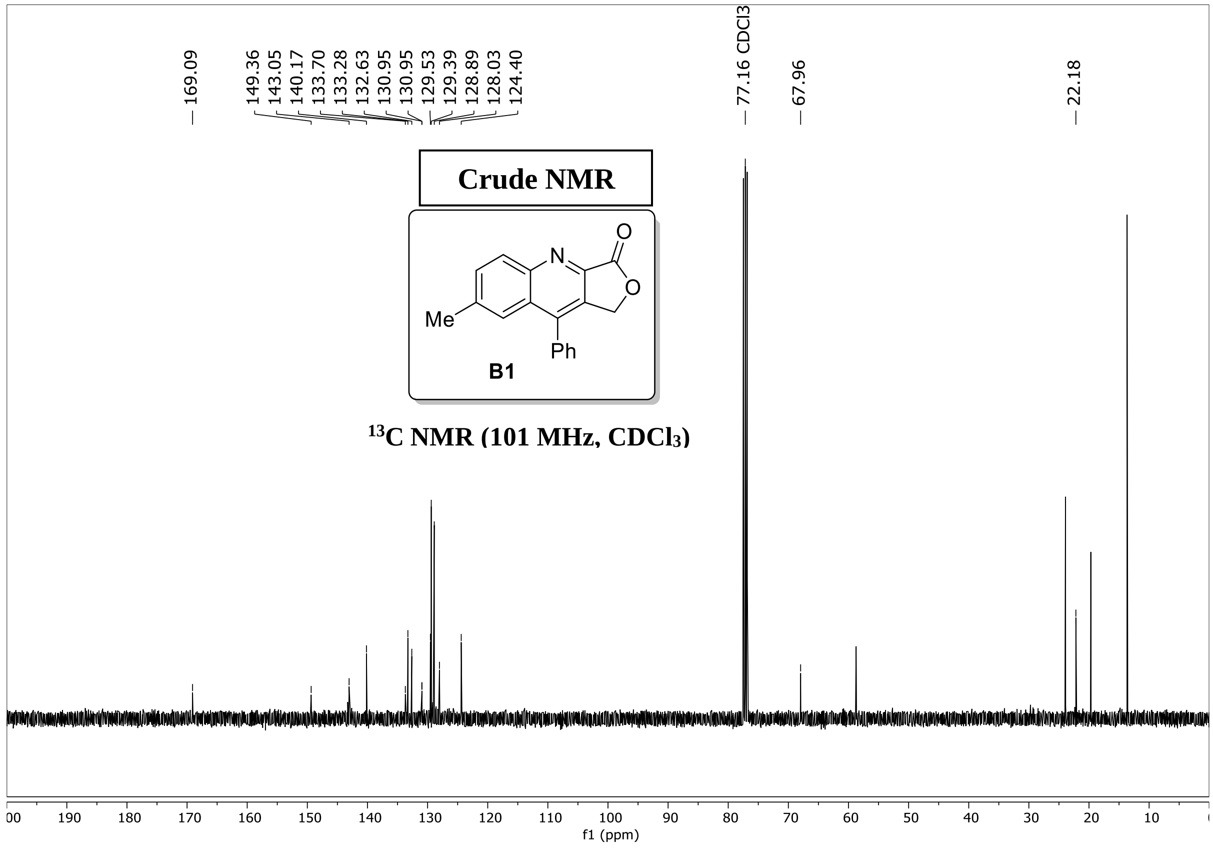
169.09
149.36
143.05
140.17
133.70
133.28
132.63
130.95
130.95
129.53
129.39
128.89
128.03
124.40
77.16 CDCl3
67.96
22.18
Crude NMR
Me
N
O
O
Ph
B1
^{13}C NMR (101 MHz, CDCl$_3$)
f1 (ppm)

12. HRMS Spectra of Intermediate:

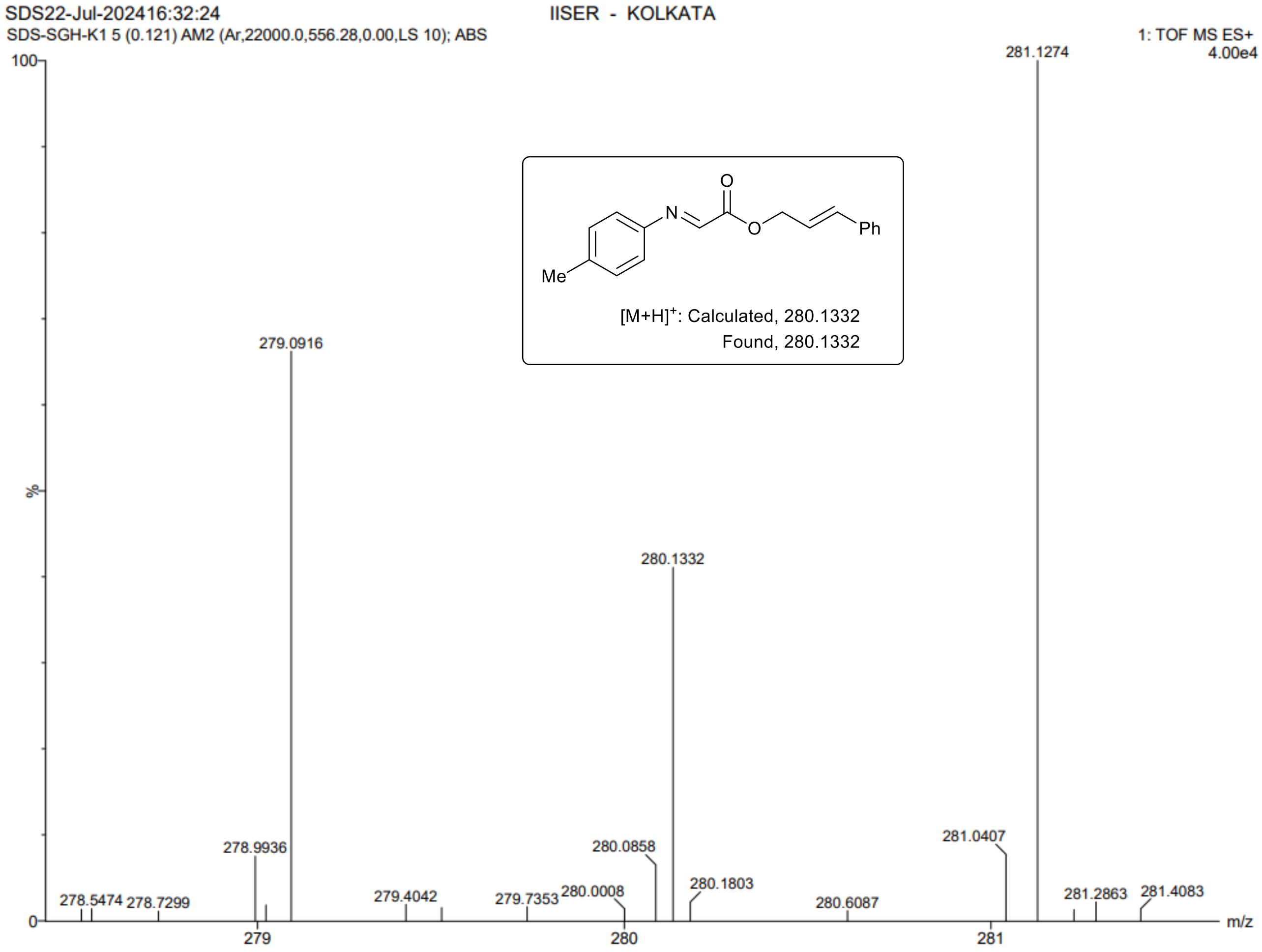

Supplement: Supplementary file 1 — gg4c00037_si_001.pdf [file gg4c00037_si_001.pdf]
